# Selective labeling and visualization of viral and bacterial neuraminidases using *ortho*-quinone methide-based probes

**DOI:** 10.1039/d5cb00170f

**Published:** 2025-10-01

**Authors:** Erianna I. Alvarado-Melendez, Simon T. Ruessink, Karin Strijbis, Tom Wennekes

**Affiliations:** a Department of Chemical Biology and Drug Discovery, Utrecht Institute for Pharmaceutical Sciences and Bijvoet Center for Biomedical Research, Utrecht University Utrecht The Netherlands t.wennekes@uu.nl; b Infection Biology Section, Division Infectious Diseases and Immunology, Department of Biomolecular Health Sciences, Faculty of Veterinary Medicine, Utrecht University Utrecht The Netherlands

## Abstract

Neuraminidases (NAs) are critical virulence factors in pathogens. In viruses such as influenza A, neuraminidase facilitates the release of virions, thereby enabling infection propagation. In pathogenic bacteria, NA activity has been linked to the pathogenicity of species such as *S. pneumoniae*, *P. aeruginosa*, and *V. cholerae*. Studies suggest that bacterial NAs play roles in mucus degradation, exposing host epitopes to enhance bacterial adhesion, biofilm formation, and bacterial survival. However, the specific mechanisms by which bacterial NAs contribute to pathogenesis remain poorly understood and largely unknown. To gain a deeper understanding of the molecular mechanisms underlying this class of enzymes, highly selective and sensitive strategies are needed for screening, detecting, and studying active NAs in complex biological samples. Specifically, chemical tools that can covalently label NAs without interfering with their enzymatic activity offer a powerful approach to precisely label and visualize these enzymes in their native environments. In this work, we present the development of novel *ortho*-quinone methide-based probes featuring an azide and biotin tags for the labeling and detection of NAs. These probes exhibit high selectivity in labeling recombinantly expressed NAs from influenza A virus and pathogenic Gram-negative *Prevotella* strains at nanomolar concentrations. Moreover, we developed a strategy that significantly improves labeling specificity of NAs when using our probes in complex samples, addressing the common issue of nonspecific labeling associated with quinone methide-based probes. Additionally, we demonstrate the potential of these probes for imaging extracellular NAs on bacterial surfaces, highlighting their utility for studying NAs in their native environments.

## Abstract 2

Highly selective *ortho*-quinone methide probes covalently label and visualize viral and bacterial neuraminidases, revealing enzyme activity and localization in complex samples with minimal off-target effects.

## Graphical abstract

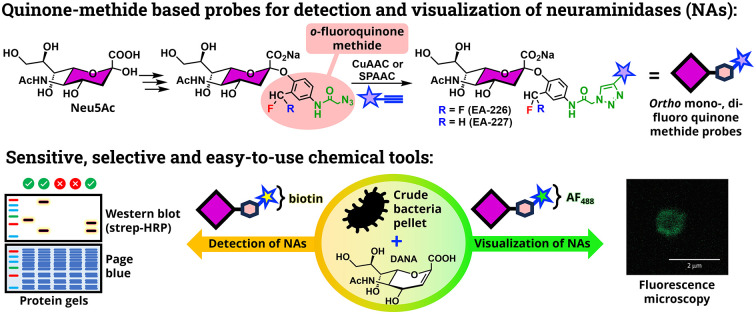

## Introduction

Neuraminidases (NAs), also known as exo-sialidases, are enzymes that hydrolyze terminal sialic acid residues from glycoconjugates. Sialylated glycoconjugates contribute to the glycocalyx, a dense layer on mammalian cell surfaces that supports barrier function and immune regulation.^1^ The removal of terminal sialic acids by viral and bacterial NAs compromises this barrier, facilitating infection and pathogen dissemination. In parallel, specific sialosides are recognized by microbial adhesins and receptors, promoting host cell attachment and invasion.^1^ The outcome of these processes is shaped by the substrate specificity, expression patterns, and localization of NAs during infection. In influenza A viruses, neuraminidases are essential for viral entry and dissemination. NAs are crucial for the release of newly assembled virions from host cells, allowing the infection to spread to neighboring cells.^2,3^ There is also evidence suggesting that NAs are important for viral entry into host cells by cleaving sialic acids from decoy receptors that can prevent the virus from binding to cell entry receptors.^4^ In bacteria, neuraminidases contribute to a variety of functions that promote survival and colonization. They are involved in the degradation of mucus in the respiratory, gastrointestinal, and reproductive tracts, providing bacteria with access to sialic acid as a nutrient source or building block.^5^ They also facilitate biofilm formation,^6^ unmasking host cell receptors, and enhancing bacterial adhesion by exposing host cell binding sites for adhesins and toxins.^7,8^ Notably, bacterial neuraminidases have been implicated in exacerbating influenza A infections by compensating for the inhibition of viral neuraminidases, thereby undermining the efficacy of neuraminidase-targeted antiviral therapies.^4^ Neuraminidases expression is not limited to pathogens; commensal bacteria in the respiratory and gastrointestinal tracts also express these enzymes.^9^ While the roles of commensal NAs in host-pathogen and host-microbiota interactions are still being elucidated, evidence suggests that they can influence mucosal environments by modifying mucin structures or unmasking host glycan epitopes to modulate immune responses.^9^

Given the widespread involvement of neuraminidases in infection and immune regulation, these enzymes are an important focus of research. Most neuraminidases associated with viruses and bacteria belong to the glycoside hydrolase families GH33 and GH34.^10^ These enzymes operate *via* a retaining two-steps catalytic mechanism in which a tyrosine residue acts as a nucleophile, forming a covalent bond with sialic acid.^11^ Hydrolysis of this covalent intermediate ultimately releases sialic acid, restoring the enzymatic activity.^12^ In contrast, some bacterial neuraminidases, such as those in GH58 and GH156, employ an inverting mechanism, in which water directly attacks the sialoside bond in a single displacement step, leading to inversion of stereochemistry at the anomeric carbon. As these enzymes are less well characterized,^13^ it is worthwhile developing tools that can be used for retaining and inverting sialidases.

A variety of chemical probes have been developed to label and study retaining neuraminidases.^14^ These include mechanism-based probes, such as carbocyclic mimics of Neu5Ac^15^ and difluoro-sialic acids,^16,17^ which act by trapping the covalent intermediate formed during the catalytic mechanism of retaining neuraminidases, thereby labeling and inhibiting the enzyme. While these probes facilitate enzyme detection and isolation, their utility is limited by the eventual hydrolysis of the trapped intermediate, and their relatively low sensitivity. We observed this limitation in experiments for the detection of neuraminidases in biological samples, which require concentrations ranging from 0.1 to 1.0 mM. These properties limit their use in samples where neuraminidase concentration is low, in experiments where a covalently stable label is required or when an inverting neuraminidase is involved.

**Scheme 1:**
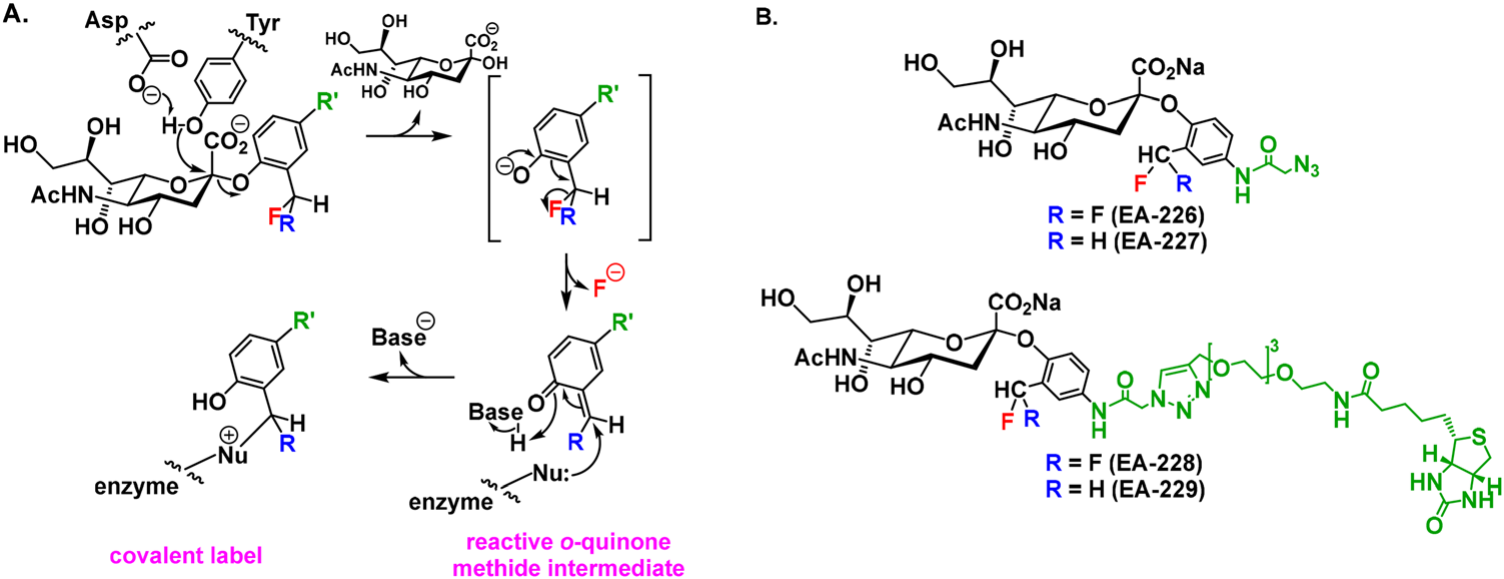
(A) Covalent labelling of neuraminidases with a quinone methide-sialoside. (B) Probes designed and developed in this work.

Fluorinated quinone methide-based substrates are an alternative strategy for the labeling and detection of neuraminidases. These probes work by a mechanism in which enzymatic cleavage of the quinone methide substrate generates a highly reactive *ortho*-quinone methide (see Scheme 1(A)). This electrophilic intermediate reacts rapidly with nucleophilic residues in the neuraminidase catalytic pocket or vicinities, effectively labeling active neuraminidases *in situ*. Compared to mechanism-based probes, quinone methide substrates can irreversibly modify the enzyme without affecting neuraminidase activity, allowing the enzyme to be labeled with multiple tags, thus providing signal amplification.^18^ Furthermore, the use of these probes is not limited to retaining neuraminidases but can also be used to label inverting neuraminidases.^13^

Quinone methide sialoside probes have previously been developed to both inhibit and label neuraminidases. The first report dates back to 2005 when Hinou *et al.* developed a fluorescent quinone methide probe to inhibit and visualize *Vibrio cholerae* neuraminidase.^19^ In the same year, Lu and collaborators reported the development of a quinone-methide-based probe to capture influenza A virus *via* its neuraminidases located at the cell surface.^20^ Later in 2013, Kai and co-authors reported the development of a macrocyclic quinone methide-based inhibitor designed to target eukaryotic, bacterial, and viral neuraminidases.^21^ However, the probe demonstrated limited efficacy in inhibiting these enzymes. In a follow-up study published in the same year, the same group reported the development of more potent neuraminidase inhibitors based on quinone methide-conjugated sialosides. They synthesized a library of probes based on a 2-difluoromethylphenyl sialoside bearing an aromatic azide group for aglycone functionalization, and they screened these probes for inhibitory activity against *Vibrio cholerae* neuraminidase (VCNA) and human neuraminidase 2 (*h*Neu2). Among the candidates, one probe exhibited the lower inhibition constant (*K*_i_) of 216 μM against VCNA, the most effective quinone methide-based substrate inhibitor reported to date.^22^ However, the overall inhibitory performance of these probes remained modest compared to other classes of neuraminidase inhibitors.

These studies showed that quinone methide sialosides can be used to label neuraminidases of bacterial and viral origin and have the potential to inhibit enzymatic activity. Importantly, the authors did not explore the use of the probes in complex biological samples. Despite their advantages as highly reactive molecules for labeling glycosyl hydrolases, quinone methide-based probes face challenges related to substrate selectivity. The reactive nature of the *ortho*-quinone methide intermediate, its relatively long lifetime and the associated diffusion can lead to off-target labeling, especially in complex biological samples, limiting their application in these systems.^23,24^

In this work, we report the development of four novel quinone methide-sialoside probes (Scheme 1(B)) that build upon previous designs^21^ to achieve improved selectivity and sensitivity for the detection and visualization of bacterial and viral neuraminidases *in vitro* and in complex biological matrices. We developed two classes of probes: both incorporate a 2-azidoacetamide handle, which offers enhanced stability and bioorthogonal reactivity compared to the prior aromatic azide tag. The first class bears a 2-difluoromethylphenyl group, while the second incorporates a 2-monofluoromethylphenyl group for which the enhanced quinone methide reactivity might enable faster and more controlled covalent labeling. We evaluated these probes for their ability to label and visualize retaining neuraminidases from the opportunistic vaginal bacterium *Prevotella timonensis*^25,26^ and influenza A virus.^27^ We demonstrate robust and selective labeling of neuraminidases using our probes, even within complex biological samples where off-target reactivity has historically limited quinone methide-based strategies. Furthermore, we anticipate that this approach could be extended to the labeling of inverting neuraminidases, as has been shown by others and us for similar probes on other inverting glycosidases.^13^

## Materials and methods

### Materials and general methods

All reagents were purchased from Merck, Thermo Fisher Scientific and Byosynth and were used without further purification. Organic solvents were dried for 24 h over pre-activated (2 h, 400 °C) 4 Å molecular sieves prior to use. Glassware for anhydrous reactions was flame-dried and cooled under a nitrogen atmosphere. Thin layer chromatography (TLC) was performed on aluminium-backed SiliaPlate TLC Plates F254 (Silicycle, Canada) and detected by UV (254 nm or 365 nm) where applicable and by dipping in 10% sulfuric acid in ethanol, *p*-anisaldehyde sugar stain or ceric ammonium molybdate stain followed by heating. Analytical thin layer chromatography (TLC) was performed on glass-backed TLC plates pre-coated with silica gel (60G, F_254_). Column chromatography was carried out using silica gel (40–63 μm; VWR chemicals) or C18-reversed phase silica gel (40–63 μm; Merck). Solvents were evaporated at maximum 40 °C under reduced pressure.

Nuclear magnetic resonance (NMR) spectra were recorded on a 400 MHz Agilent spectrometer (400 and 101 MHz) or a 600 MHz Bruker Avance Neo spectrometer (600 and 125 MHz). Chemical shifts are reported in parts per million (ppm) relative to residual solvent peak. Mass spectra were recorded by ESI on a Bruker micrOTOF-QII mass spectrometer.

Expression and purification of the recombinant proteins N1WIS from influenza A and *Prevotella timonensis* neuraminidases NanH1 and NanH2 (*Pt*NanH1 and *Pt*NanH2) were carried out as reported previously.^25,27^

### Bacterial strains and growth conditions:

*P. timonensis* and *P. bivia* were grown at 37 °C under anaerobic conditions (5% H_2_, 10% CO_2_, 85% N_2_) in a Coy Lab's Vinyl Anaerobic Chamber. Bacteria were cultured in Cooked Meat Medium (CMM, CM0081) supplemented with 1 mg L^−1^ vitamin K1 (Sigma, V3501) and 5 mg L^−1^ hemin (Sigma, 51280) following the DSMZ #110 protocol (https://www.dsmz.de/microorganisms/medium/pdf/DSMZ_Medium110.pdf).

### Neuraminidase activity assays

Neuraminidase activity was measured by the fluorometric assay using 4-methylumbelliferyl *N*-acetyl-α-d-neuraminic acid sodium salt (MUNANA, Sigma Aldrich) as the substrate. The activity of the recombinantly expressed enzymes was determined by measuring hydrolysis of MUNANA. For this, the reactions were set up in 96-well plates by adding 400 μM of MUNANA to the recombinant enzyme (0.04 μM *Pt*NanH1, and 0.04 μM *Pt*NanH2) in bis-tris buffer (50 mM, 4 mM CaCl_2_, pH 6) for a total volume of 100 μL. The concentration of bis-tris buffer used did not affect the activity of the enzymes as shown in S8. Fluorescence was measured after addition of MUNANA at room temperature (RT) overtime (340 nm excitation and 490 nm emission) with a gain of 1124 (CLARIOstar Plus).

For the inhibition assays, the enzyme solutions (0.02 μM *Pt*NanH1, and 0.01 μM *Pt*NanH2) were pre-incubated with varying concentrations of EA-229 (500 μM–0.5 pM) for 30 minutes at RT. Neuraminidase activity was assessed by adding MUNANA (200 μM, 100 μL total volume on well), and measuring the fluorescence overtime at 340 nm (excitation) and 490 nm (emission) with a gain of 1124 (CLARIOstar Plus).

### Labeling experiments

The recombinant neuraminidase N1WIS was incubated with different concentrations of EA-226, EA-227, EA-228 and EA-229 in bis-tris buffer (50 mM, 4 mM CaCl_2_, pH 6) at room temperature for 1 hour. Samples treated with *E. coli* lysate were prepared by adding 1 μL of *E. coli* cell lysate (1 mg mL^−1^) to the N1WIS solutions in bis-tris buffer, followed by the addition of the probes. After incubation, the samples were denatured with loading buffer and boiling at 95 °C for 5 min. Samples treated with probes EA-226 and EA-227 were denatured with a non-reducing Laemmli buffer. Samples treated with EA-228 and EA-229 were denatured with Laemmli buffer.

Labeling of different neuraminidases was tested by incubating the enzymes (1 μM final concentration) incubated with EA-229 (0.5 μM) for 1 h at RT. Solutions of PtNanH1, *Pt*NanH2, TcTS, VCNA, N1WIS and NANA aldolase were prepared in bis-tris buffer (50 mM, 4 mM CaCl_2_, pH 6), the solution of Kdnase was prepared in sodium acetate buffer (50 mM, pH 4) the optimal pH for its activity. When bacterial pellets were used, 1 μL of loose pellet suspended in 19 μL of bis-tris buffer was used as the source of enzyme. For the competition assays, the enzymes/bacterial samples were preincubated with the inhibitor (DANA or diF-Neu9AF_647_) for 30 min at RT, followed by incubation with the probe EA-229 for 1 h at RT.

### SDS-PAGE and western blot analysis

After incubation with the probes, the samples were denatured using Laemmli buffer. For the experiments with EA-226 and EA-227, a non-reducing loading buffer was used to prevent reduction of the azido group. Then samples were heated at 95 °C for 5 minutes. Proteins were separated by SDS-PAGE using 10% bis-tris gels system under denaturing condition (120 V, 60 minutes). For the western blotting, the gel was electroblotted onto a PVDF membrane. The membrane was blocked with 5% skimmed milk for 30–60 min, washed with 1% milk for 5 min, stained with anti-biotin-HRP antibody (1:10 000 in 1% milk), washed (1% milk, followed by PBS, 5 min each), and treated with ECL Western substrate (Bio-Rad Laboratories) for signal detection. Staining of protein gels was done using PageBlue™ Protein Staining Solution (Thermo Fisher Scientific). In-gel fluorescence was imaged using Cytiva® Imaging System on the Cy5 channel (625–650 nm excitation, 675–725 nm emission filter).

### Fluorescence confocal microscopy

*Prevotella timonensis* CRIS 5C-B1 (*circa* 1 × 10^8^ cells) were centrifuged at 4000 rpm for 5 min at 4 °C, washed two times with PBS, and resuspended in 20 μL of PBS. Controls with the non-covalent inhibitor DANA were treated for 30 min prior addition of EA-227. Incubation with EA-227 was performed for 1 h at RT. After this, cells were collected by centrifugation (4000 rpm, 5 min, RT) and washed two times with PBS. Pellets were resuspended in 20 μL of click mixture: 1 μM alkyne-Alexa Fluor 488 (Jena Bioscience, Jena, Germany), 0.5 mM CuSO_4_, 2.5 mM Na-l-ascorbate in PBS, or PBS for the controls. Samples were then incubated in the dark for 2 h at RT. Bacteria were centrifuged (4000 rpm, 5 min, RT), washed two times with PBS and resuspended in 900 μL of a solution 2% PFA in PBS to fix the cells. Samples were incubated for 30 min at RT in the dark. Fixation was stopped by addition of 400 μL of a 50 mM solution of NH_4_Cl in PBS. After which bacteria pellets were washed two times with PBS, and carefully resuspended in 10 μL of ProLong™ Diamond Antifade Mountant (Thermo Fisher Scientific) and mounted on a glass slide. Slides were stored at RT overnight to allow the samples to harden. Images were collected on Olympus/Evident SpinSR10 confocal microscope in combination with SORA software. Image analysis was performed using OlyVIA and ImageJ software.

### Synthesis and characterization

**Fig. 1:**
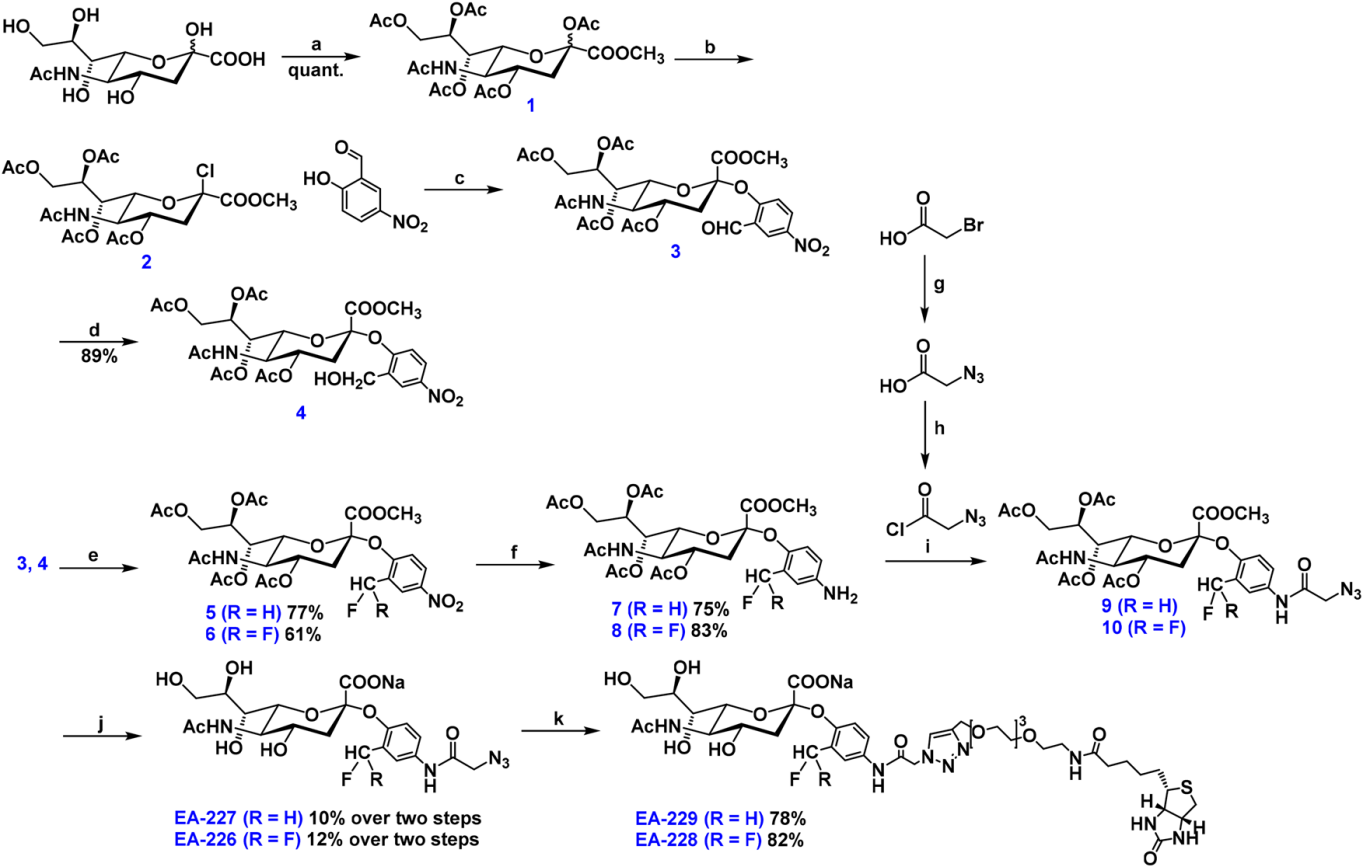
Synthesis of the quinone methide sialoside probes. Reagents and conditions: (a) 1. Amberlite-H^+^ resin, CH_3_OH, room temperature (RT), overnight (o/n), 2. Ac_2_O, pyridine, RT, o/n; (b) AcCl, HCl (g), 0 °C to RT, o/n; (c) 2, 2-hydroxy-5-nitrobenzaldehyde (1.1 eq.), DIPEA, CH_3_CN, RT, o/n; (d) 3, NaB(OAc)_3_H (10 eq.), EtOH, 0 °C to RT, o/n; (e) 3 or 4, DAST (6 eq. 3 eq.), DCM, 0 °C, 4 h or 2 h; 5 or 6, Pd/C, H_2_ (g), EtOAc, RT, 6 h or 12 h; (g) NaN_3_; (h) oxalyl chloride, DCM, 0 °C, o/n; (i) 7 or 8, 2-azidoacetyl chloride (5 eq.), pyridine, DCM, 0 °C to RT, o/n; (j) 1. 9 or 10, Na_2_CO_3_ (3.3 eq.), CH_3_OH, RT, 3 h, 2. NaOH (aq.), pH 11, RT, o/n; (k) EA-226 or EA-227, alkyne-PEG_4_-biotin (2.5 eq. or 1.5 eq.), Cu-THPTA, Na-l-ascorbate, H_2_O/THF, RT, 16 h.

We started the synthesis of the probes by preparing intermediates 3 and 6 (Fig. 1), using a protocol adapted from the literature.^19,21^ For probes featuring a difluoromethyl substituent on the aromatic ring of the aglycone (EA-226 and EA-228), compound 3 was treated with excess DAST to fluorinate the aldehyde, yielding intermediate 6. For the synthesis of EA-227 and EA-229, the aldehyde in compound 3 was first reduced to the corresponding alcohol, producing intermediate 4, which was then fluorinated with DAST to give compound 5. Due to the susceptibility of intermediates 5 and 6 to hydrolysis under both acidic and basic conditions, as well as elevated temperatures, from this point on all reactions and workups were performed at 0 °C or RT. Reduction of the nitro groups in 5 and 6 was achieved using atmospheric pressure hydrogen and Pd/C, affording intermediates 7 and 8, respectively. Separately, 2-azidoacetyl chloride was synthesized according to a reported method^28^ and subsequently coupled to 7 and 8, to obtain compounds 9 and 10. Despite our attempts to prevent hydrolysis of the products during the washing steps and evaporation of the solvent, we still recovered a mixture of product and the anomerically hydrolyzed product. We continue with the deprotection steps using the crude mixtures. EA-226 and EA-227 were purified using reverse-phase column chromatography. Finally, CuAAC reaction was used to conjugate alkyne-PEG_4_-biotin, affording the target probes EA-228 and EA-229. All intermediates were characterized by ^1^H-NMR, ^13^C-NMR, ^19^F-NMR and ESI-MS. The synthesis and the characterization data can be found in S1.

## Results and discussion

We synthesized four quinone methide-based sialic acid probes differing in reactivity and functional handles: EA-226 and EA-227 contain an azidoacetamide group in the aglycone, whereas EA-228 and EA-229 are biotin-functionalized (Scheme 1(A)). To evaluate how their reactivity affects labeling of recombinant and bacterial neuraminidases, we introduced a difluoromethyl substituent into the aglycone aromatic ring of EA-226 and EA-228, while EA-227 and EA-229 contain a monofluoromethyl substituent (Fig. 1). The difluoromethyl probes (EA-226 and EA-228) are expected to exhibit reduced reactivity compared to their monofluoromethyl counterparts (EA-227 and EA-229), due to electrostatic stabilization by the additional fluorine atom (Scheme 1(A)). Additionally, based on prior work, the rate of fluorine elimination and subsequent quinone methide quenching by nucleophiles should be slower for difluoromethyl-containing probes (EA-226, EA-228) relative to the monofluoromethyl analogs (EA-227, EA-229).^28,29^ This slower reactivity may increase the chances of off-target labeling, as the reactive intermediate persists longer and can diffuse further before being quenched.

**Fig. 2:**
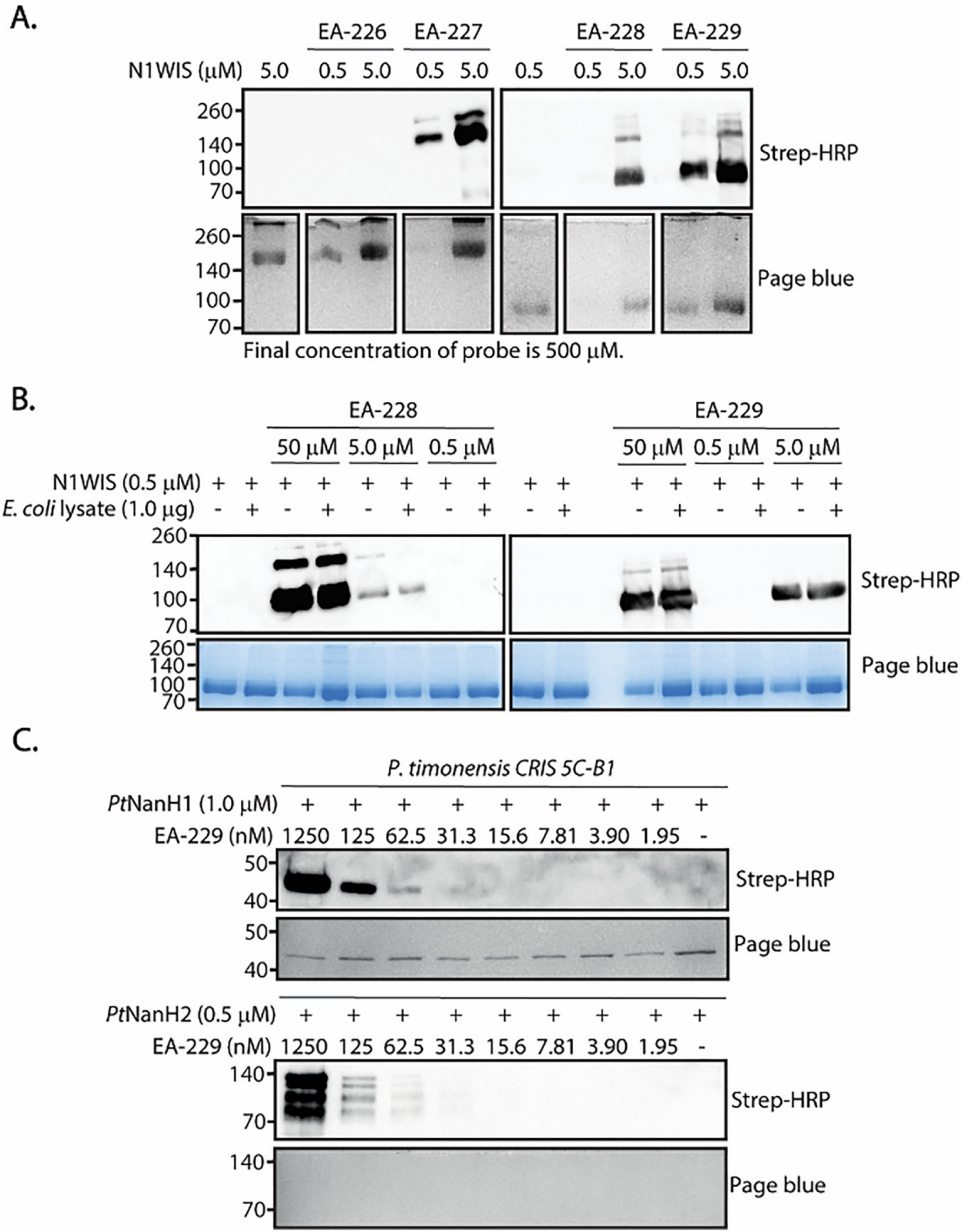
(A) Labeling of N1WIS neuraminidase from influenza A virus using EA-226, EA-227, EA-228 and EA-229 (500 μM final concentration). (B) Limit of detection and selectivity of EA-228 and EA-229 incubated with N1WIS (70 kDa) in the presence of a *E. coli* lysate at different concentrations. (C) Labeling of recombinant *Pt*NanH1 (44 kDa) and *Pt*NanH2 (110 kDa) from *Prevotella timonensis* CRIS 5C-B1 with EA-229 at different concentrations.

We first evaluated the ability of EA-226 and EA-227 to label N1WIS influenza A virus neuraminidase by incubating the probes (500 μM final concentration) with the recombinant enzyme for one hour at RT. Samples were denatured under non-reducing conditions and analyzed by SDS-PAGE and western blot. Visualization of the bands was achieved by a copper-catalyzed azide–alkyne cycloaddition (CuAAC) reaction on the blot with alkyne-biotin, followed by incubation with streptavidin-HRP and enhanced chemiluminescence substrate (ECL). The EA-227-treated sample displayed two distinct bands at approximately 280 and 140 kDa, corresponding to the dimeric and tetrameric forms of the neuraminidase, respectively. These signals were noticeably more intense than those observed in the EA-226-treated sample, which can only be detected when we overexposed the blot (Fig. 2(A), for full gel images and overexposed blots see S2, SI), suggesting more efficient labeling of the monofluoromethyl-containing probe. Since both EA-226 and EA-227 have phenolic aglycones with a p*K*_a_ around ∼9, weak leaving groups, glycosylation of the neuraminidase to the covalent intermediate is rate-determining.^30,31^ The stronger inductive effect of the difluoromethyl substituent in EA-226 lowers the p*K*_a_ of its phenolic aglycone compared to EA-227, which should enhance leaving-group ability and thus accelerate aglycone release. However, the reduced labeling efficiency of EA-226 indicates that quinone methide formation is the crucial step. Upon enzymatic hydrolysis of the glycosidic linkage, the *ortho*-di or mono-fluoromethyl-phenolate released undergoes a 1,4-elimination, generating the highly reactive quinone methide intermediate (Scheme 1(A)). The difference in the labeling degree when using EA-226 and EA-227 correlates with what was previously observed for these mono- or di-fluorinated quinone methide precursors, as stated above.

Upon enzymatic hydrolysis of the glycosidic linkage, the *ortho*-difluoromethyl-phenolate released undergoes a 1,4-elimination, generating a highly reactive quinone methide intermediate (Scheme 1(A)). The difference in the labeling degree when using EA-226 and EA-227, can be a result of the different reactivities of the probes and diffusion rates of the reactive intermediates, as stated above. Another possibility for the lower degree of labeling by the difluoromethyl containing EA-226 is that after the formation of the covalent intermediate with the enzyme (Scheme 1) the phenol group of the conjugate forms a quinone methide by elimination of the remaining fluorine atom. This reactive intermediate can react with water to form a hemiacetal that in turn can be converted into the aldehyde with concomitant loss of the reporter group's covalent bond to the enzyme.^28^

We next performed experiments with the biotinylated probes EA-228 and EA-229 and similar results were obtained. In these experiments, the samples were denatured under reducing conditions and detected using streptavidin-HRP and enhanced chemiluminescence substrate (ECL). In this case, only the monomer and dimer of the N1WIS NA were detected. As EA-228 and EA-229 allow us to visualize the N1WIS NA monomers and are easier to use, we tested the selectivity of the probes towards N1WIS in the presence of an *E. coli* cell lysate. As shown in Fig. 2(B), the probes selectively labeled the viral neuraminidase at concentrations as low as 5.0 μM.

**Fig. 3:**
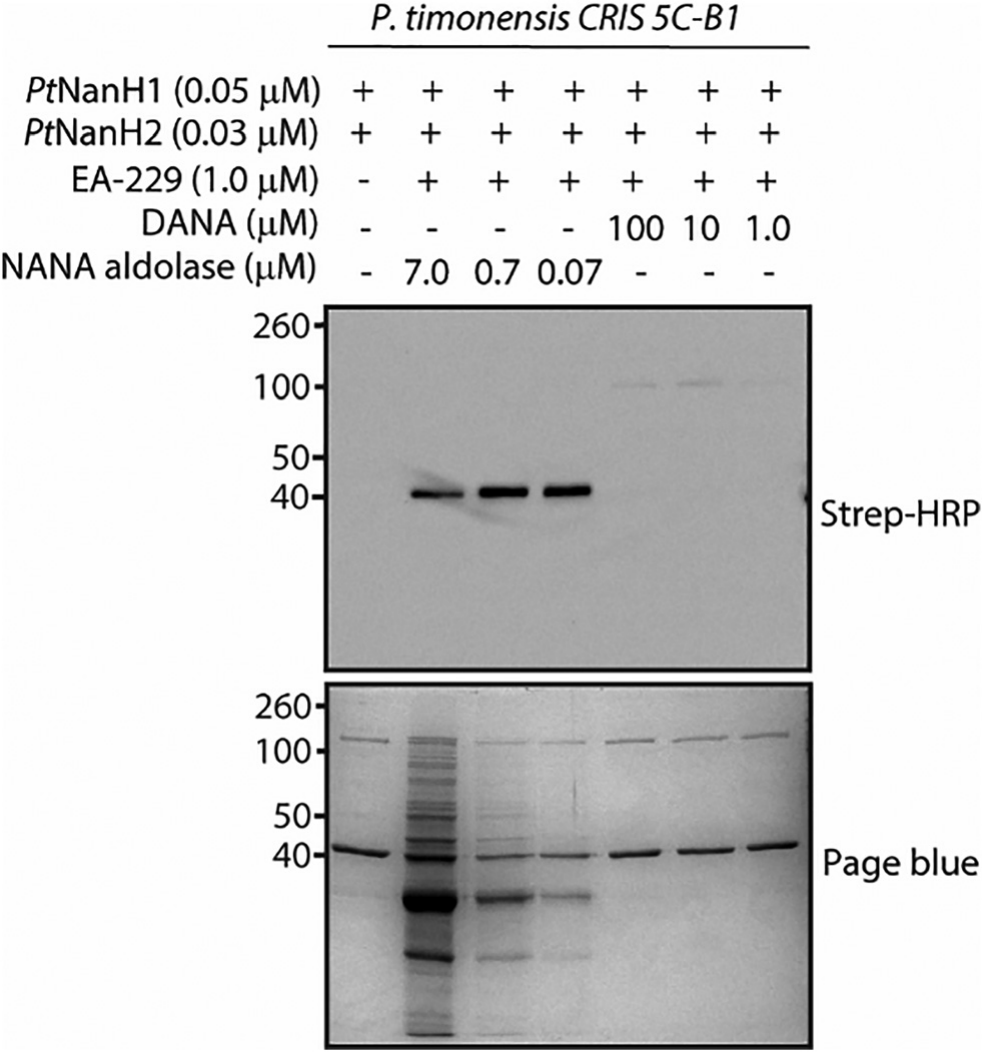
Labeling of *Pt*NanH1 (44 kDa) and *Pt*NanH2 (110 kDa) in the presence of NANA aldolase (35 kDa) and competition assay with the neuraminidase inhibitor DANA.

**Fig. 4:**
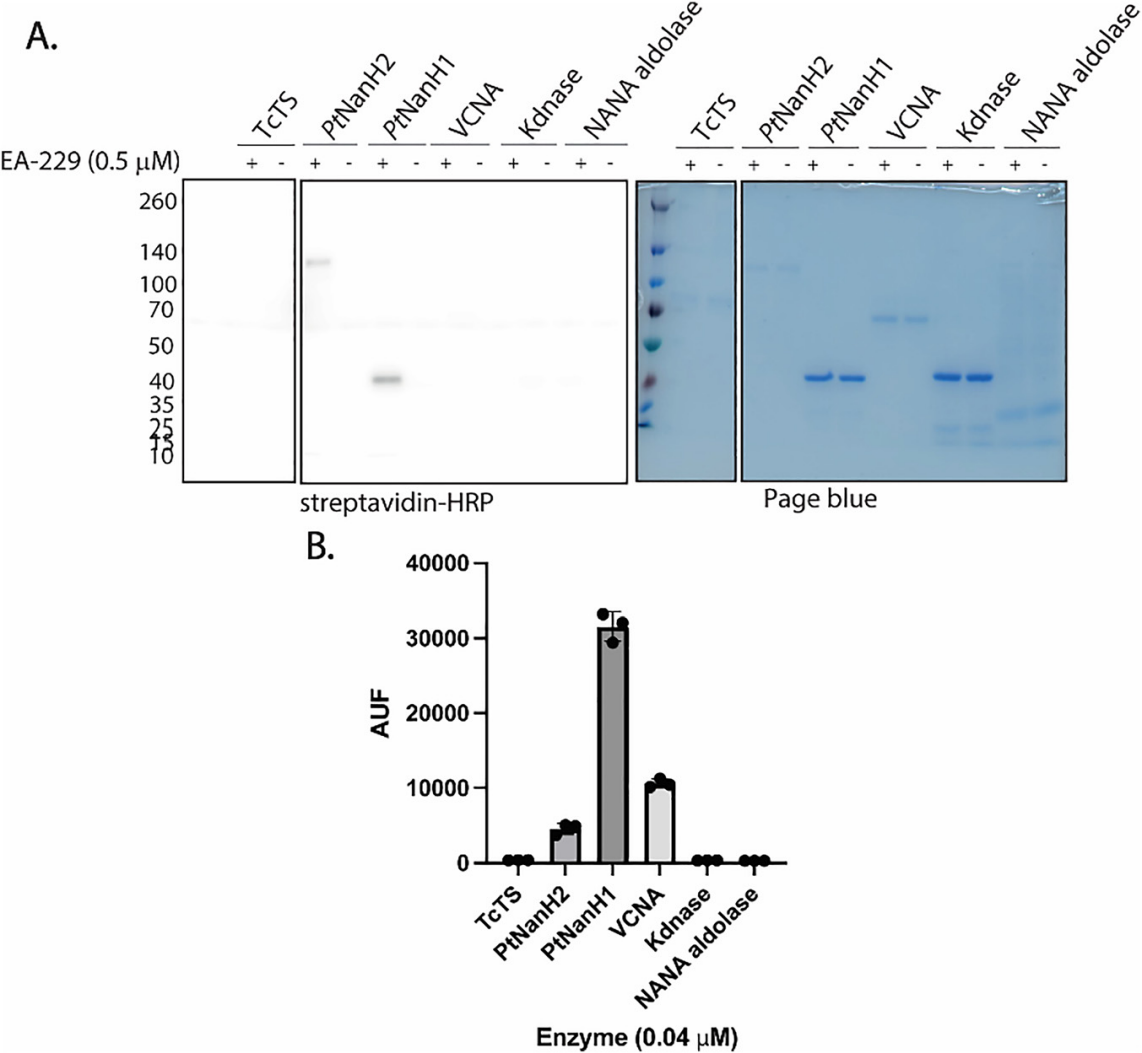
(A) *In vitro* labeling of different enzymes using EA-229. TcTS (1.0 μM, 144 kDa), *Pt*NanH2 (1.0 μM, 110 kDa), *Pt*NanH1 (1.0 μM, 44 kDa), VCNA (0.1 U, 95 kDa), Kdnase (1.0 μM, 46 kDa), NANA aldolase (1.0 μM, 35 kDa). (B) Fluorometric assay for neuraminidase activity of recombinant neuraminidases using MUNANA. Enzyme solutions (0.04 M) were incubated with 400 M MUNANA at RT. Fluorescence was measured after 10 minutes of the MUNANA addition (340 nm excitation and 490 nm emission) with a gain of 1124 (CLARIOstar Plus). Data plotted is the result of three replicates.

Next, we used EA-229, which showed to be the most sensitive and easy-to-use probe for the labeling of influenza A neuraminidase, to label recombinant bacterial neuraminidases from *Prevotella timonensis*. As shown in Fig. 2(C), EA-228 and EA-229 can label 1 μg of *Pt*NanH1, and *Pt*NanH2 at concentrations as low as 62 nM. We also show that EA-229 selectively labels the bacterial neuraminidase NanH1, in the presence of nonspecific competitor protein, the commercially available NANA aldolase. We confirmed that the observed labeling is neuraminidase activity-dependent with a competition assay where the neuraminidases were pre-incubated with DANA, a known non-covalent neuraminidase inhibitor (Fig. 3). The difference in labeling between *Pt*NanH1 and *Pt*NanH2 can be attributed to their intrinsic catalytic activities towards the probe. To evaluate this, we performed a fluorescence-based assay using 2′-(4-methylumbelliferyl)-α-d-*N*-acetylneuraminic acid (MUNANA). When a neuraminidase hydrolyzes MUNANA, the release of 4-methylumbelliferone (4-MU) is proportional to the hydrolysis rate and can be monitor by fluorescence. As shown in Fig. 4(B), consistent with the labeling results, *Pt*NanH1 exhibits higher enzymatic activity than *Pt*NanH2.

In the competition assay using DANA, we observed abrogation of *Pt*NanH1 labeling and faint bands for *Pt*NanH2 that were not visible in the absence of the inhibitor (Fig. 3). One possible explanation is that both enzymes, *Pt*NanH1 and *Pt*NanH2, compete for the substrate, due to its higher catalytic efficiency, *Pt*NanH1 hydrolyzes the probe more rapidly, reducing its availability and thereby limiting the effective labeling of *Pt*NanH2. Upon the addition of DANA, a more potent inhibitor of *Pt*NanH1 (see S2, SI), **EA-229**-mediated labeling of *Pt*NanH1 is suppressed. At the tested concentrations, DANA does not inhibit the activity of *Pt*NanH2 (S3, SI). This enzyme is then exposed to **EA-229** for long enough to result in weak labeling (Fig. 3). For clarity, we refer to the recombinant neuraminidases from *Prevotella timonensis* CRIS 5C-B1 as *Pt*NanH1 and *Pt*NanH2, and to the native proteins present in the bacterial cells as NanH1 and NanH2.

We investigated whether labeling by EA-229 affects enzyme activity with a fluorometric inhibition assay. For this, the recombinant enzymes *Pt*NanH1 and *Pt*NanH2 were preincubated with EA-229. DANA was also used as a positive control. Next, the fluorescent substrate MUNANA was added to the samples, and the fluorescence was measured after 1 h incubation at 37 °C. As expected, we found that EA-229 did not inhibit the enzyme activity at the concentrations tested in this assay (1 μM) (see S4, SI).

The specificity of EA-229 was further evaluated by incubating the probe with different recombinant enzymes, the *Trypanosoma cruzi trans*-sialidase (TcTS, 144 kDa), a Kdnase from the fungus *Aspergillus fumigatus* (46 kDa), the commercially available *Vibrio cholerae* neuraminidase (VCNA, 95 kDa), recombinant neuraminidases from *P. timonensis Pt*NanH1 (44 kDa) and *Pt*NanH2 (110 kDa); and finally, the sialic acid aldolase NANA (35 kDa) as a negative control. For this experiment, the enzyme concentration was fixed at 1.0 μM and the probe concentration was 0.5 μM. Fig. 4(A) shows that EA-229 preferentially labels *Pt*NanH1 and *Pt*NanH2 under these conditions. We also demonstrate that the probe selectively targets neuraminidases, as the NANA aldolase and the Kdnase were not labelled. Since the active sites of the neuraminidases *Pt*NanH1, *Pt*NanH2 and VCNA are similar and conserved, we hypothesize that the difference in selectivity could be related to the intrinsic catalytic activity of the enzymes. To verify this, we measured the relative activities of the enzymes with a MUNANA assay (Fig. 4(B)). We maintained the relative concentrations of the enzymes at levels equivalent to those used in the labeling experiment. The relative activities of *Pt*NanH1 and *Pt*NanH2 align with what is observed in Fig. 4(A). In the MUNANA assay, the fluorescent substrate contains a smaller aglycone group compared to EA-229. This structural difference likely affects how the enzyme interacts with and hydrolyzes the substrates. Therefore, we hypothesize that the lack of visible labeling of VCNA by EA-229 is due to differences in how VCNA interacts, or processes EA-229 compared to *Pt*NanH1 and *Pt*NanH2, suggesting that EA-229 exhibits a degree of selectivity toward *Pt*NanH1 and *Pt*NanH2. We hypothesize that this observed selectivity arises from differences in hydrolysis rates among the enzymes, with slower-catalyzing enzymes such as TcTS generating the quinone-methide intermediate less efficiently, thereby limiting labeling.

We next sought to determine if our probes were capable of labeling neuraminidases on the surface of intact *Prevotella* bacterial cells. We selected this system due to the relevance of neuraminidases in *Prevotella* timonensis strains, which have been associated with bacterial vaginosis.^25^

**Fig. 5:**
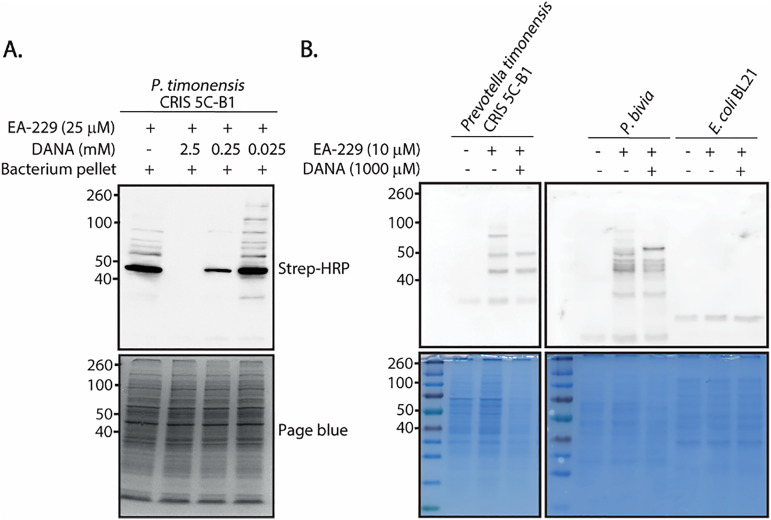
(A) *In vitro* labeling of neuraminidases from bacterial cells. The expected sizes of NanH1 and NanH2 are 47 and 110 kDa, respectively. A solution of 1 μL of bacterial pellet in 19 μL of bis-tris buffer (50 mM, 4 mM CaCl_2_, pH 6) was preincubated with DANA for 30 min at RT, followed by addition of EA-229 and incubation for 30 min. The resulting samples were denatured and analyzed by western blot. (B) Labeling of different bacterial pellets preincubated with DANA using EA-229, *E. coli* BL21 pellet was used as negative control as this bacterium does not express neuraminidases.

We cultured *P. timonensis* CRIS 5C-B1 under anaerobic conditions, pelleted and washed with PBS, followed by incubation with EA-229, SDS-PAGE and western blot analysis. In this assay, we included samples that were preincubated with the neuraminidase inhibitor DANA to verify that the observed labeling was neuraminidase-dependent. As illustrated in Fig. 5(A), the *P. timonensis* CRIS 5C-B1 sample treated with EA-229 exhibits multiple intense bands, while the samples preincubated with DANA show a decrease in number and intensity of the bands with increasing concentrations of the inhibitor.

To prevent off-target labeling, we performed the experiments under different conditions such as lower concentrations of EA-229, incubation at 4 °C instead of 37 °C, and addition of Tritonx100 (1% v/v). These attempts did not reduce the observed unspecific labeling (see S5, SI). However, in the competition experiment with DANA (Fig. 5(A)), to our surprise, we did observe reduced unspecific labeling and a clear predominant band between 40 and 50 kDa. This enhanced labeling occurred when DANA was used in a 10-fold molar excess compared to the probe, and the band disappeared when DANA was added in a 100-fold molar excess. We hypothesize that adding DANA at a 10- to 100-fold molar excess modulates probe turnover by the enzyme, controlling the release of quinone methide reactive intermediates to a degree that enables selective and clean labeling of NanH1 in the bacterial pellet. Another possibility is that the interaction of the enzyme with DANA exposes or enhances the nucleophilicity of a specific amino acid residue in the vicinities of the catalytic pocket, allowing it to react more efficiently with the quinone methide intermediate. We are currently conducting mass spectrometry studies to identify the attachment sites of the novel quinone methide probe on the *Pt*NanH1 enzyme, as well as to determine the site of modification and to confirm the identity of the protein labeled in the bacterial pellet.

In the labeling experiments using the *P. timonensis* CRIS 5C-B1 pellet, we did not observe a band for NanH2. This finding is consistent with the observation that *Pt*NanH1 exhibits higher activity than *Pt*NanH2 in the MUNANA assay. Taken together, these results could indicate a difference in the enzyme expression levels on the bacterial surface. Alternatively, it is possible that EA-229 is more efficiently recognized or processed by NanH1 than by NanH2.

We used the same strategy of combining a 100-fold excess of DANA and EA-229 to label neuraminidases from cells of other *Prevotella* strains. For these experiments we included *P. timonensis* CRIS 5C-B1, and *Prevotella bivia*. Preincubating the cells with a 1:100 excess of DANA over EA-229, we observed less labeled proteins compared to samples treated with EA-229 alone (Fig. 5(B)). In particular, for *P. bivia*, without preincubation with DANA, we did not observe a clear band for its neuraminidase NanH (60 kDa). However, in the samples pretreated with DANA, an intense band appears at 60 kDa.

We next conducted a competition experiment using a covalent inhibitor, the fluorescent difluorosialic acid probe diF-Neu9AF_647_, to determine whether we could replicate the effect previously observed with the non-covalent inhibitor DANA. In addition to assessing competition, we aimed to explore whether dual labeling of neuraminidases in the bacterial pellet was possible by combining EA-229 with diF-Neu9AF_647_.

**Fig. 6:**
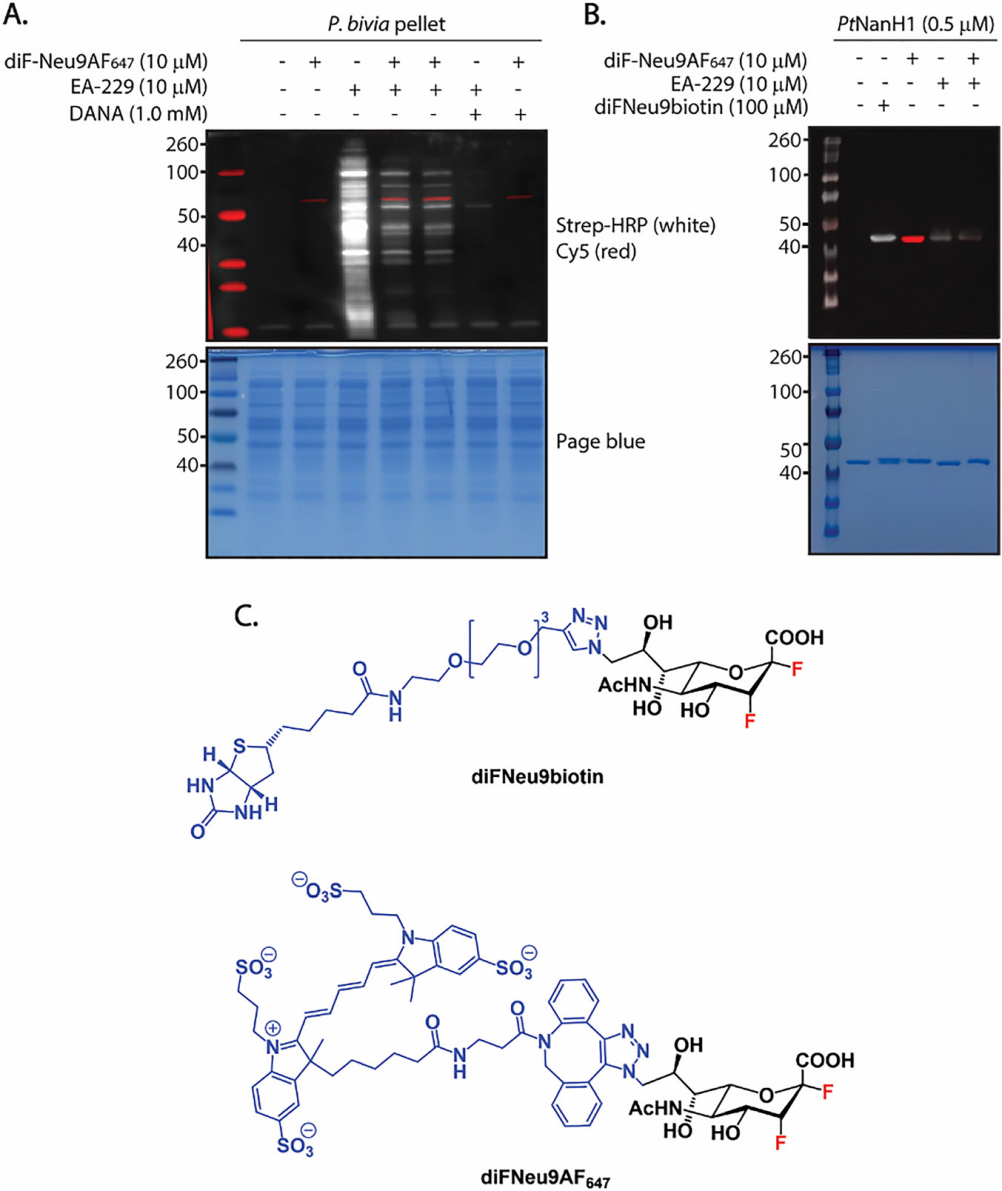
Overlapped gel images, in red Cy5 fluorescence signals, in white signals obtained by the ECL substrate reaction with the streptavidin-HRP tag. (A) A solution of 1 μL of bacterial pellet in 19 μL of bis-tris buffer (50 mM, 4 mM CaCl_2_, pH 6) was preincubated with DANA for 30 min at RT, followed by addition of EA-229 and incubation for 30 min. The resulting samples were denatured and analyzed by western blot. (B) Labeling of *Pt*NanH1 with the fluorescent difluoro sialic acid probe diFNeu9AF_647_, diFNeu9biotin and EA-229. (C) Chemical structure of diFNeu9biotin and diFNeu9AF_647_.

*P. timonensis* CRIS 5C-B1 bacterial pellet was incubated simultaneously with equal concentrations of both probes EA-229 and diF-Neu9AF_647_. To determine whether we could reproduce the effect seen in the DANA competition assay, we also included a sample where the bacterial pellet was preincubated with diF-Neu9AF_647_ for 30 minutes prior to incubation with EA-229. In contrast to EA-229, diF-Neu9AF_647_ forms a covalent intermediate in the catalytic site of the enzyme, thereby inhibiting its activity. Interestingly, we did not observe a band corresponding to the doubly labeled enzyme. As shown in Fig. 6(A), two bands between 40 and 50 kDa are visible, a red signal from the diF-NeuAF_647_ probe, and a slightly lower band detected with the HRP-streptavidin antibody. Samples pretreated with diF-Neu9AF_647_ exhibited reduced off-target labeling, indicating that the selectivity enhancement effect obtained with DANA can also be reproduced with a covalent inhibitor. This further suggests that the effect is related to interactions of the inhibitors with the catalytic pocket. We are currently performing proteomics experiments to elucidate this mechanism. The two bands observed correspond to two different population of enzymes: one bearing a biotin group and the other labeled with the fluorescent diF-Neu9AF_647_ (Fig. 6(A)).

The difference in protein migration between the main bands labeled by EA-229 and diF-Neu9AF_647_ could be attributed to the tags introduced by the probes. EA-229 adds one or multiple tags of 680 Da, while diF-Neu9AF_647_ forms a covalent intermediate with the enzyme, adding 1447 Da to the molecular weight of NanH1. To assess whether the different labels affect protein migration in SDS-PAGE, we incubated the recombinant *Pt*NanH1 with both EA-229 and diF-Neu9AF_647_ and analyzed the mixture by SDS-PAGE (Fig. 6(B)). *Pt*NanH1 labeled by EA-229 migrates slightly faster than the enzyme treated with diF-Neu9AF_647_, demonstrating that the different tags influence protein migration and may explain why the bands observed in Fig. 6(A) do not colocalize.

To investigate why we did not observe neuraminidases labeled by both probes when they were incubated simultaneously with EA-229 and diF-Neu9AF_647_, we assessed whether EA-229 might inhibit enzymatic activity and thereby preventing subsequent labeling by diF-Neu9AF_647_. To test this, we performed a MUNANA assay using the *P. timonensis* CRIS 5C-B1 bacterial pellet as the enzyme source. The results confirmed that neuraminidases in the **EA-229**-treated bacterial pellet remained active (S6, SI). This indicates that the enzyme population initially labeled by EA-229 retains catalytic activity and, in principle, remains accessible for labeling by diF-Neu9AF_647_. The absence of dual labeling may therefore result from steric hindrance introduced by the biotin tags near the active site. While this steric effect does not appear to interfere with turnover of the small MUNANA substrate, allowing activity to be detected, it may impede access or processing of the bulkier diF-Neu9AF_647_ probe. Consequently, we do not observe distinct bands corresponding to doubly labeled neuraminidases under these conditions.

We cannot rule out the possibility that the two bands labeled in Fig. 6(A) correspond to different protein species. Planned application of these probes in proteomic experiments should confirm their identities, but our results here suggest that the type of label contributes to the observed protein migration patterns. As shown in Fig. 6(B), labeling of *Pt*NanH1 with different probes produces shifts in migration, with diF-Neu9biotin and diF-Neu9AF_647_ migrating slightly more slowly than both the unlabeled protein and the EA-229-labeled protein.

**Fig. 7:**
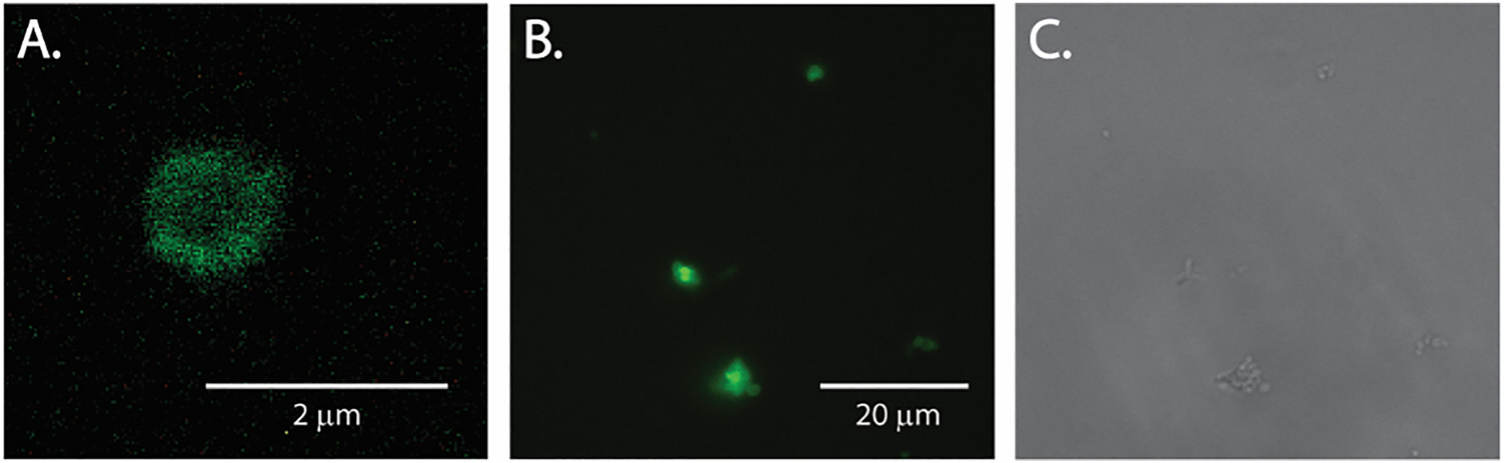
(A) High resolution fluorescence microscopy image of a *P. timonensis* CRIS 5C-B1 cell fluorescently labeled using EA-227 followed by CuAAC reaction with alkyne-AF_488_. This image was collected on an Olympus/Evident SpinSR10 confocal microscope in combination with SORA software. (B) Image collected at a lower resolution using an EVOS 5000 microscope (100×). (C) Transmitted-light image, EVOS 5000 microscope (100×). Images of unlabeled and non-specific binding controls are provided in Fig. S7 (SI).

To demonstrate the utility of the quinone methide probes for imaging of cell surface neuraminidases, we incubated EA-227 with *P. timonensis* 5C-B1 bacterial cells, followed by CuAAC reaction with alkyne-AF_488_ to visualize the cells. As a negative control bacterial cells were preincubated with DANA. The labeling with EA-227 resulted in a bright green signal in the bacterial cell surface (Fig. 7). This fluorescent signal was reduced when the samples were preincubated with DANA, confirming that the labeling was neuraminidase-dependent (S7, SI).

## Conclusion

We developed four novel quinone methide-based probes for the detection and visualization of viral and bacterial neuraminidases. The probes are sensitive and enable detection of neuraminidase activity without interfering with enzymatic function. EA-227 was successfully applied to image neuraminidases on the bacterial surface of *P. timonensis* cells with precision and sensitivity. Furthermore, we demonstrate selective labeling of neuraminidases in complex biological samples using a combination of EA-229 and a neuraminidase inhibitor. This strategy can be extended to complex co-culture systems for sensitive and selective detection and visualization of neuraminidases. We envision that these novel probes will support the discovery, labeling, and functional characterization of retaining and inverting neuraminidases in clinically relevant pathogens.

## Author contributions

E. I. A. M.: writing – original draft, review and editing, conceptualization, methodology, resources, investigation, validation and formal analysis. S. T. R.: investigation. K. S.: conceptualization, review and editing, formal analysis, supervision. T. W.: conceptualization, writing, review and editing, formal analysis, supervision, funding acquisition and project administration.

## Conflicts of interest

The authors declare no conflict of interest.

## Supplementary Material

CB-006-D5CB00170F-s001

## Data Availability

The data supporting this article have been included as part of the supplementary information (SI). Supplementary information is available. The supporting information file contains detailed experimental procedures, compound synthesis routes, NMR and MS characterization data, full gel images, inhibition and fluorometric assay results, and fluorescence microscopy images. See DOI: https://doi.org/10.1039/d5cb00170f.
