# Supplementary material for: Selective labeling and visualization of viral and bacterial neuraminidases using ortho-quinone methide-based probes
Source: RSC Chem Biol. 2025 Oct 1;6(12):1909–19. doi: 10.1039/d5cb00170f (PMC12536644; doi:10.1039/d5cb00170f)

Supplementary Information (SI) for RSC Chemical Biology.
This journal is © The Royal Society of Chemistry 2025

Supporting Information

Selective Labeling and Visualization of Viral and Bacterial Neuraminidases using Ortho-Quinone Methide-Based Probes

Erianna I. Alvarado-Melendez, Simon T. Ruessink, Karin Strijbis & Tom Wennekes

Supporting Information:

1. **Supporting Information S1.** Synthesis and Characterization data (^{1}H-NMR, ^{13}C-NMR, ^{19}F-NMR, ESI-MS).
2. **Suporting Information S2.** Full lengths gel images.
3. **Supporting Information S3.** Inhibition of *Pt*NanH1 and *Pt*NanH2 using DANA (IC_{50}).
4. **Supporting Information S4.** Fluorometric assay for neuraminidase activity detection of recombinant *Pt*NanH1 and *Pt*NanH2 after labeling with **EA-229**.
5. **Supporting Information S5.** Gel analysis. Attempts to decrease off-labeling in *P. timonensis* 5C-B1 pellet using **EA-229**.
6. **Supporting Information S6.** Fluorometric assay for neuraminidase activity detection of *P. timonensis* 5C-B1 pellet after incubation with **EA-229**.
7. **Supporting Information S7.** Fluorescence microscopy images of *P. timonensis* CRIS 5C-B1 fluorescently labeled using **EA-227** followed by CuAAC reaction with alkyne-AF_{488} (GFP and light channel).
8. **Supporting Information S8.** 4-MUNANA assay for the evaluation of the enzymatic activity of PtNanH1 and PtNanH2 in different buffers.

1. **Supporting Information S1.** Synthesis and characterization data (^{1}H-NMR, ^{13}C-NMR, ^{19}F-NMR, ESI-MS).

Synthesis and characterization:

All reagents were purchased from Merck, Thermo Fisher Scientific and Byosynth and were used without further purification. Organic solvents were dried for 24 h over pre-activated (2 h, 400 °C) 4Å molecular sieves prior to use. Glassware for anhydrous reactions was flame-dried and cooled under a nitrogen atmosphere. Thin layer chromatography (TLC) was performed on aluminium-backed SiliaPlate TLC Plates F254 (Silicycle, Canada) and detected by UV (254 nm or 365 nm) where applicable and by dipping in 10% sulfuric acid in ethanol, *p*-anisaldehyde sugar stain or ceric ammonium molybdate stain followed by heating. Analytical thin layer chromatography (TLC) was performed on glass-backed TLC plates pre-coated with silica gel (60G, F_{254}). Column chromatography was carried out using silica gel (40-63 µm; VWR chemicals) or C18-reversed phase silica gel (40-63 µm; Merck). Solvents were evaporated at maximum 40 °C under reduced pressure.

Nuclear magnetic resonance (NMR) spectra were recorded on a 400 MHz Agilent spectrometer (400 and 101 MHz) or a 600 MHz Bruker Avance Neo spectrometer (600 and 125 MHz). Chemical shifts are reported in parts per million (ppm) relative to residual solvent peak. Mass spectra were recorded by ESI on a Bruker micrOTOF-QII mass spectrometer.

Methyl 4,7,8,9-tetra-*O*-acetyl-N-acetyl-2-chloro-2-deoxy-D-neuraminate (2):

Protocol adapted from reference. (H. Hinou, M. Kurogochi, H. Shimizu and S. I. Nishimura, *Biochemistry*, 2005, **44**, 11669–11675. H. Kai, H. Hinou, K. Naruchi, T. Matsushita and S. I. Nishimura, *Chem. - A Eur. J.*, 2013, **19**, 1364–1372).

Methyl (2-*O*-(2-formyl-4-nitro)phenyl-5-acetamido-4,7,8,9-tetra-*O*-acetyl-3,5-dideoxy-D-glycero-α-D-galacto-2-nonulopyranosid)onate (3):

2-hydroxy-5-nitrobenzaldehyde (690 mg, 4.13 mmol) was dissolved in 8 mL of dry CH_3CN under N_2 atmosphere, then 2 mL of DIPEA was added and the reaction mixture was left to stir at room temperature for 15 min. Compound **2** (1.92 g, 3.77 mmol) was added to the mixture and the reaction was left to stir for 18 h under nitrogen at room temperature. The solvent was evaporated under reduced pressure, and the residue was resuspended in EtOAc (150 mL). The organic layer was washed three times with (150 mL) 5% citric acid (aq.), and one time with (150 mL) an aqueous solution 5% $NaHCO_3$. The organic layer was dried over Na_2SO_4 and evaporated. The product was loaded onto a silica gel column and eluted with 20% acetone in PE to give compound **3** (2.21 g, 3.45 mmol, 92%) as a yellow syrup. R_f = 0.5 (PE/methanol/toluene, 4:2:2). ^{1}H NMR (400 MHz, $CDCl_3$) δ 10.40 (d, *J* = 0.5 Hz, 1H, CHO), 8.68 (d, *J* = 2.9 Hz, 1H, aromatic), 8.41 (ddd, *J* = 9.3, 2.9, 0.5 Hz, 1H, aromatic), 7.42 (d, *J* = 9.2 Hz, 1H, aromatic), 5.37 (ddd, *J* = 12.0, 7.1, 2.6 Hz, 1H, H7), 5.33 (dd, *J* = 9.4, 1.7 Hz, 1H, H8), 5.27 (d, *J* = 9.9 Hz, 1H), 5.03 (ddd, *J* = 11.9, 10.2, 4.6 Hz, 1H, H4), 4.64 (dd, *J* = 10.8, 1.6 Hz, 1H, H9), 4.21 (dd, *J* = 12.5, 2.4 Hz, 1H, H6), 4.14 (t, *J* = 10.3 Hz, 1H, H9'), 4.06 (dd, *J* = 12.4, 5.0 Hz, 1H, H5), 3.67 (s, 3H, OCH_3), 2.82 (dd, *J* = 13.1, 4.7 Hz, 1H, H-3e), 2.38 (dd, *J* = 13.2, 12.0 Hz, 1H, H-3a), 2.18 (s, 3H, OAc), 2.11 (s, 3H, OAc), 2.06 (s, 3H, OAc), 2.04 (s, 3H, OAc), 1.93 (s, 3H, NAc). ^{13}C NMR (101 MHz, $CDCl_3$) δ 187.01, 170.80, 170.54, 170.25, 170.09, 170.00, 167.59, 159.99, 143.63, 130.51, 126.28, 124.19, 119.34, 100.02, 73.86, 67.92, 67.82, 66.83, 62.16, 53.63, 49.41, 38.61, 30.90, 23.20, 20.97, 20.78, 20.70. HRMS (ESI): *m/z* calcd for $C_{27}H_{32}N_2O_{16}N+Na^+$ 663.1649; found: 663.1636. The ^{1}H and ^{13}C spectra of this compound were identical to previously reported spectra. (H. Hinou, M. Kurogochi, H. Shimizu and S. I. Nishimura, *Biochemistry*, 2005, **44**, 11669–11675. H. Kai, H. Hinou, K. Naruchi, T. Matsushita and S. I. Nishimura, *Chem. - A Eur. J.*, 2013, **19**, 1364–1372).

Methyl (2-*O*-(2-hydroxymethyl-4-nitro)phenyl-5-acetamido-4,7,8,9-tetra-*O*-acetyl-3,5-dideoxy-D-glycero-α-D-galacto-2-nonulopyranosid)onate (4):

Compound **3** (1.0 g, 1.56 mmol) and $NaB(OAc)_3H$ (3.3 g, 15.57 mmol) were dissolved in 200 mL of dry EtOH at 0°C under N_2 atmosphere. The reaction was stirred for 18 hours and allowed to reach room temperature. The mixture was diluted with 700 mL EtOAc, and washed 5 times with 100 mL of a solution 5% $NaHCO_3$, and two times with 100 mL of brine solution. The organic layer was dried over Na_2SO_4, filtered and evaporated under reduced pressure. The residue, crude product **4** (0.89 g, 1.39 mmol, ~89% crude yield) was used for the next reaction without further purification. R_f = 0.3 (PE/methanol/toluene, 7:2:1). ^{1}H NMR (400 MHz, $CDCl_3$) δ 8.31 (d, *J* = 2.9 Hz, 1H, aromatic), 8.13 (dd, *J* = 9.1, 2.9 Hz, 1H, aromatic), 7.25 (d, *J* = 9.1 Hz, 1H, aromatic), 5.41 – 5.25 (m, 2H, H7, H8), 5.00 (ddd, *J* = 11.3, 10.1, 4.8 Hz, 1H, H4), 4.74 (d, *J* = 7.2 Hz, 2H, CH_2), 4.58 (dd, *J* = 10.9, 1.6 Hz, 1H, H9), 4.23 (dd, *J* = 12.5, 2.4 Hz, 1H, H6), 4.14 – 3.99 (m, 2H, H5, H9), 3.65 (d, *J* = 0.5 Hz, 3H, OCH_3), 2.74 (dd, *J* = 13.2, 4.8 Hz, 1H, H-3eq), 2.31 (dd, *J* = 13.2, 11.4 Hz, 1H, H-3ax), 2.16 (d, *J* = 3.0 Hz, 3H, OAc), 2.09 – 2.06 (m, 3H, OAc), 2.05 – 2.04 (m, 3H, OAc), 1.91 (s, 3H, OAc). ^{13}C NMR (101 MHz, $CDCl_3$) δ 170.79, 170.61, 170.31, 170.02, 169.98, 167.79, 156.05, 143.58, 132.68, 124.62, 123.74, 117.42, 99.83, 77.30, 76.98, 76.67, 73.64, 68.37, 68.11, 67.05, 62.16, 60.00, 58.44, 53.49, 49.32, 38.47, 23.19, 20.95, 20.78, 20.70, 20.66, 18.40.

Methyl (2-*O*-(2-difluoromethyl-4-nitro)phenyl-5-acetamido-4,7,8,9-tetra-*O*-acetyl-3,5-dideoxy-D-glycero-α-D-galacto-2-nonulopyranosid)onate (6):

Compound **3** (1.0 g, 1.56 mmol) was dissolved in 20 mL dry DCM at 0°C under N_2 atmosphere. DAST (1.24 mL, 9.39 mmol) was added dropwise and the resulting reaction mixture was stirred at 0°C for 4h. It was subsequently quenched by addition of 1 mL of CH_3OH and silica. The mixture was washed twice with 20 mL of an ice cold 1% $NaHCO_3$ (aq.)solution, and twice with 20 mL of ice cold water. The organic layer was dried over Na_2SO_4, filtered and evaporated under reduced pressure. The residue was purified via silica gel column chromatography (petroleum ether/EtOAc 1:1 to 100% EtOAc). Compound **6** (0.449 g, 0.951 mmol) was obtained with a yield of 61%. R_f = 0.4 (PE/methanol/toluene, 7:2:1) as a yellow syrup. ^{1}H NMR (400 MHz, $CDCl_3$) δ 8.45 (d, *J* = 2.9 Hz, 1H, aromatic), 8.32 (ddd, *J* = 9.2, 2.3, 1.5 Hz, 1H, aromatic), 7.40 (dt, *J* = 9.2, 1.2 Hz, 1H, aromatic), 6.88 (d, *J* = 54.8 Hz, 1H, CHF), 5.40 – 5.29 (m, 2H, H7, H8), 5.24 (d, *J* = 9.9 Hz, 1H), 5.01 (ddd, *J* = 11.8, 10.2, 4.7 Hz, 1H, H4), 4.62 (dd, *J* = 10.9, 1.7 Hz, 1H, H9), 4.22 (dd, *J* = 12.5, 2.6 Hz, 1H, H6), 4.15 – 4.02 (m, 2H, H9', H5), 3.64 (s, 3H, OCH_3), 2.77 (dd, *J* = 13.2, 4.7 Hz, 1H, H-3eq), 2.32 (dd, *J* = 13.2, 11.8 Hz, 1H, H-3ax), 2.18 (s, 3H, OAc), 2.10 (s, 3H, OAc), 2.05 (s, 3H, OAc), 2.04 (s, 3H, OAc), 1.92 (s, 3H, OAc). ^{13}C NMR (101 MHz, $CDCl_3$) δ 170.82, 170.24, 170.06, 167.73, 127.85, 117.41, 99.81, 79.89, 78.20, 77.30, 77.18, 76.98, 76.97, 76.66, 73.71, 68.17, 68.01, 66.94, 62.11, 53.46, 49.36, 38.61, 23.21, 20.98, 20.78, 20.70.^{19}F NMR (376 MHz, $CDCl_3$) δ -221.28 (t, *J* = 47.0 Hz). HRMS (ESI): *m/z* calcd for $C_{27}H_{32}F_2N_2O_{15}+H^+$ 663.1871; found: 663.1849.

Methyl (2-*O*-(2-fluoromethyl-4-nitro)phenyl-5-acetamido-4,7,8,9-tetra-*O*-acetyl-3,5-dideoxy-D-glycero-α-D-galacto-2-nonulopyranosid)onate (5):

Compound **4** (820 mg, 1.28 mmol) was dissolved in 15 mL dry DCM at 0°C under N_2 atmosphere. DAST (0.52 mL, 3.94 mmol) was added dropwise and the resulting reaction mixture was stirred at 0°C for 2h . It was subsequently quenched by addition of 1 mL CH_3OH and silica. The mixture was washed three times with 20 mL of a 1% $NaHCO_3$ ice cold solution, and twice with 20 mL of ice cold water. The organic layer was dried over Na_2SO_4, filtered and evaporated under reduced pressure. The residue was purified via silica gel column chromatography (PE/methanol/toluene, 7:2:1). Compound **5** (0.630 g, 0.977 mmol) was obtained with a 77% yield as a yellow syrup. R_f = 0.3 (DCM/EtOAc, 3:7). ^{1}H NMR (400 MHz, $CDCl_3$) δ 8.28 (dd, *J* = 2.9, 0.9 Hz, 1H, aromatic), 8.20 (dd, *J* = 9.0, 2.9 Hz, 1H, aromatic), 7.30 (dd, *J* = 9.2, 1.3 Hz, 1H, aromatic), 5.46 (d, *J* = 47.0 Hz, 2H, CH_2F), 5.38 – 5.29 (m, 2H, H7, H8), 5.24 (d, *J* = 9.9 Hz, 1H), 4.98 (ddd, *J* = 11.9, 10.2, 4.7 Hz, 1H, H4), 4.61 (dd, *J* = 10.9, 1.7 Hz, 1H, H9), 4.22 (dd, *J* = 12.5, 2.5 Hz, 1H, H6), 4.14 – 3.98 (m, 2H, H5, H9'), 3.63 (s, 3H, OCH_3), 2.74 (dd, *J* = 13.1, 4.7 Hz, 1H, 3H-eq), 2.30 (dd, *J* = 13.2, 12.0 Hz, 1H, 3H-ax), 2.18 (s, 3H, OAc), 2.10 (s, 3H, OAc), 2.05 (s, 3H, OAc), 2.04 (s, 3H, OAc), 1.92 (s, 3H, OAc).^{13}C NMR (101 MHz, $CDCl_3$) δ 170.80, 170.24, 170.09, 170.00, 167.50, 118.36, 110.04, 100.05, 77.30, 77.18, 76.98, 76.67, 73.72, 67.98, 67.89, 66.86, 62.13, 53.54, 49.41, 38.54, 23.21, 20.98, 20.76. ^{19}F NMR (376 MHz, $CDCl_3$) δ -116.42 (dd, *J* = 54.8, 7.2 Hz). HRMS (ESI): *m/z* calcd for $C_{27}H_{33}FN_2O_{15}+H^+$ 645.1943; found: 645.1915.

Methyl (2-*O*-(2-fluoromethyl-4-amino)phenyl-5-acetamido-4,7,8,9-tetra-*O*-acetyl-3,5-dideoxy-D-glycero-α-D-galacto-2-nonulopyranosid)onate (7):

Compound **5** (500 mg, 0.755 mmol) was dissolved in 100 mL dry EtOAc at RT under N_2 atmosphere. Next, 94 mg of Pd/C (10% weight) was added, and H_2 (g) was bubbled through the solution for 5 minutes. The reaction was left to stir under a H_2 atmosphere at RT for 6 h. The suspension was filtered over celite, the flow-through was diluted with 20 mL of EtOAc and washed three times with 50 mL ice cold H_2O, and one time with 50 mL brine. The organic layer was dried over Na_2SO_4, filtered and evaporated under reduced pressure. Crude product **7** was isolated with a ~83% crude yield (0.398 g, 0.629 mmol), and was used for the next reaction without further purification. R_f = 0.25 (DCM/EtOAc, 2:8). ^{1}H NMR (400 MHz, $CDCl_3$) δ 7.02 (dd, *J* = 8.7, 1.3 Hz, 1H, aromatic), 6.68 (dd, *J* = 2.9, 0.8 Hz, 1H, aromatic), 6.56 (ddd, *J* = 8.7, 2.9, 1.2 Hz, 1H, aromatic), 5.35 – 5.31 (m, 2H, H7, H8), 5.28 – 5.40 (dd, *J* = 47.9, 2.7 Hz, CHF), 5.19 (d, *J* = 10.0 Hz, 1H), 4.91 (ddd, *J* = 12.2, 10.3, 4.6 Hz, 1H, H4), 4.29 (ddd, *J* = 10.8, 7.1, 1.3 Hz, 2H, H9), 4.21 – 3.94 (m, 3H, H9', H5, H6), 3.63 (s, 3H, OCH_3), 2.66 (dd, *J* = 12.9, 4.6 Hz, 1H, 3H-eq), 2.14 (m, 1H, 3H-ax), 2.13 (s, 3H, OAc), 2.11 (s, 3H, OAc), 2.04 (s, 3H, OAc), 2.01 (s, 3H, OAc), 1.89 (s, 3H, OAc). ^{13}C NMR (101 MHz, $CDCl_3$) δ 170.91, 170.60, 170.20, 170.00, 169.97, 167.58, 143.18, 121.65, 115.74, 114.72, 100.83, 80.82, 77.30, 76.98, 76.66, 73.11, 69.09, 68.80, 67.25, 61.93, 52.90, 49.43, 37.60, 23.20, 20.98, 20.80, 20.75, 20.73. ^{19}F NMR (376 MHz, $CDCl_3$) δ -216.20 (t, *J* = 47.6 Hz).

Methyl (2-*O*-(2-difluoromethyl-4-amino)phenyl-5-acetamido-4,7,8,9-tetra-*O*-acetyl-3,5-dideoxy-D-glycero-α-D-galacto-2-nonulopyranosid)onate (8):

Compound **6** (550 mg, 0.853 mmol) was dissolved in 100 mL dry EtOAc at RT under N_2 atmosphere. Next, 94 mg of Pd/C (10% weight) was added, and H_2 (g) was bubbled through the solution for 5 minutes. The reaction was left to stir under a H_2 atmosphere at RT for 12h. The suspension was filtered over celite, the flow-through was diluted with 20 mL EtOAc and washed three times with 50 mL of ice cold H_2O, and 50 mL of brine solution. The organic layer was dried over Na_2SO_4, filtered and evaporated under reduced pressure. Crude product **8** (0.395 g, 0.643 mmol) was obtained with a ~75% crude yield, and it was used for the next reaction without further purification. R_f = 0.3 (DCM/EtOAc, 3:7). ^{1}H NMR (400 MHz, $CDCl_3$) δ 7.08 (dt, *J* = 8.8, 1.2 Hz, 1H, aromatic), 6.83 (d, *J* = 3.1 Hz, 1H, aromatic), 6.72 – 6.62 (m, 1H, aromatic), 5.38 – 5.32 (m, 2H, H7, H8), 5.18 (d, *J* = 10.0 Hz, 1H), 4.92 (ddd, *J* = 12.1, 10.3, 4.6 Hz, 1H, H4), 4.36 – 4.26 (m, 2H, H6, H9), 4.19 – 3.97 (m, 2H, H9', H5), 3.63 (s, 3H, OCH_3), 2.68 (dd, *J* = 12.9, 4.6 Hz, 1H, 3H-eq), 2.23 – 2.14 (m, 1H, 3H-ax), 2.13 (s, 3H, OAc), 2.11 (s, 3H, OAc), 2.03 (s, 3H, OAc), 2.02 (s, 3H, OAc), 1.89 (s, 3H, OAc). ^{13}C NMR (101 MHz, $CDCl_3$) δ 171.14, 170.89, 170.20, 169.98, 167.32, 122.07, 77.30, 76.98, 76.66, 73.13, 68.82, 68.64, 67.11, 61.91, 60.37, 52.96, 49.45, 37.71, 23.20, 20.97, 20.79, 20.75, 20.71, 14.17. ^{19}F NMR (376 MHz, $CDCl_3$) δ -112.47 (dd, *J* = 302.7, 55.5 Hz), -115.21 (dd, *J* = 302.6, 55.4 Hz). HRMS (ESI): *m/z* calcd for $C_{27}H_{34}F_2N_2O_{13}$+Na^+ 655.1926; found: 655.1904.

2-Azidoacetyl chloride:

This compound was synthesized as reported previously. The ^{1}H and ^{13}C spectra were identical to previously reported spectra. (V. Haridas, Y. K. Sharma, S. Sahu, R. P. Verma, S. Sadanandan and B. G. Kacheshwar, *Tetrahedron*, 2011, **67**, 1873–1884).

Methyl (2-*O*-(2-difluoromethyl-4-(2-azidoacetamido)phenyl-5-acetamido-4,7,8,9-tetra-*O*-acetyl-3,5-dideoxy-D-glycero-α-D-galacto-2-nonulopyranosid)onate (10):

Compound **8** (302.0 mg, 0.853 mmol) was dissolved in 5 mL dry DCM at 0°C under N_2 atmosphere. Next, 2-azidoacetyl chloride (230 µL, 2.23 mmol) was added, followed by addition of pyridine until pH 8 was reached. The reaction mixture was left stirring for 18 h at RT and was subsequently quenched with the addition of 5 mL of CH_3OH. The resulting solution was diluted with 100 mL DCM, washed five times with 20 mL ice cold solution of citric acid 5%, and then five times with 20 mL of ice cold $NaHCO_3$ 1% solution. We found that this product is prone to hydrolysis, despite our attempts to prevent hydrolysis of the product during the washing steps and evaporation of the solvent, we still recovered a mixture of product and hydrolyzed product, as seen in the TLC and spectroscopic data (See Supporting information S1). We continue with the deprotection step using the crude mixture. R_f = 0.2 (PE/EtOAc, 6:4). ^{1}H NMR (400 MHz, CD_3OD) δ 7.85 (d, *J* = 2.7 Hz, 1H, aromatic), 7.60 (dd, *J* = 9.0,

2.7 Hz, 1H, aromatic), 7.28 (d, *J* = 9.1 Hz, 1H, aromatic), 7.06 (dd, *J* = 8.8, 1.3 Hz, 1H), 6.92 – 6.81 (m, 1H), 6.77 – 6.68 (m, 1H), 5.34 (q, *J* = 2.4 Hz, 3H), 4.49 (dd, *J* = 10.9, 1.4 Hz, 1H, H9), 4.36 (dd, *J* = 10.8, 1.5 Hz, 1H), 4.31 (dd, *J* = 12.4, 2.2 Hz, 1H), 3.64 (s, 3H), 2.80 (dd, *J* = 13.0, 4.8 Hz, 1H), 2.75 (dd, *J* = 12.8, 4.7 Hz, 1H), 2.08 (s, 4H), 1.99 (s, 7H), 1.84 (s, 3H). ^{19}F NMR (376 MHz, CD_3OD) δ -113.30 (dd, *J* = 304.0, 55.6 Hz, product), -114.77 (dd, *J* = 303.8, 55.2 Hz), -116.38 (dd, *J* = 303.7, 55.4 Hz, product), -116.63 (dd, *J* = 303.2, 55.3 Hz). HRMS (ESI): *m/z* calcd for $C_{29}H_{36}FN_5O_{14}$+Na^+ 720.2140; found: 720.1880.

Methyl (2-*O*-(2-fluoromethyl-4-(2-azidoacetamido)phenyl-5-acetamido-4,7,8,9-tetra-*O*-acetyl-3,5-dideoxy-D-glycero-α-D-galacto-2-nonulopyranosid)onate (9):

Compound **7** (350.0 mg, 0.553 mmol) was dissolved in 5 mL dry DCM at 0°C under N_2 atmosphere. Next, 2-azidoacetyl chloride (282 µL, 2.60 mmol) was added, followed by addition of pyridine until pH 8 was reached. The reaction mixture was left stirring for 18 h at RT and was subsequently quenched with the addition of 5 mL of CH_3OH. The resulting solution was diluted with 100 mL DCM, washed five times with 20 mL of ice cold solution of citric acid 5%, and then five times with 20 mL of ice cold $NaHCO_3$ 1% solution. The product is prone to hydrolysis, despite our attempts to prevent hydrolysis of the product during the washing steps and evaporation of the solvent, we still recovered a mixture of product and hydrolyzed product, as seen in the TLC and spectroscopic data (See Supporting information S1). We continue with the deprotection step using the crude mixture. R_f = 0.4 (DCM/EtOAc, 9:1). ^{1}H NMR (400 MHz, CD_3OD) δ 7.68 – 7.63 (m, 1H), 7.50 – 7.41 (m, 1H), 7.20 (dd, *J* = 9.0, 1.2 Hz, 2H), 7.18 – 7.08 (m, 1H), 7.01 (dd, *J* = 8.7, 1.3 Hz, 1H), 6.74 (d, *J* = 2.9 Hz, 1H), 6.61 (ddd, *J* = 8.7, 2.8, 1.4 Hz, 1H), 5.51 – 5.45 (m, 1H), 5.40 – 5.29 (m, 6H), 4.82 (s, 15H), 4.46 (dd, *J* = 10.8, 1.5 Hz, 1H), 4.37 – 4.24 (m, 3H), 3.64 (s, 3H), 3.63 (s, 3H), 2.78 (dd, *J* = 13.0, 4.7 Hz, 1H), 2.73 (dd, *J* = 12.8, 4.7 Hz, 1H). ^{19}F NMR (376 MHz, cd_3od) δ -215.83 (t, *J* = 47.9 Hz), -217.72 (t, *J* = 47.7 Hz). HRMS (ESI): *m/z* calcd for $C_{29}H_{35}F_2N_5O_{14}$+H^+ 716.2226; found: 716.2186. $C_{29}H_{35}F_2N_5O_{14}$+Na^+ 738.2046; found: 738.2008.

Methyl (2-*O*-(2-difluoromethyl-4-(2-azidoacetamido)phenyl-5-acetamido-3,5-dideoxy-D-glycero-α-D-galacto-2-nonulopyranosid)onate (EA-226):

Crude 10 (54 mg, 75.46 µmol) was dissolved in dry CH_3OH, under argon atmosphere and Na_2CO_3 (26 mg, 246 µmol) was added. The reaction mixture was stirred at RT for 3 h. Next, the solvent was evaporated under reduced pressure at 35°C. The residue was redissolved in an aqueous solution of NaOH 0.1 M (pH 11), and the mixture was stirred for 18 h at RT. The resulting solution was freeze dried, resuspended in H_2O, and loaded onto a C18 silica gel reverse phase column (gradient: 0% → 30% CH_3CN in H_2O), where the product was separated from the hydrolyzed compound with a yield of 10% over two steps (4.2 mg, 7.56 µmol). ^{1}H NMR (600 MHz, D_2O) δ 7.63 (d, *J* = 2.6 Hz, 1H, aromatic), 7.42 (dd, *J* = 8.9, 2.7 Hz, 1H, aromatic), 7.31 (d, *J* = 8.9 Hz, 1H, aromatic), 7.04 (d, *J* = 55.9 Hz, 1H, CHF_2), 3.89 – 3.82 (m, 1H), 3.81 (dd, *J* = 10.4, 1.6 Hz, 1H, H9), 3.80 – 3.74 (m, 2H), 3.70 (ddd, *J* = 11.6, 9.3, 4.7 Hz, 1H), 3.59 – 3.50 (m, 2H), 2.84 (dd, *J* = 12.6, 4.7 Hz, 1H, 3H-eq), 1.97 (d, *J* = 0.8 Hz, 3H, OAc), 1.90 (t, *J* = 12.2 Hz, 1H, 3H-ax). ^{13}C NMR (151 MHz, D_2O) δ 175.08, 172.13, 169.18, 125.64, 121.89, 103.09, 73.54, 71.68, 68.15, 68.02, 62.63, 52.12, 51.66, 40.42, 22.01.^{19}F NMR (376 MHz, D_2O) δ -111.20 (dd, *J* = 301.0, 55.4 Hz), -116.36 (dd, *J* = 301.0, 54.7 Hz). R_f = 0.4 (EtOAc/MeOH/H_2O, 16:3:1). HRMS (ESI): *m/z* calcd for $C_{20}H_{25}F_2N_5O_{10}$+Na^+ 556,1467; found: 556,1435.

Methyl (2-*O*-(2-difluoromethyl-4-(2-azidoacetamido)phenyl-5-acetamido-3,5-dideoxy-D-glycero-α-D-galacto-2-nonulopyranosid)onate (EA-227):

Crude 9 (54 mg, 77 µmol) in dry CH_3OH, under argon atmosphere and Na_2CO_3 (28 mg, 271 µmol) was added. The reaction was stirred at RT for 3 h. Next, the solvent was evaporated under reduced pressure at 35°C. The residue was redissolved in an aqueous solution of NaOH 0.1 M (pH 11), and the mixture was stirred for 18 h at RT. The resulting solution was freeze dried, the residue was resuspended in H_2O, and loaded onto a C18 silica gel reverse phase column (gradient: 0% → 30% CH_3CN in H_2O) where the product was partially separated from the hydrolyzed compound with a yield of 12% over two steps (5.0 mg, 9.30 µmol). ^{1}H NMR (400 MHz, D_2O) δ 7.39 (s, 1H, aromatic), 7.22 (t, *J* = 7.3 Hz, 2H, aromatic), 5.65 – 5.20 (m, 2H, CH_2F), 4.04 (s, 2H, CH_2), 3.73 (t, *J* = 10.6 Hz, 4H), 3.55 – 3.36 (m, 3H, OCH_3), 2.77 (dd, *J* = 12.6, 4.7 Hz, 1H, 3H-ax), 1.90 (s, OAc), 1.82 (t, *J* = 12.0 Hz, 1H, 3H-eq). HRMS (ESI): *m/z* calcd for $C_{20}H_{26}FN_5O_{10}$+Na^+ 538,1561; found: 538,1570. ^{19}F NMR (376 MHz, D_2O) δ -209.76 (t, *J* = 47.1 Hz), -210.12 (t, *J* = 47.6 Hz) hydrolyzed product. R_f = 0.2 (EtOAc/MeOH/H_2O, 16:3:1).

Methyl (2-*O*-(2-difluoromethyl-4-(2-PEG$_4$-biotin-acetamido)phenyl-5-acetamido-3,5-dideoxy-D-glycero-α-D-galacto-2-nonulopyranosid)onate (EA-228):

EA-226 (1.2 mg, 2.16 µmol) was dissolved in 300 µL degassed H_2O. Next, a solution of alkyne-PEG$_4$-biotin (2.4 mg, 5.40 µmol) in 35 µL (THF:H_2O, 1:1) was added to the mixture, followed by the addition of $CuSO_4$ with 3 equivalents of THPTA ligand (final concentration 0.3 mM), and a freshly prepared solution of Na-*L*-ascorbate (final concentration of 0.7 mM). The reaction was stirred at RT in the dark for 16 h. The reaction crude t was purified by C18 reverse phase silica column (H_2O→30% CH_3CN) and obtained as a white powder with 82% yield (1.8 mg, 1.78 µmol). ^{1}H NMR (600 MHz, D_2O) δ 7.98 (d, *J* = 1.3 Hz, 1H), 7.65 – 7.50 (m, 1H), 7.36 (dd, *J* = 8.9, 2.8 Hz, 1H), 7.23 (d, *J* = 9.0 Hz, 1H), 7.08 – 6.79 (m, 1H), 5.29 (s, 1H), 4.44 – 4.33 (m, 1H), 4.23 – 4.13 (m, 1H), 3.81 – 3.65 (m, 5H), 3.64 – 3.57 (m, 4H), 3.59 – 3.52 (m, 5H), 3.52 – 3.45 (m, 10H), 3.45 – 3.39 (m, 3H), 3.23 – 3.13 (m, 2H), 3.12 – 3.05 (m, 1H), 2.80 – 2.71 (m, 2H), 2.62 – 2.51 (m, 2H), 2.05 (dd, *J* = 8.0, 6.7 Hz, 2H), 1.88 (d, *J* = 1.3 Hz, 3H), 1.83 – 1.76 (m, 1H), 1.55 – 1.31 (m, 3H), 1.18 (dddd, *J* = 15.9, 8.8, 6.7, 2.2 Hz, 2H). ^{13}C NMR (151 MHz, D_2O) δ 176.83, 132.76, 121.99, 73.53, 71.68, 69.62, 69.55, 69.40, 68.96, 68.83, 63.06, 62.63, 62.01, 60.19, 55.28, 52.44, 51.67, 40.39, 39.65, 38.89, 35.41, 27.84, 27.63, 25.09, 22.01. ^{19}F NMR (376 MHz, D_2O) δ -111.15 (dd, *J* = 301.0, 55.3 Hz), -116.38 (dd, *J* = 301.0, 54.8 Hz). HRMS (ESI): *m/z* calcd for $C_{41}H_{60}F_2N_8O_{16}S+H^+$ 991,3894; found: 991,3820.

Methyl (2-*O*-(2-difluoromethyl-4-(2-PEG$_4$-biotin-acetamido)phenyl-5-acetamido-3,5-dideoxy-D-glycero-α-D-galacto-2-nonulopyranosid)onate (EA-229):

EA-227 (2.5 mg, 4.65 µmol) was dissolved in 300 µL of degassed H_2O. Then, a solution of alkyne-PEG$_4$-biotin (3.1 mg, 6.77 µmol) in 55 µL (THF:H_2O) was added to the mixture, followed by the addition of $CuSO_4$ with 3 equivalents of THPTA ligand (final concentration 0.3 mM), and a freshly prepared solution of Na-*L*-ascorbate (final concentration of 0.7 mM). The reaction was stirred at RT in the dark for 16 h. The product was purified by C18 reverse phase silica column (H_2O → 30% CH_3CN) and obtained as a white powder with 78% yield (3.6 mg, 3.62 µmol). ^{1}H NMR (600 MHz, D_2O) δ 7.55 – 7.50 (m, 1H), 7.34 (ddd, *J* = 8.9, 2.8, 1.5 Hz, 1H), 7.27 (dd, *J* = 8.9, 1.4 Hz, 1H), 5.66 – 5.40 (m, 1H), 5.37 (d, *J* = 1.3 Hz, 2H), 4.46 (dd, *J* = 7.9, 5.0 Hz, 1H), 4.30 – 4.24 (m, 1H), 3.86 – 3.75 (m, 5H), 3.72 – 3.66 (m, 4H), 3.66 – 3.61 (m, 4H), 3.61 – 3.55 (m, 9H), 3.53 – 3.48 (m, 3H), 3.27 (t, *J* = 5.1 Hz, 2H), 3.17 (dt, *J* = 9.9, 5.2 Hz, 1H), 2.84 (dddd, *J* = 14.0, 12.5, 4.8, 1.3 Hz, 2H), 2.69 – 2.60 (m, 2H), 2.16 – 2.10 (m, 2H), 1.97 (d, *J* = 1.4 Hz, 3H), 1.90 – 1.84 (m, 1H), 1.63 – 1.40 (m, 4H), 1.31 – 1.24 (m, 2H). ^{13}C NMR (151 MHz, D_2O) δ 176.83, 175.07, 165.96, 165.27, 126.48, 122.45, 121.73, 81.27, 80.22, 73.42, 71.79, 69.63, 69.61, 69.56, 69.54, 69.40, 68.97, 68.83, 68.14, 68.10, 63.06, 62.61, 62.01, 60.19, 55.29, 52.46, 51.70, 40.40, 39.65, 38.89, 35.42, 27.84, 27.63, 25.10, 22.01. ^{19}F NMR (376 MHz, D_2O) δ -210.02 (t, *J* = 47.3 Hz). HRMS (ESI): *m/z* calcd for $C_{41}H_{61}FN_8O_{16}S+H^+$ 973,3928; found: 973,3989.

diF-Neu9AF$_{647}$:

diF-Neu9Az was conjugated to alkyne-PEG$_4$-AF$_{647}$ as using the CuAAC reaction protocol described Experimental section of in this article. The crude mixture was used without further purification.

Spectroscopic data (^{1}H-NMR, ^{13}C-NMR, ^{19}F-NMR, ESI-MS):

Methyl (2-*O*-(2-formyl-4-nitro)phenyl-5-acetamido-4,7,8,9-tetra-*O*-acetyl-3,5-dideoxy-D-glycero-α-D-galacto-2-nonulopyranosid)onate (3):

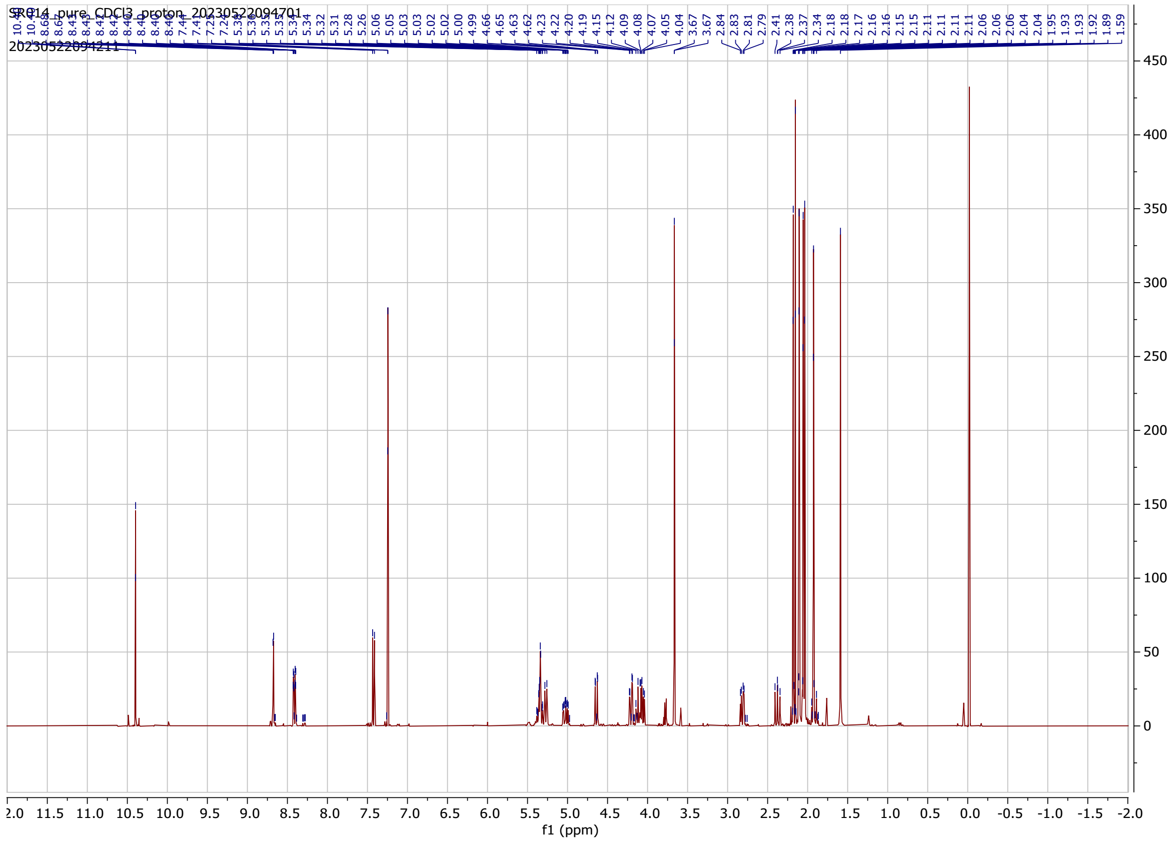

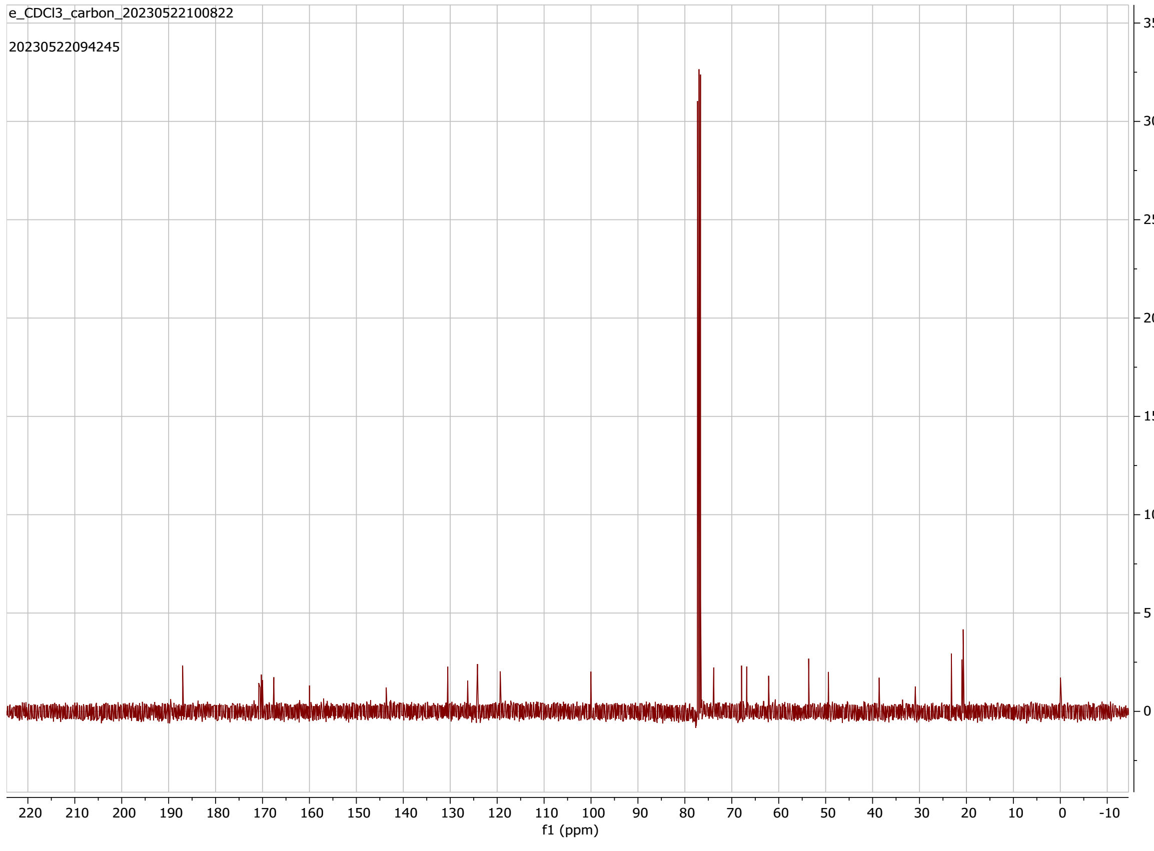

Methyl (2-*O*-(2-hydroxymethyl-4-nitro)phenyl-5-acetamido-4,7,8,9-tetra-*O*-acetyl-3,5-dideoxy-D-glycero-α-D-galacto-2-nonulopyranosid)onate (4):

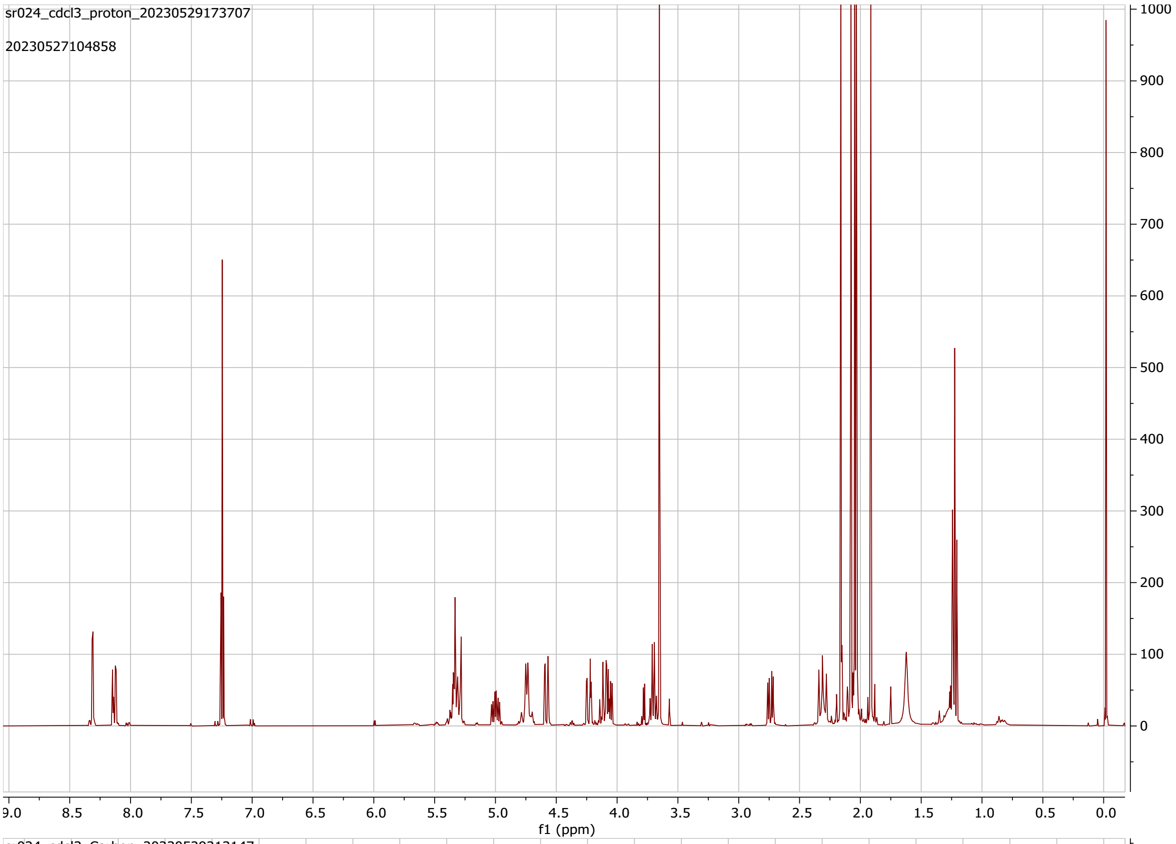

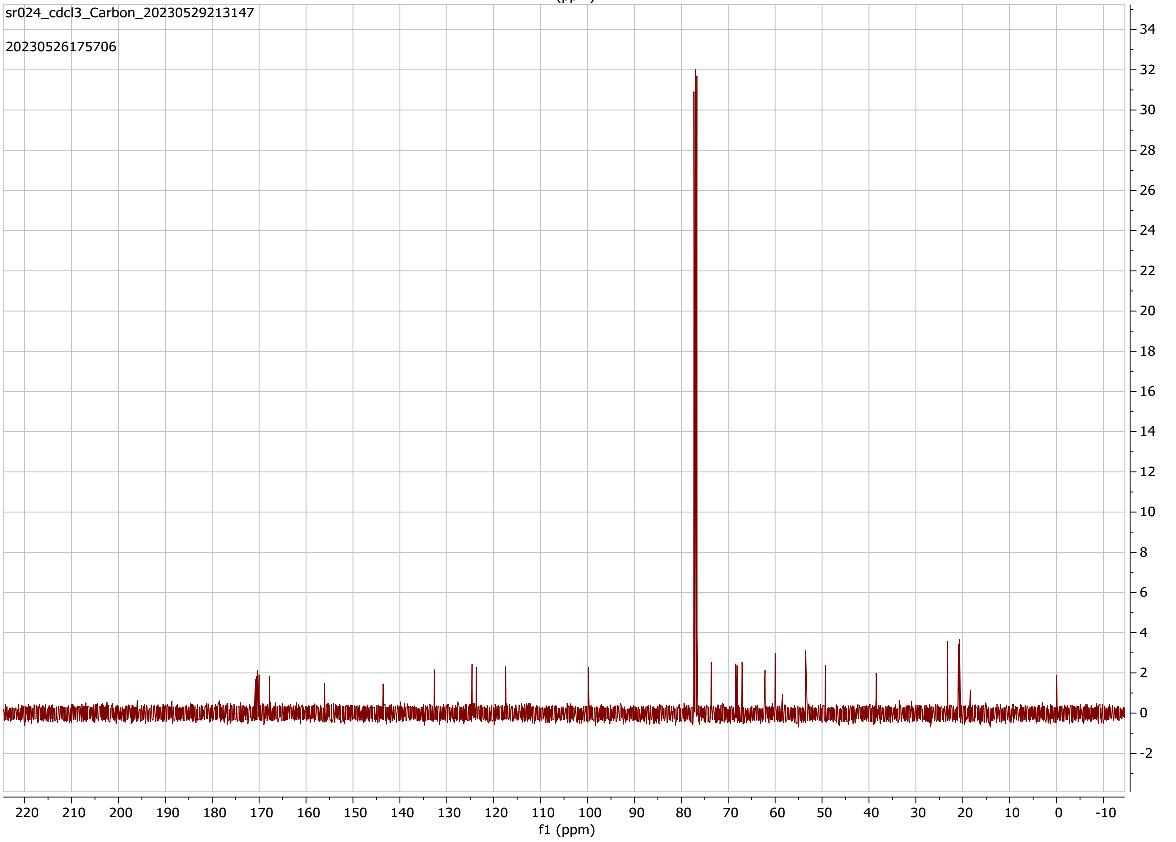

Methyl (2-*O*-(2-difluoromethyl-4-nitro)phenyl-5-acetamido-4,7,8,9-tetra-*O*-acetyl-3,5-dideoxy-D-glycero-α-D-galacto-2-nonulopyranosid)onate (6):

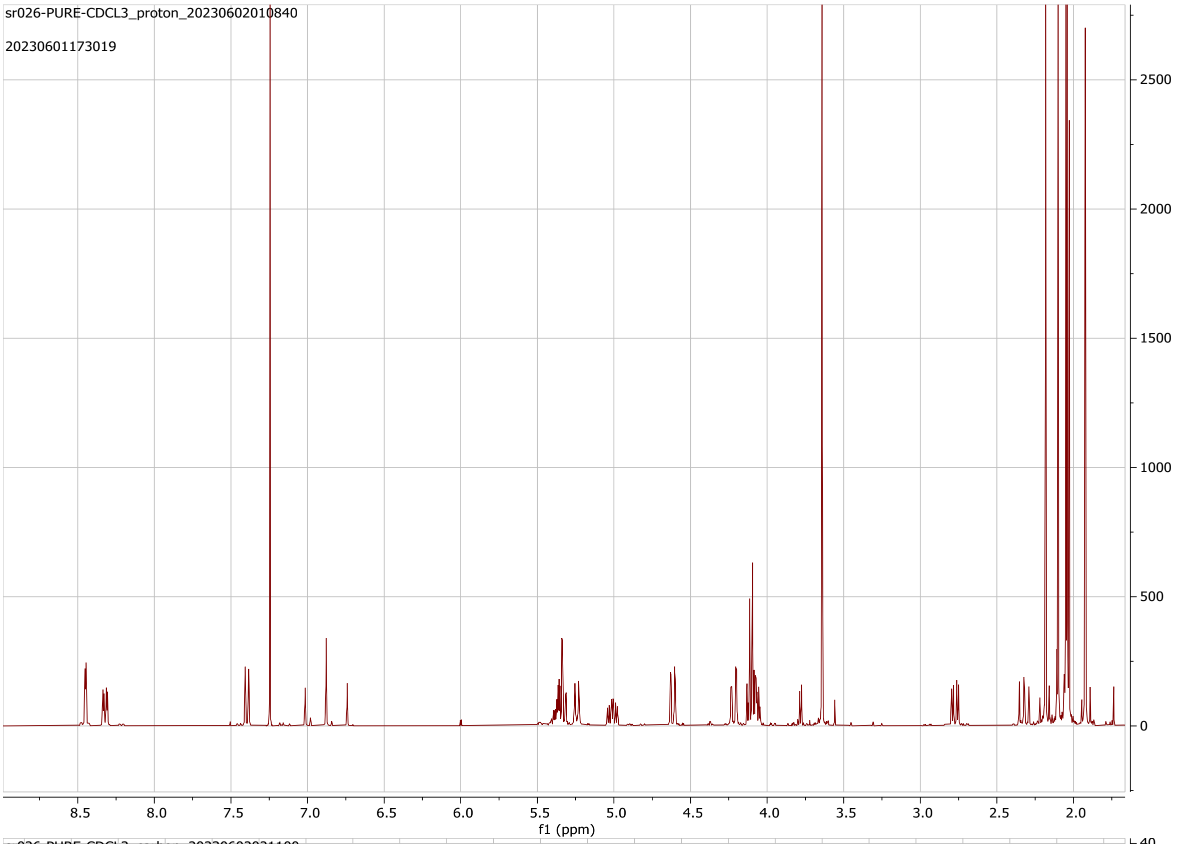

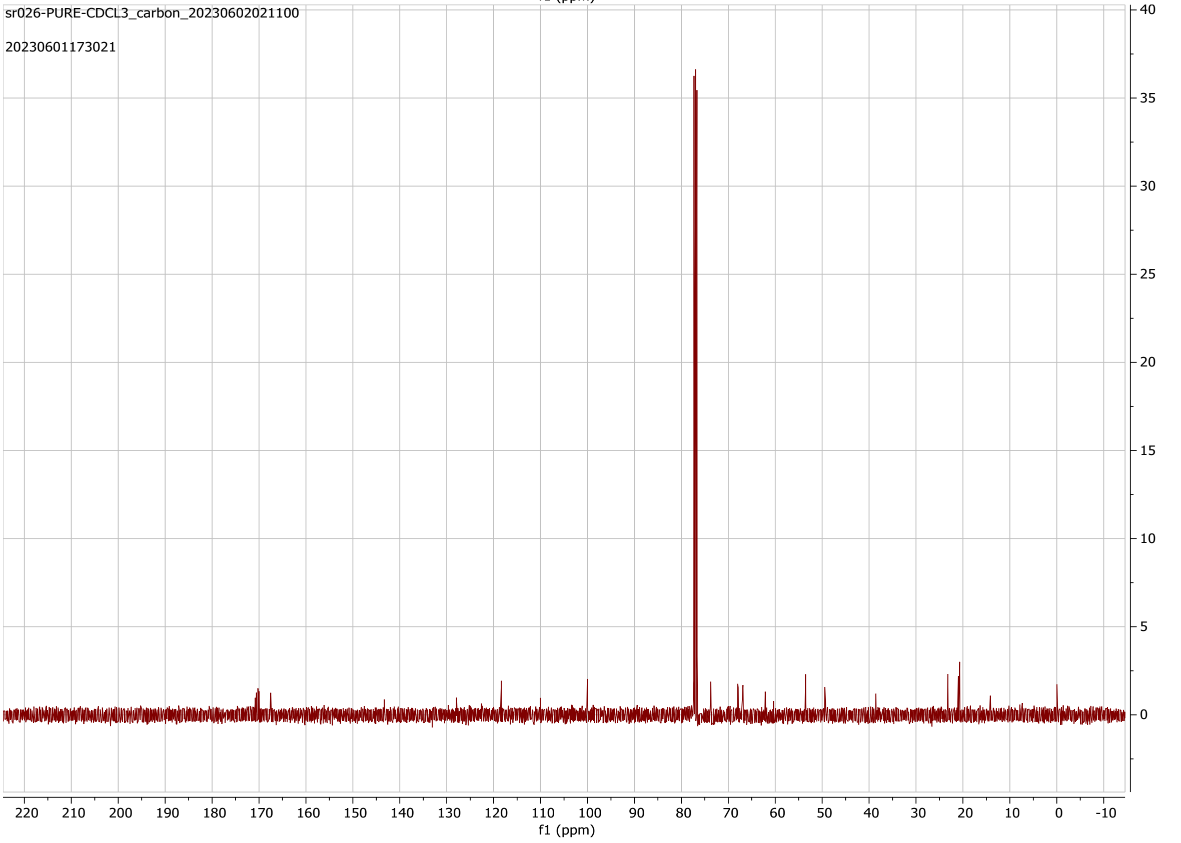

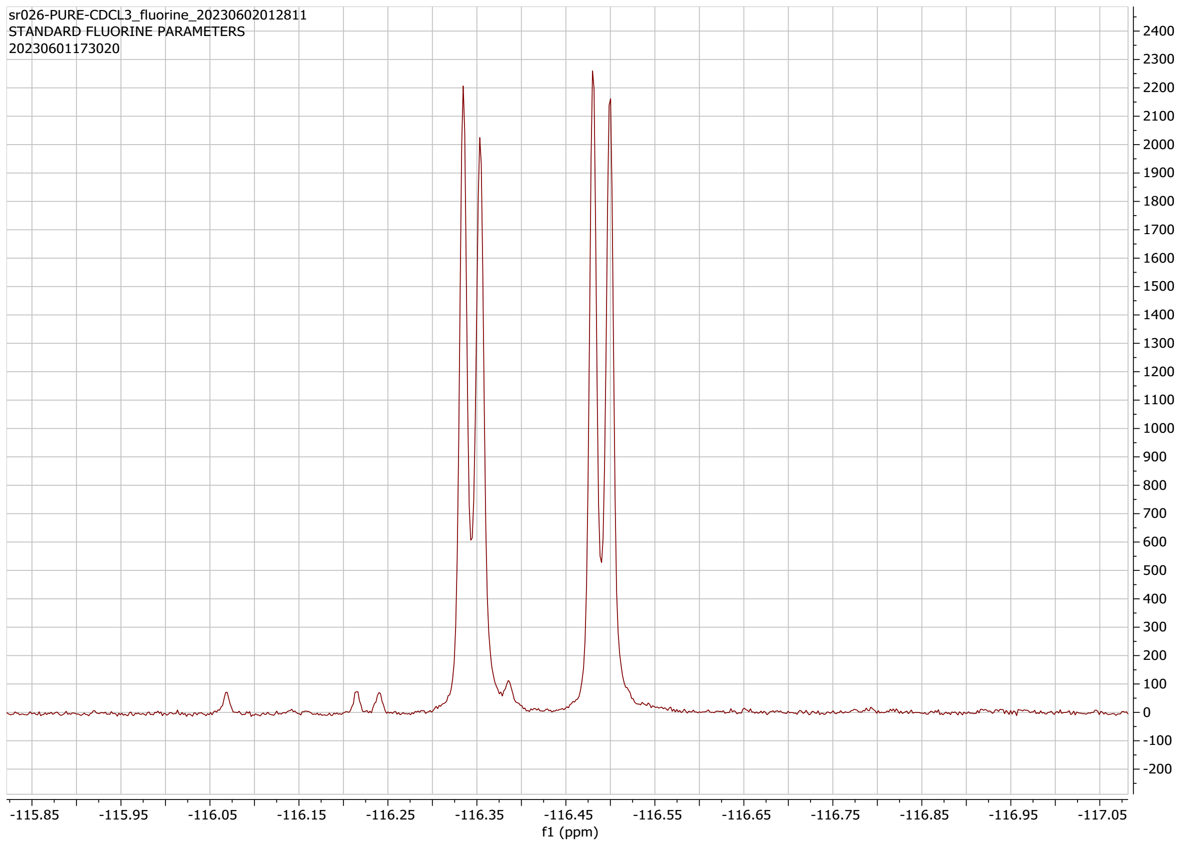

Methyl (2-*O*-(2-fluoromethyl-4-nitro)phenyl-5-acetamido-4,7,8,9-tetra-*O*-acetyl-3,5-dideoxy-D-glycero-α-D-galacto-2-nonulopyranosid)onate (5):

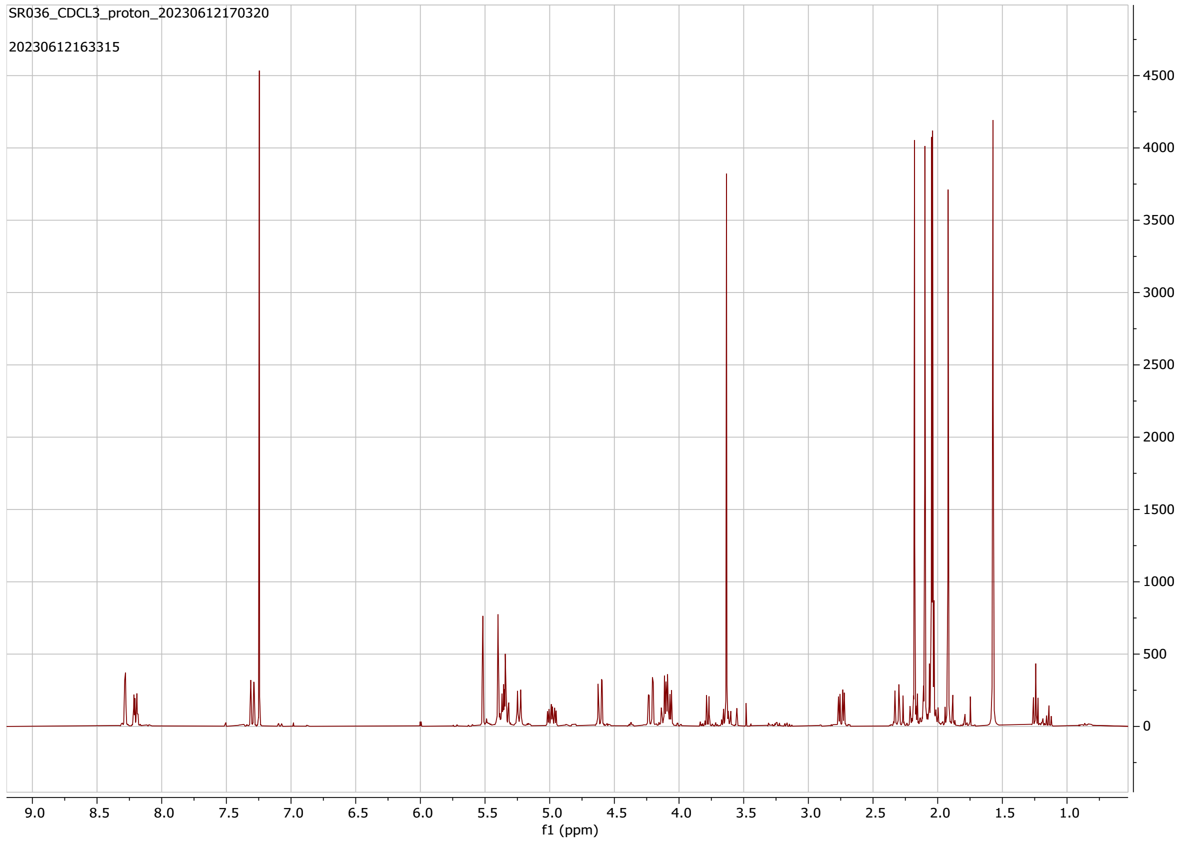

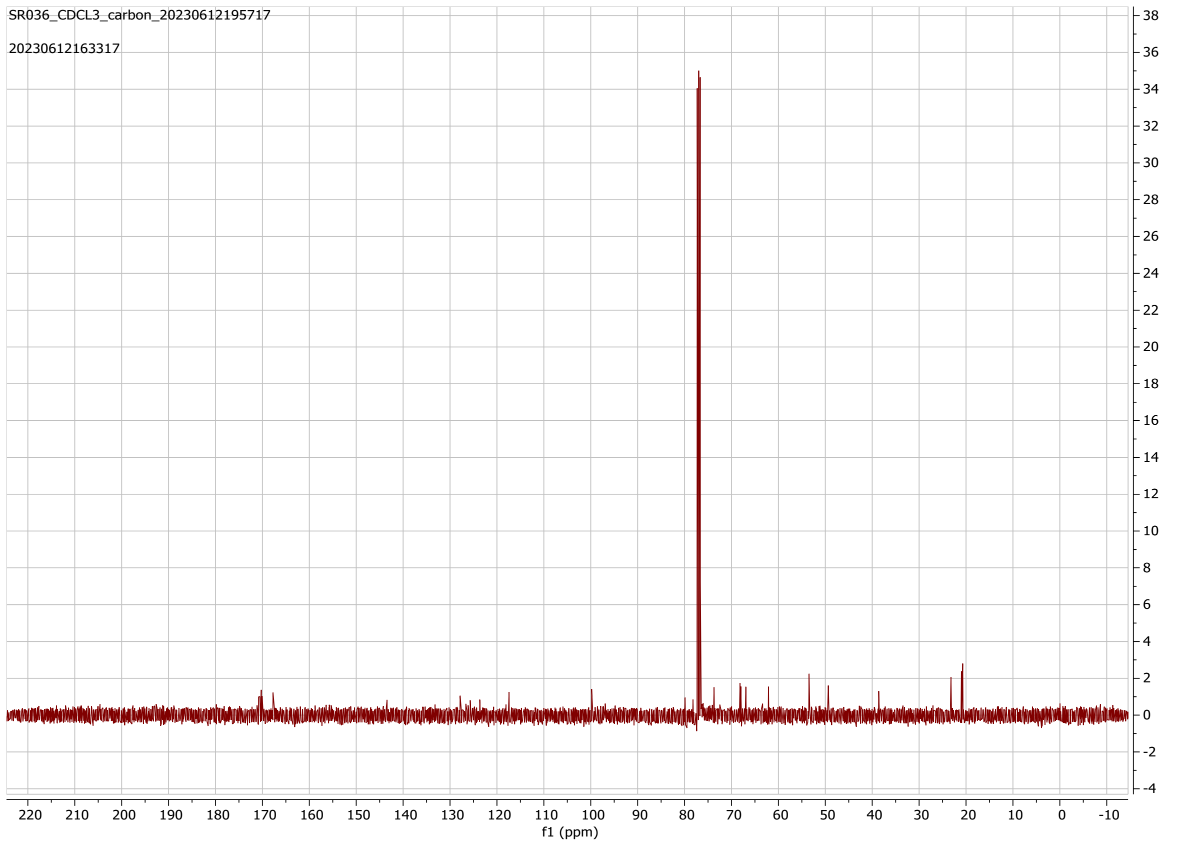
SR036_CDCL3_carbon_20230612195717
20230612163317
f1 (ppm)

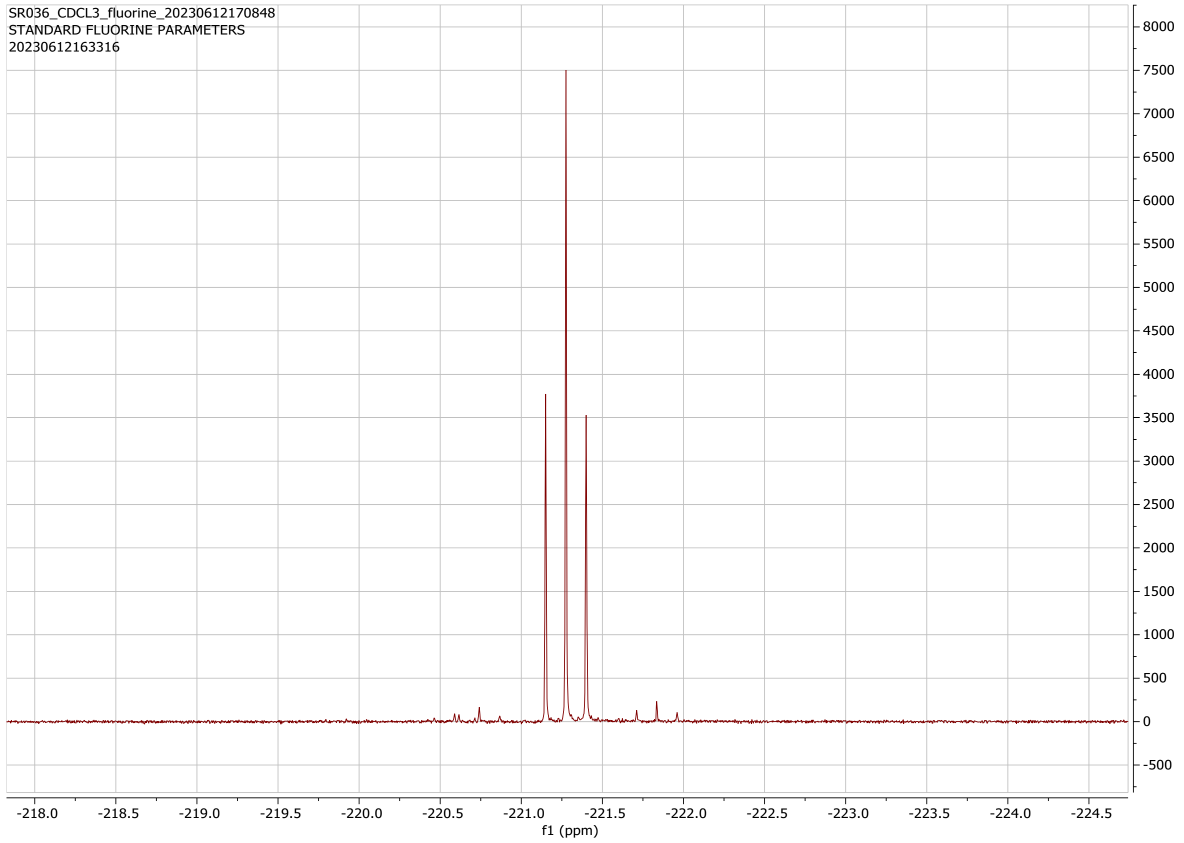
SR036_CDCL3_fluorine_20230612170848
STANDARD FLUORINE PARAMETERS
20230612163316
f1 (ppm)

Methyl (2-*O*-(2-fluoromethyl-4-amino)phenyl-5-acetamido-4,7,8,9-tetra-*O*-acetyl-3,5-dideoxy-D-glycero-α-D-galacto-2-nonulopyranosid)onate (7):

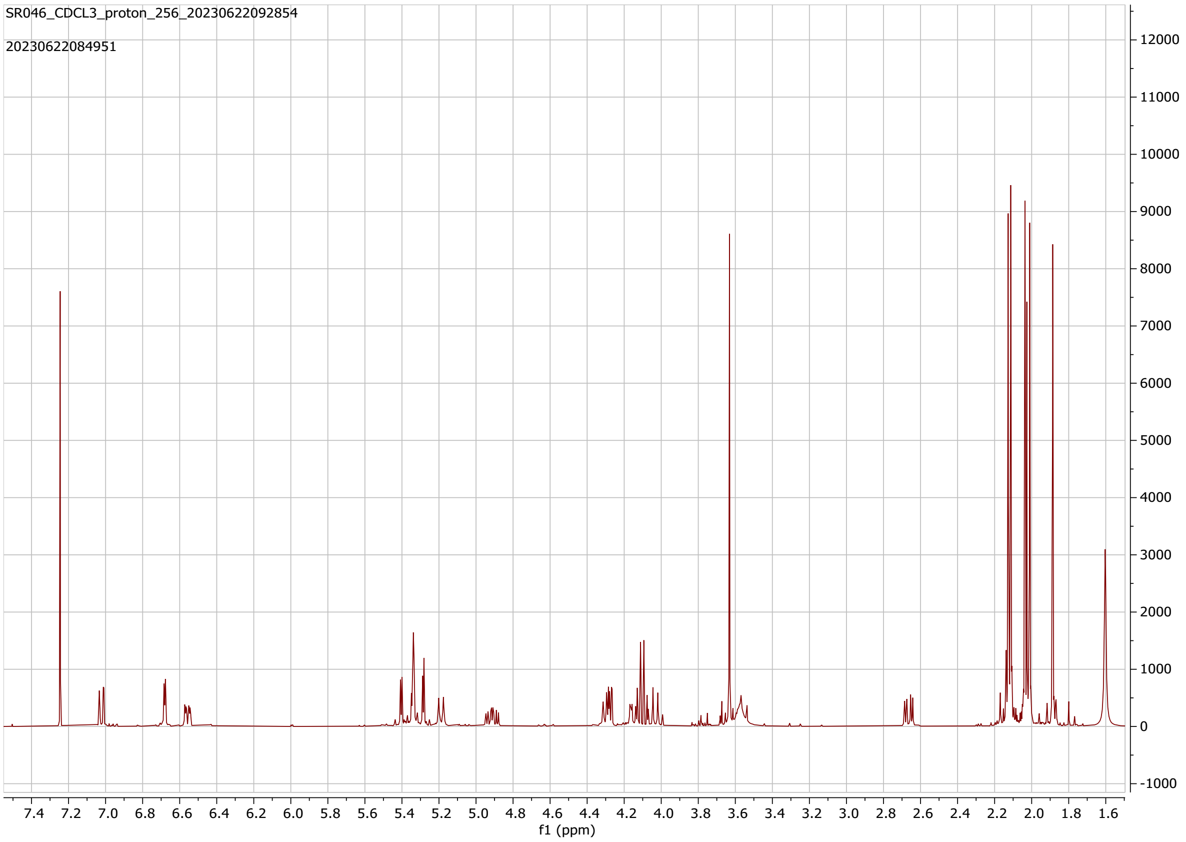

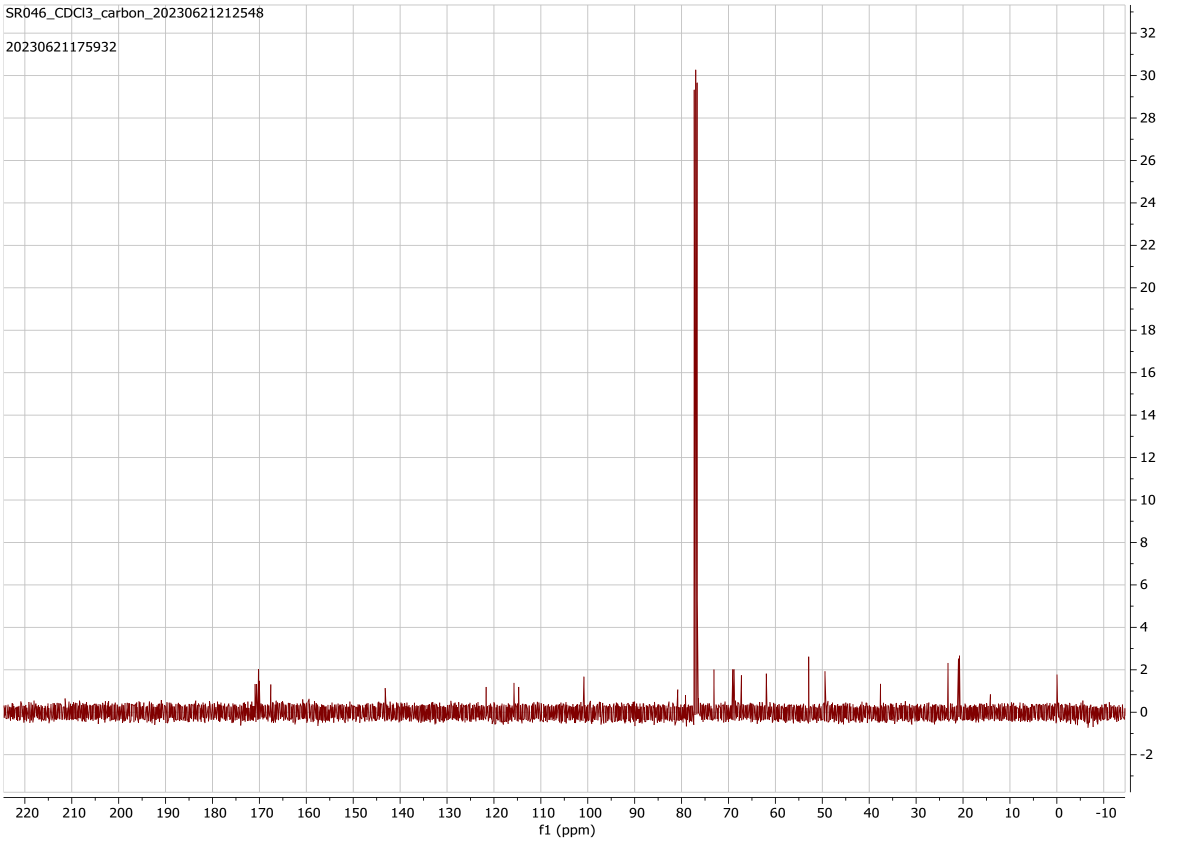

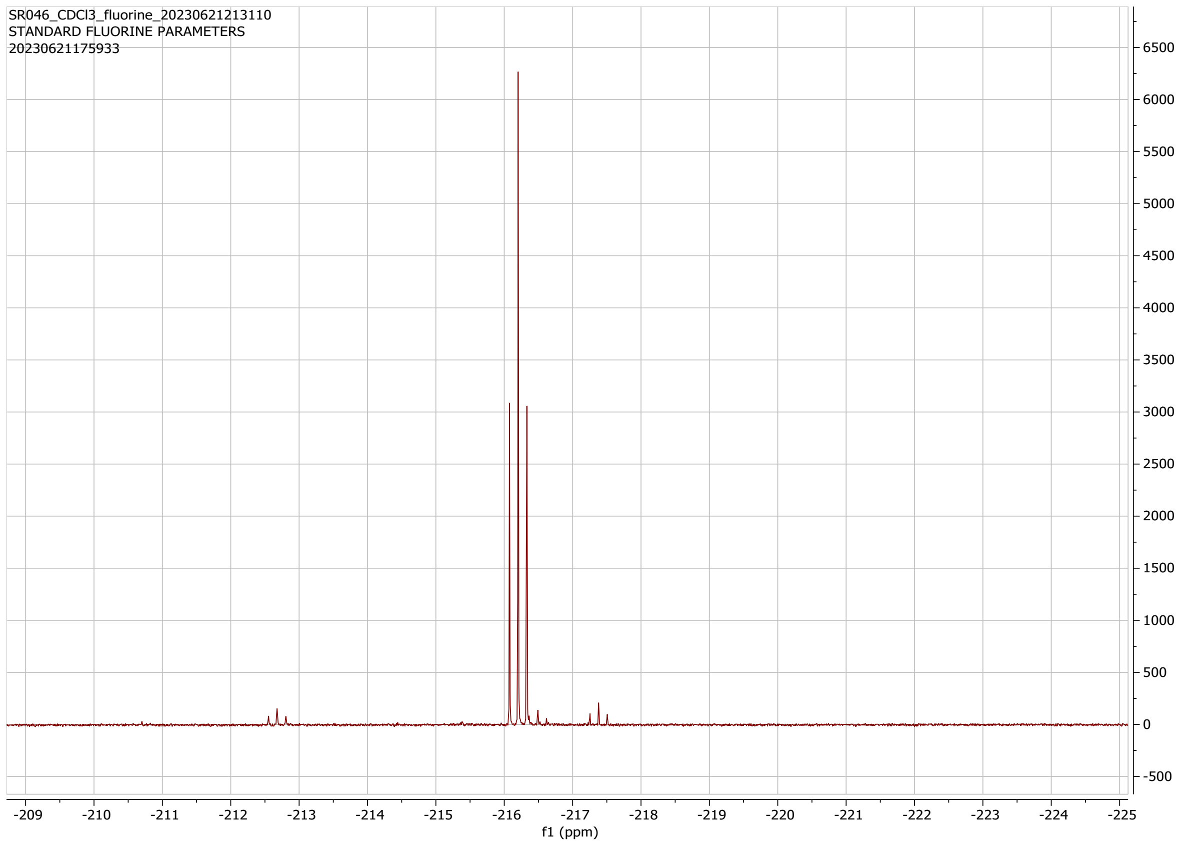

Methyl (2-*O*-(2-difluoromethyl-4-amino)phenyl-5-acetamido-4,7,8,9-tetra-*O*-acetyl-3,5-dideoxy-D-glycero-α-D-galacto-2-nonulopyranosid)onate (8):

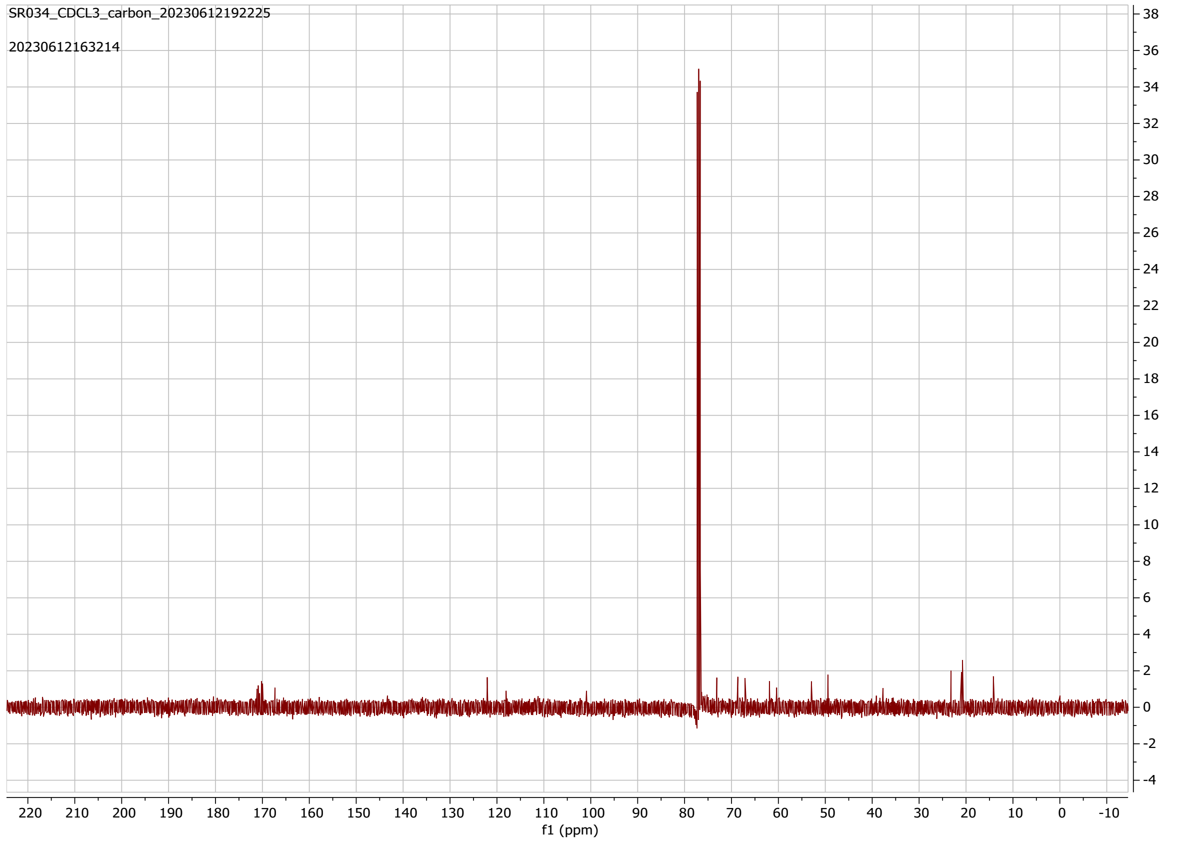

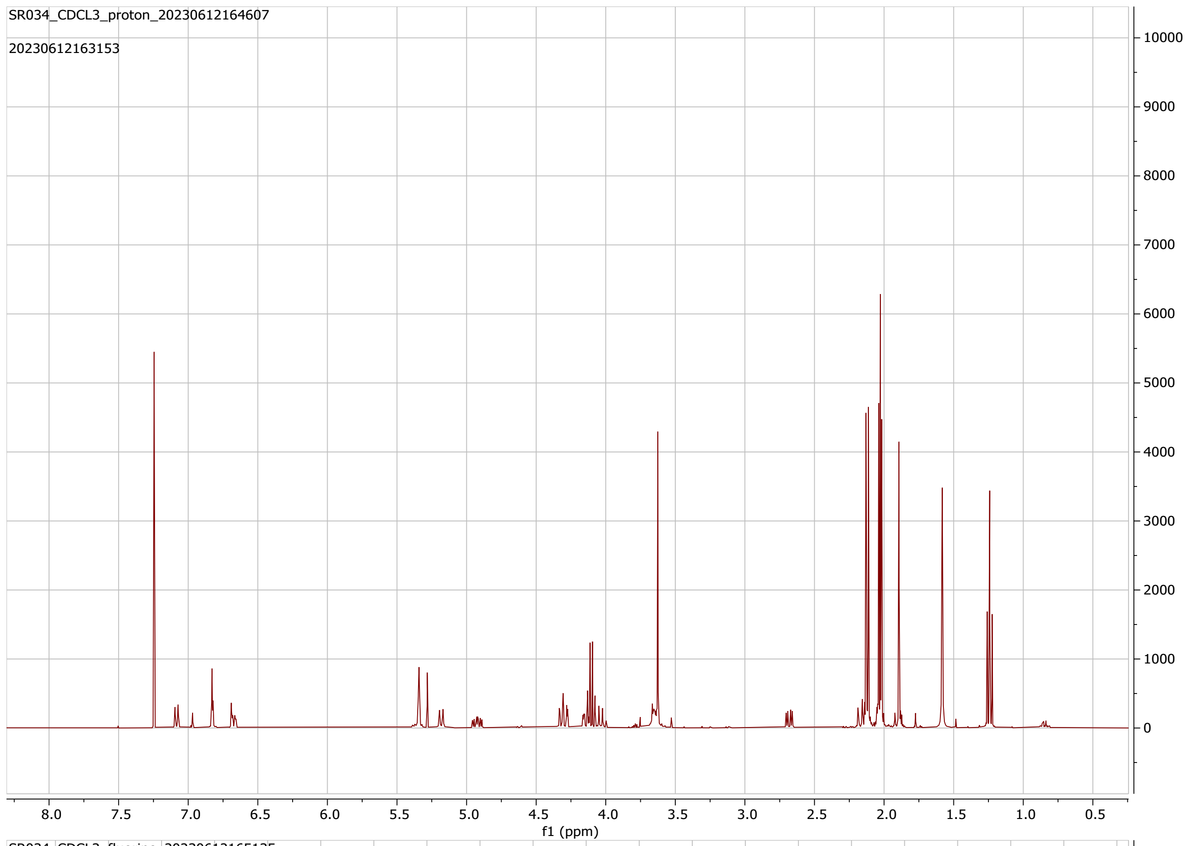
SR034_CDCL3_proton_20230612164607
20230612163153
f1 (ppm)

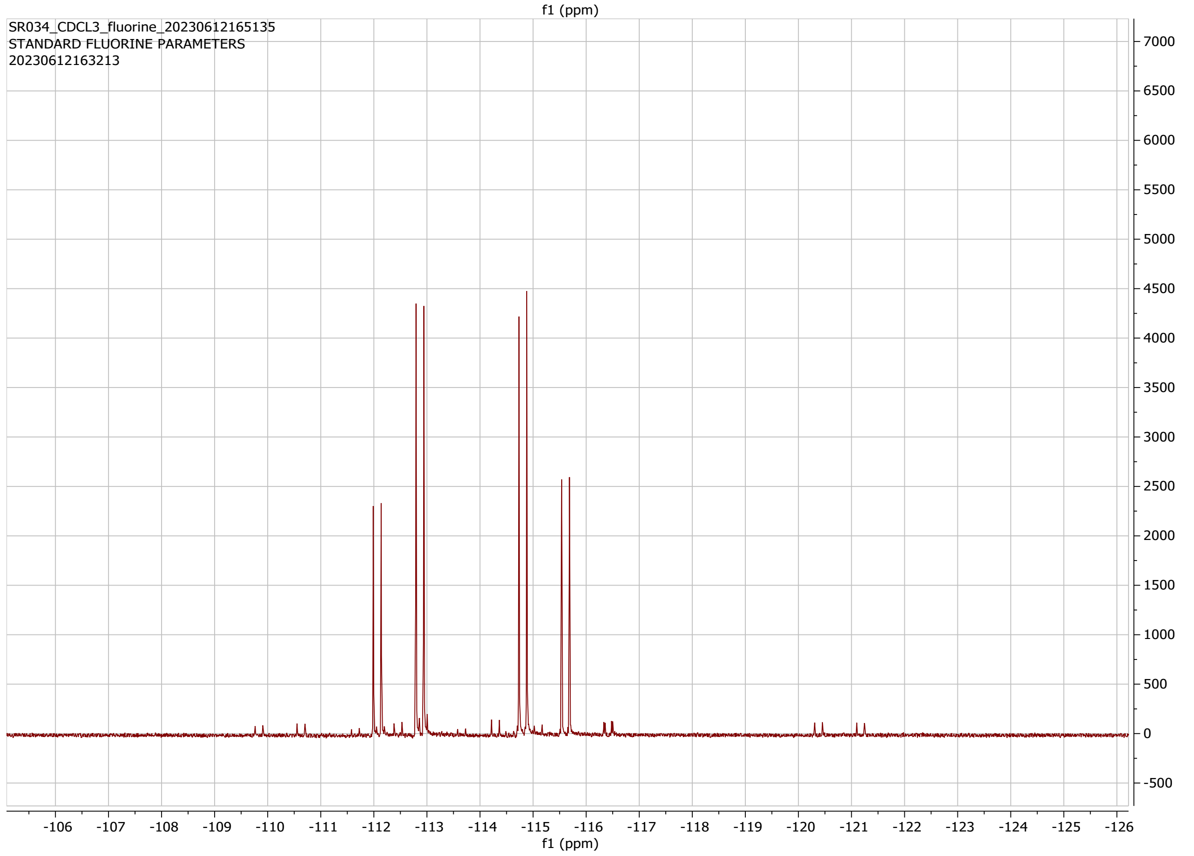
SR034_CDCL3_fluorine_20230612165135
STANDARD FLUORINE PARAMETERS
20230612163213
f1 (ppm)

Methyl (2-*O*-(2-difluoromethyl-4-(2-azidoacetamido)phenyl-5-acetamido-4,7,8,9-tetra-*O*-acetyl-3,5-dideoxy-D-glycero-α-D-galacto-2-nonulopyranosid)onate (9):

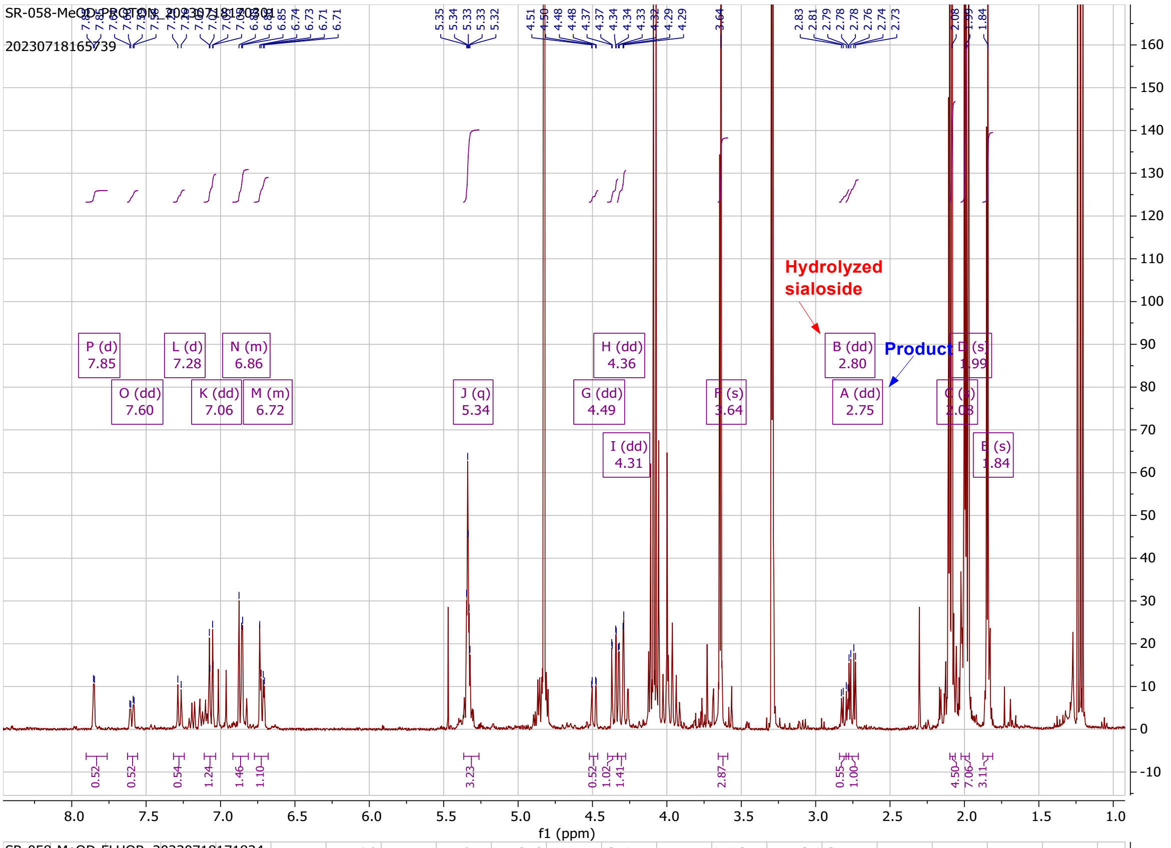

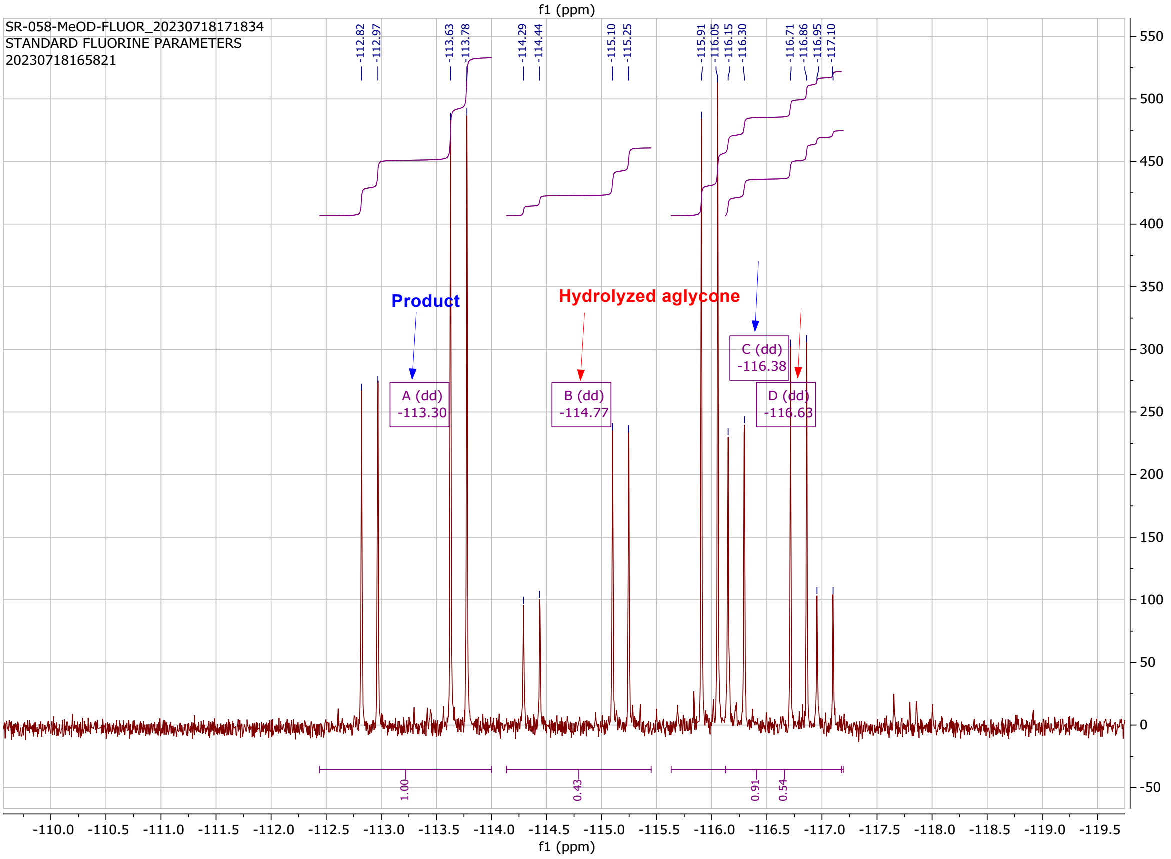

TLC (2:8 DCM [illegible]mic acid):

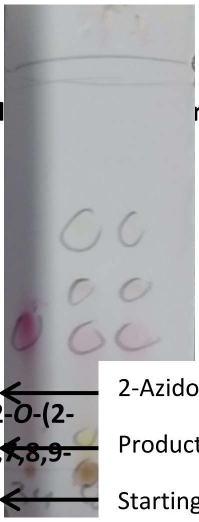

Methyl (2-*O*-(2-[illegible]fluoromethyl-4-(2-azidoacetamido)phenyl-5-acetamido-4,[illegible],8,9-tetra-*O*-acetyl-3,5-dideoxy-D-glycero-α-D-galacto-2-[illegible]nonulopyranosid)onate (10):

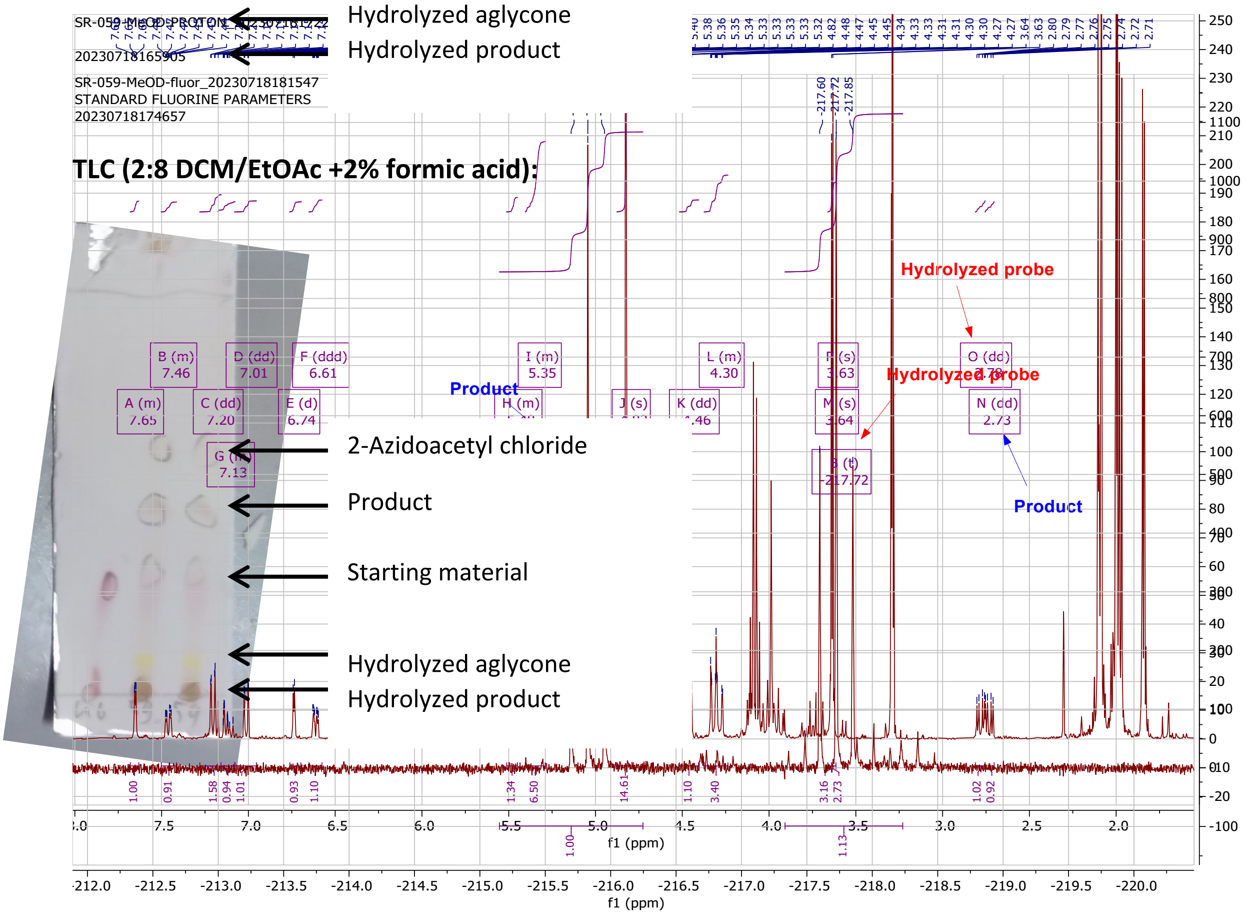

Methyl (2-*O*-(2-difluoromethyl-4-(2-azidoacetamido)phenyl-5-acetamido-3,5-dideoxy-D-glycero-α-D-galacto-2-nonulopyranosid)onate (EA-226):

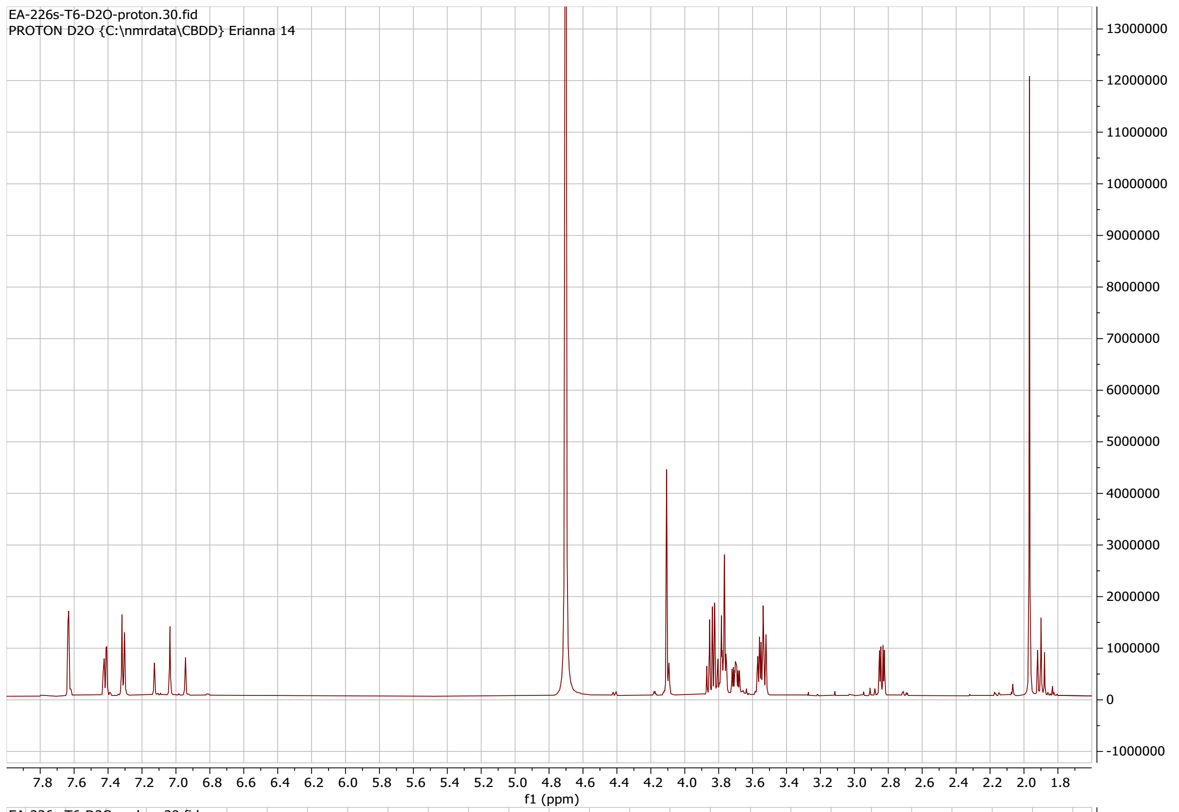
EA-226s-T6-D2O-proton.30.fid
PROTON D2O {C:\nmrdata\CBDD} Erianna 14
f1 (ppm)

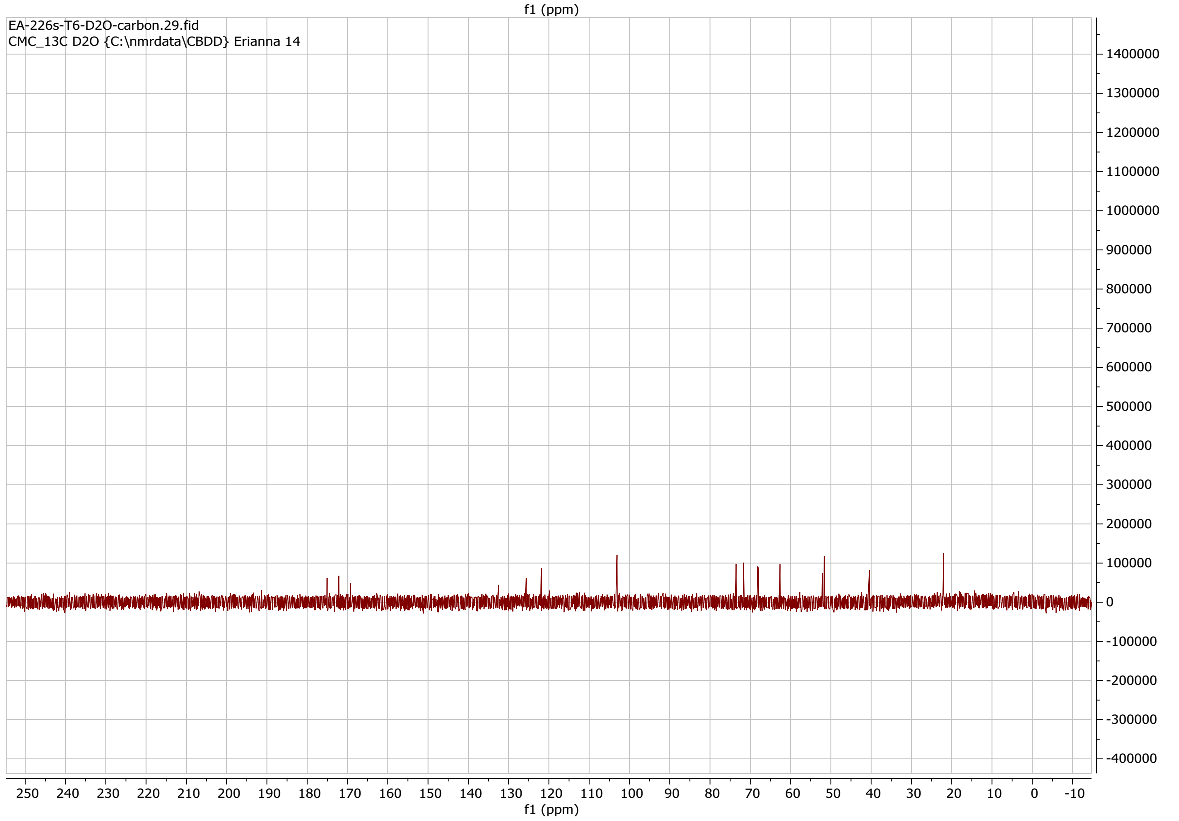
EA-226s-T6-D2O-carbon.29.fid
CMC_13C D2O {C:\nmrdata\CBDD} Erianna 14
f1 (ppm)

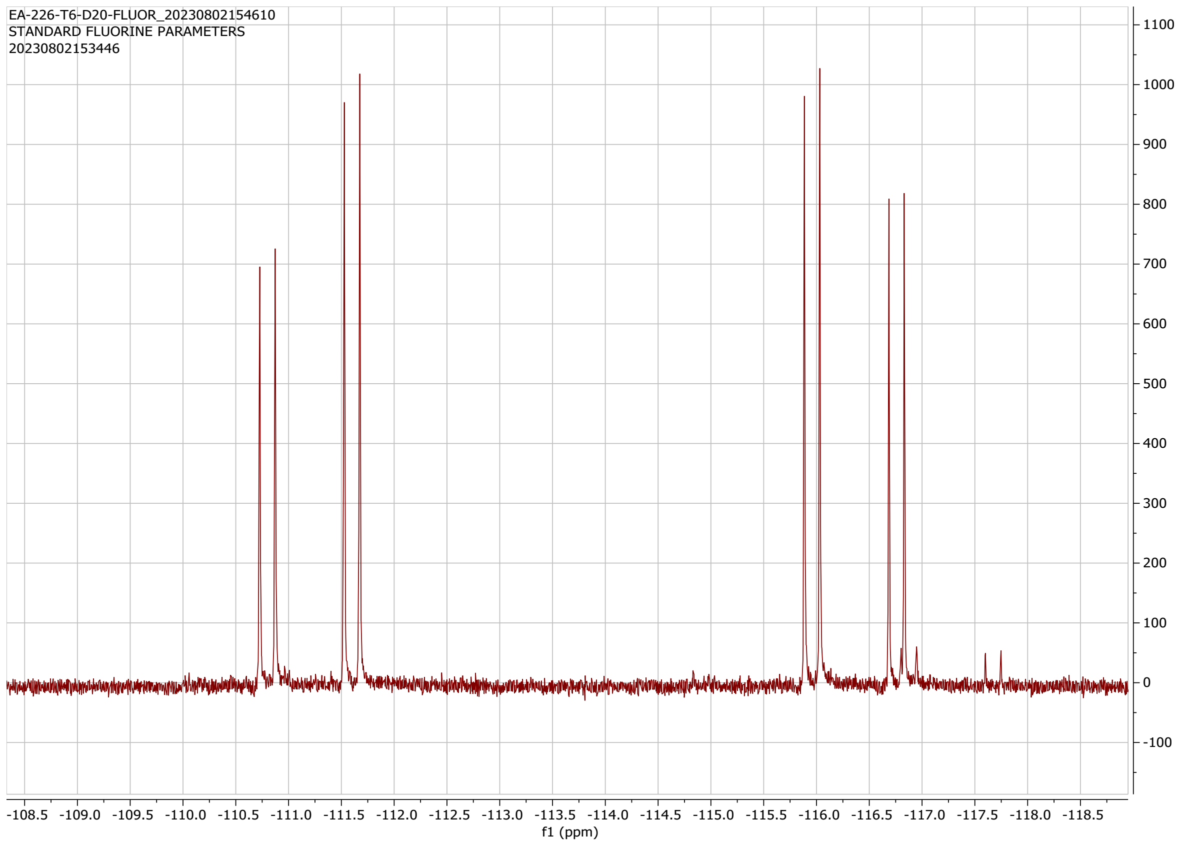

Methyl (2-*O*-(2-difluoromethyl-4-(2-azidoacetamido)phenyl-5-acetamido-3,5-dideoxy-D-glycero-α-D-galacto-2-nonulopyranosid)onate (EA-227):

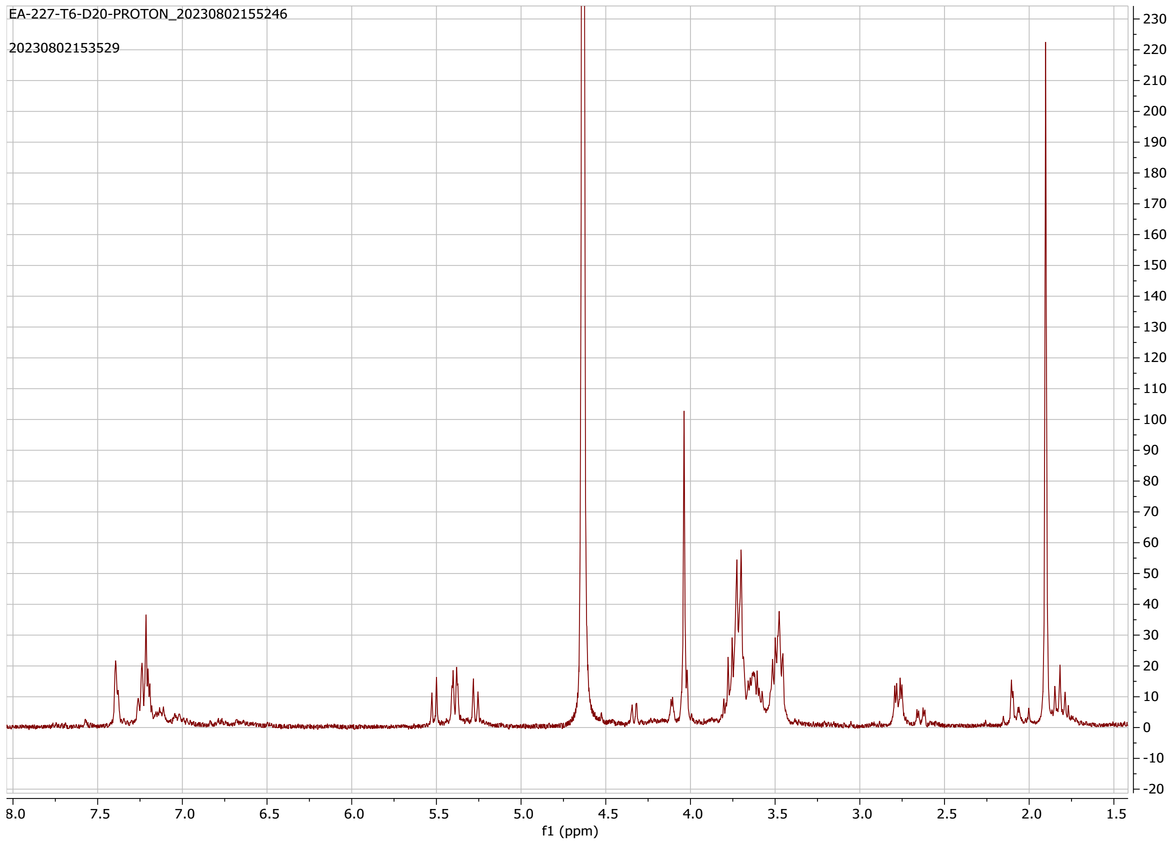

Methyl (2-*O*-(2-fluoromethyl-4-(2-PEG$_4$-biotin-acetamido)phenyl-5-acetamido-3,5-dideoxy-D-glycero-α-D-galacto-2-nonulopyranosid)onate (EA-228):

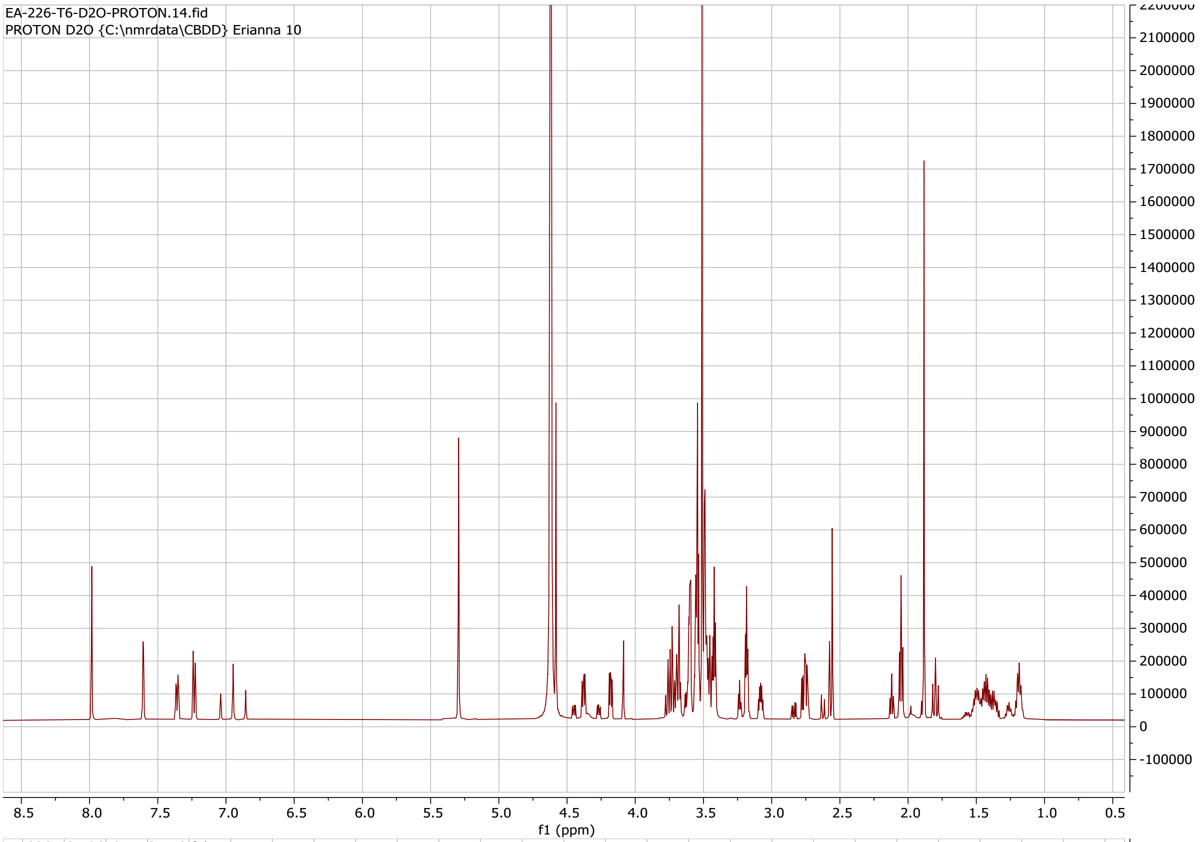

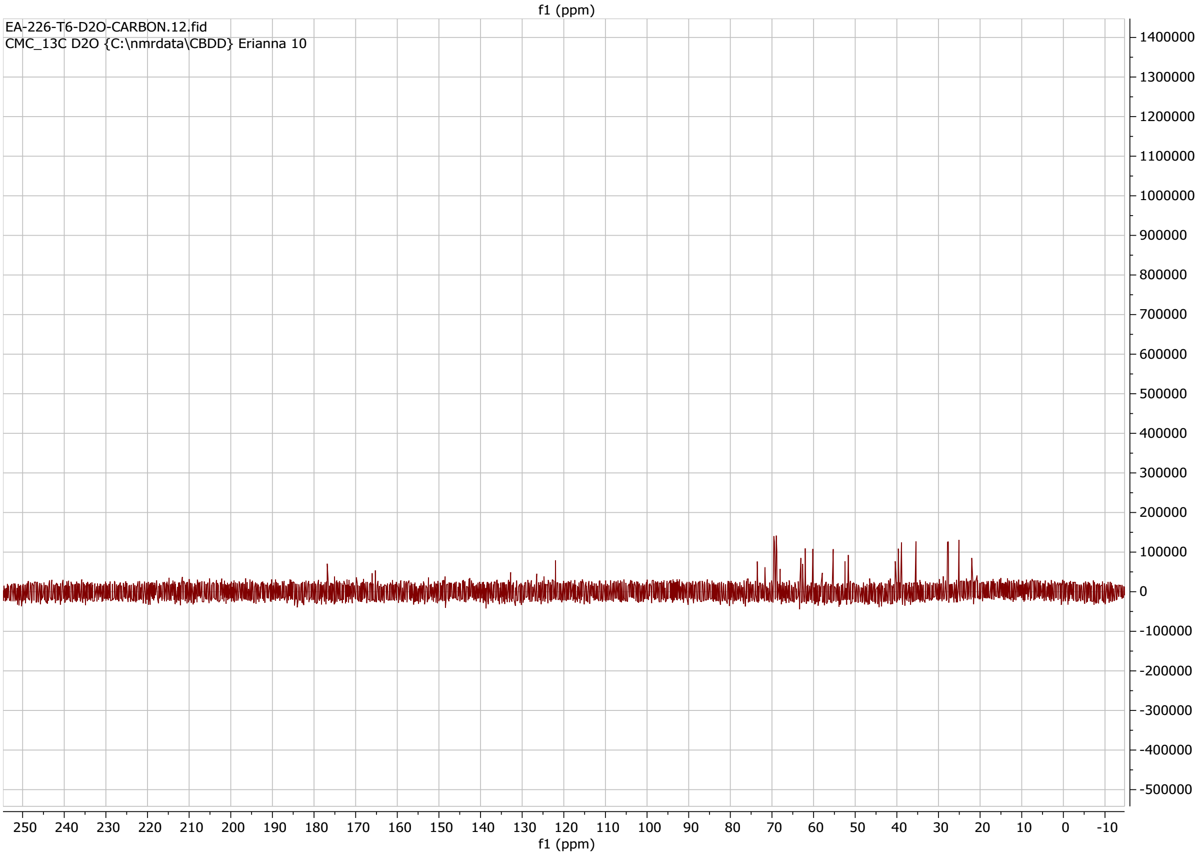

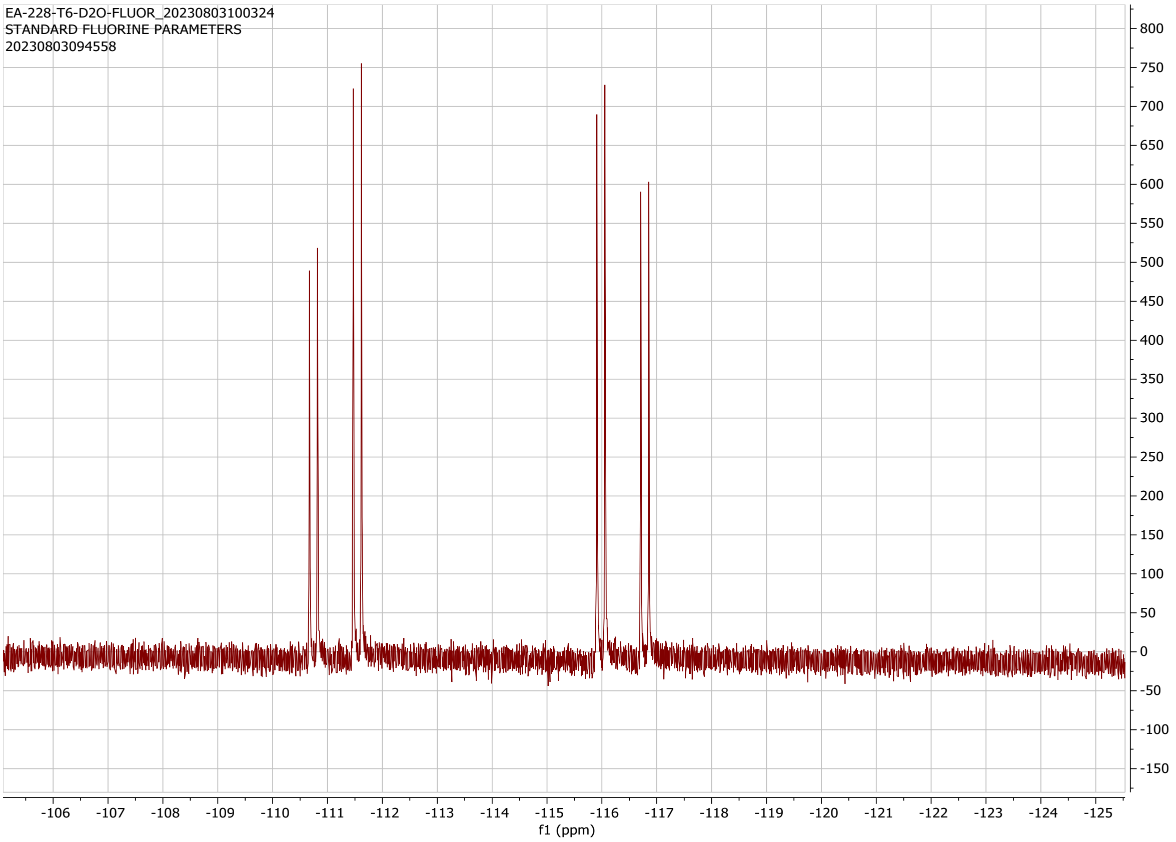

Methyl (2-*O*-(2-difluoromethyl-4-(2-PEG$_4$-biotin-acetamido)phenyl-5-acetamido-3,5-dideoxy-D-glycero-α-D-galacto-2-nonulopyranosid)onate (EA-229):

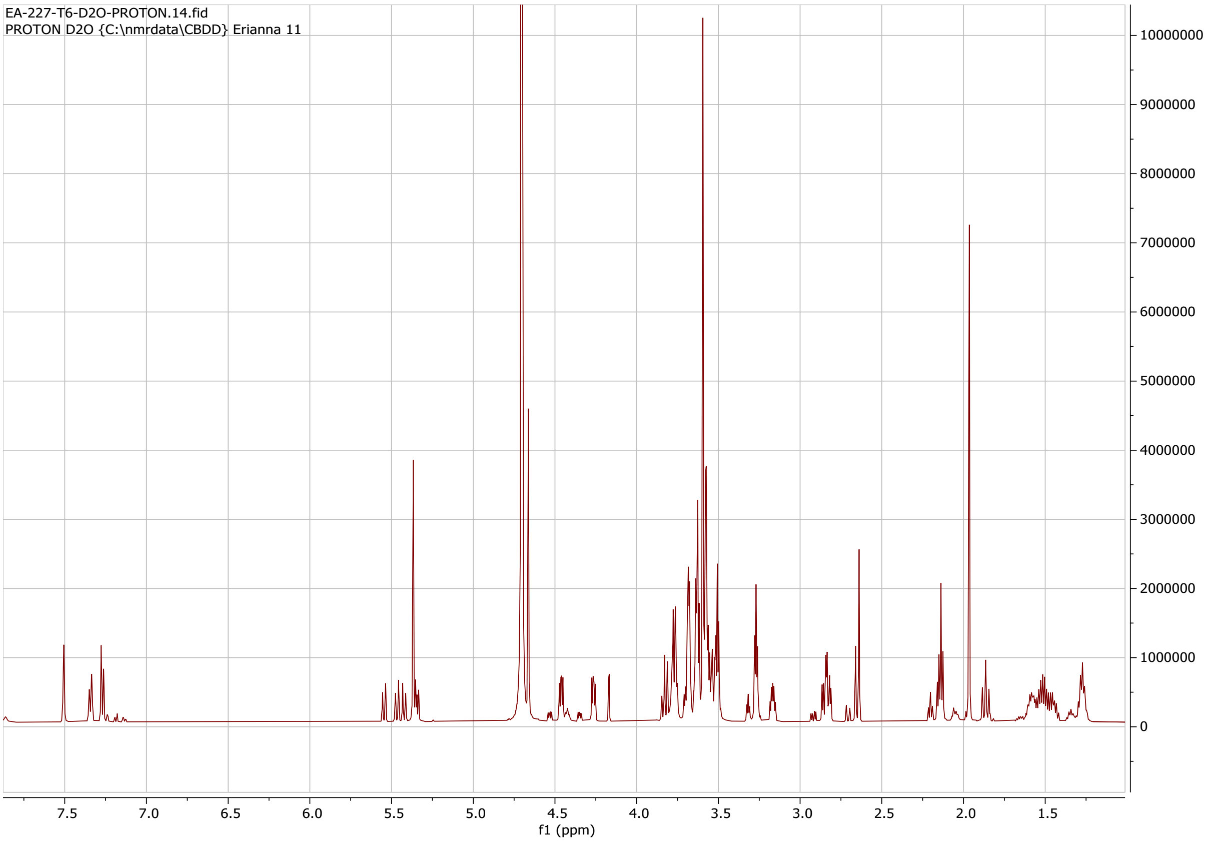

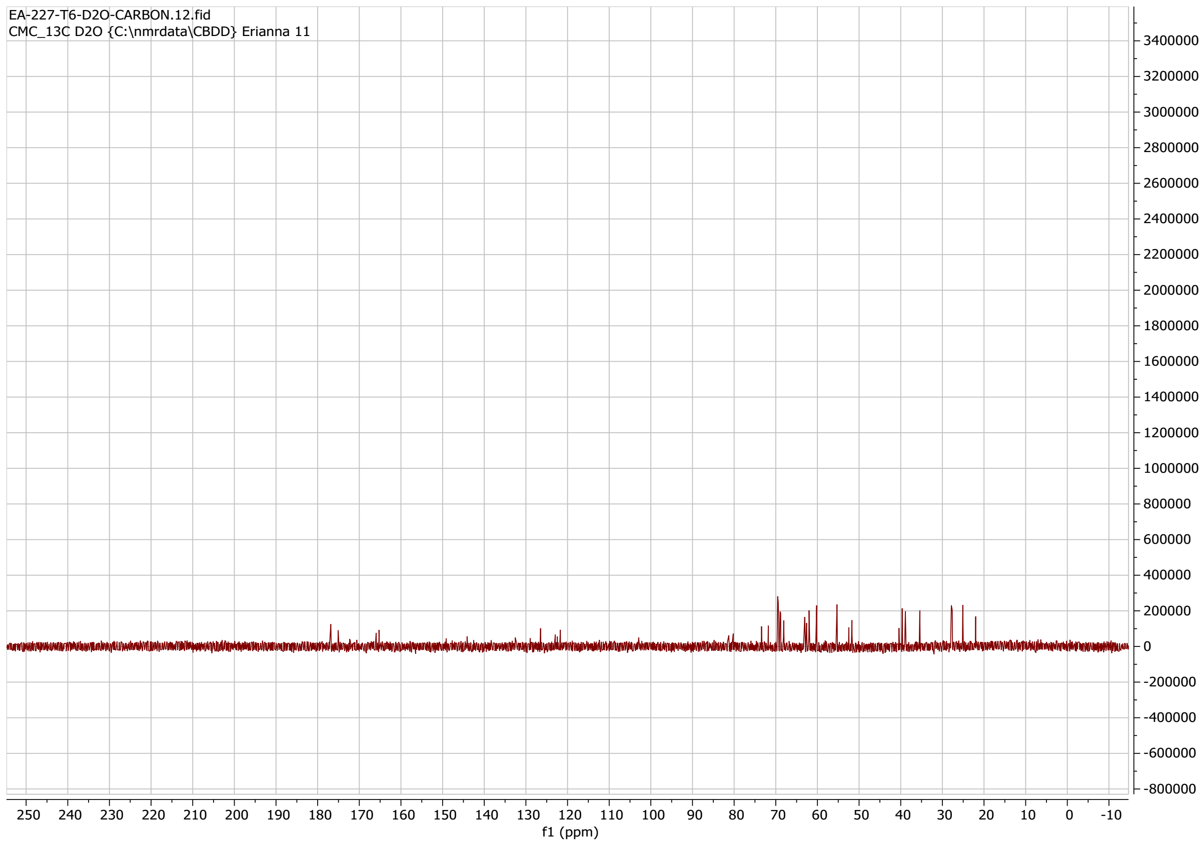
EA-227-T6-D2O-CARBON.12.fid
CMC_13C D2O {C:\nmrdata\CBDD} Erianna 11
f1 (ppm)

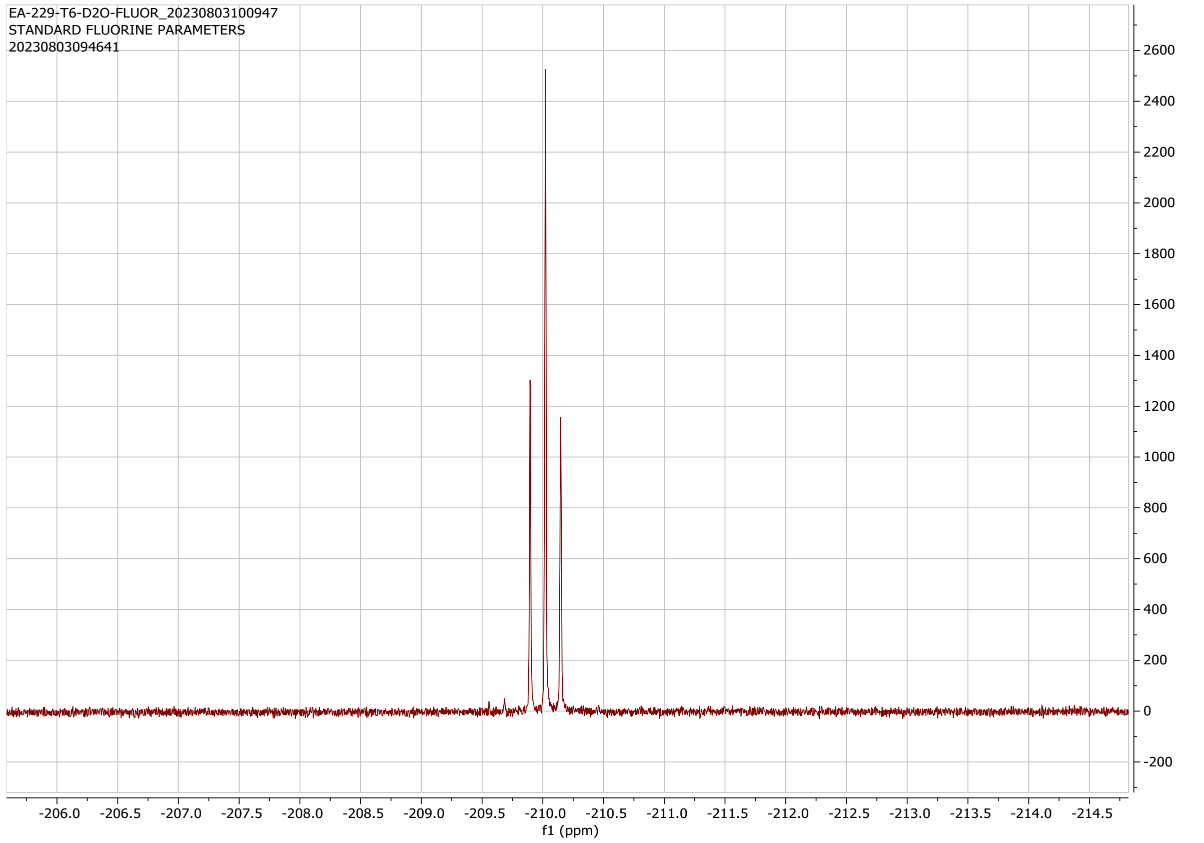
EA-229-T6-D2O-FLUOR_20230803100947
STANDARD FLUORINE PARAMETERS
20230803094641
f1 (ppm)

2. Supporting Information S2. Full lengths gel images.

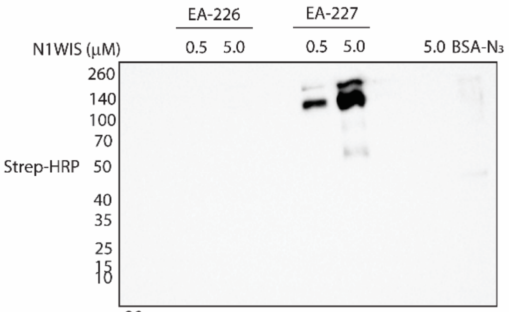

30 s exposure.

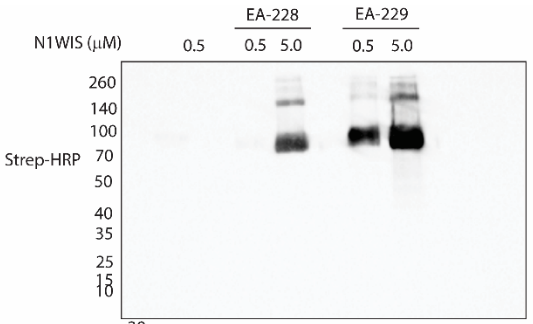

30 s exposure.

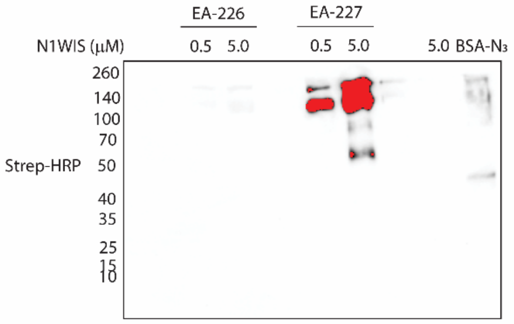

1 minute exposure.

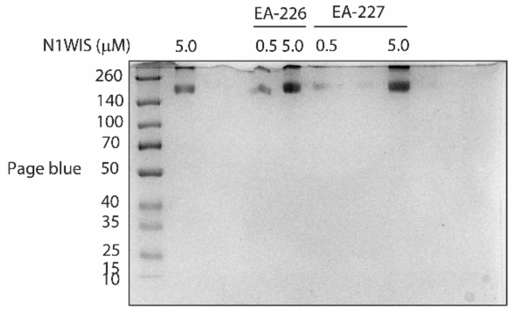

Final concentration of probe 500 uM.
Enzyme was treated with probes for 1 h in bis/tris buffer 50 mM, 4 mM $CaCl_2$, pH 6.
10% SDS gels.

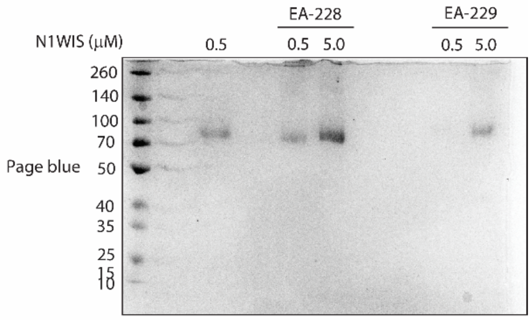

Final concentration of probe 500 uM.
Enzyme was treated with probes for 1 h in bis/tris buffer 50 mM, 4 mM $CaCl_2$, pH 6.
10% SDS gels.

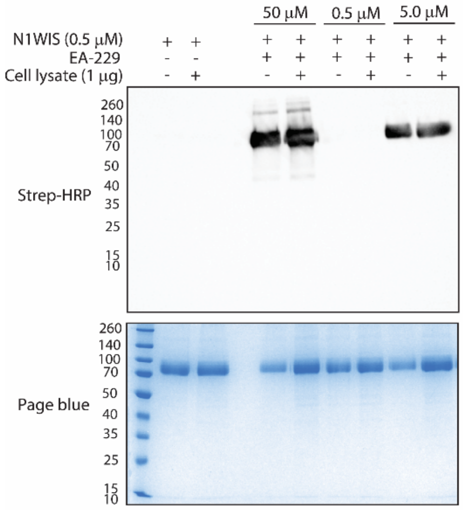

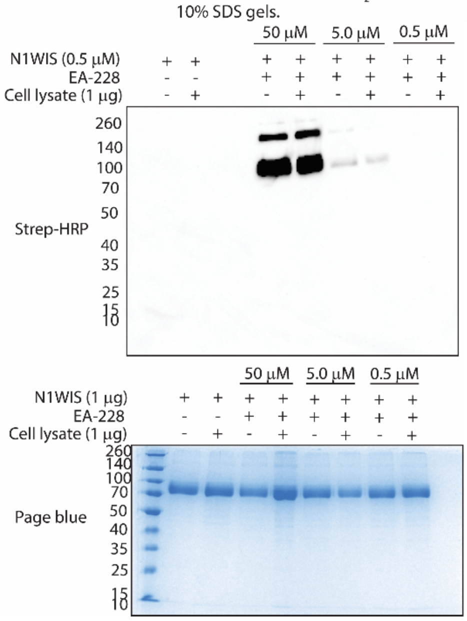

3. **Supporting Information S3.** Inhibition of *Pt*NanH1 and *Pt*NanH2 using DANA (IC_{50}).

The inhibition assay was performed as described in the experimental section. IC_{50} values were calculated using the Onesite-Fit logIC_{50} protocol in GraphPad.

4. **Supporting Information S4.** Fluorometric assay for neuraminidase activity detection of recombinant *Pt*NanH1 and *Pt*NanH2 after 1h incubation with **EA-229**.

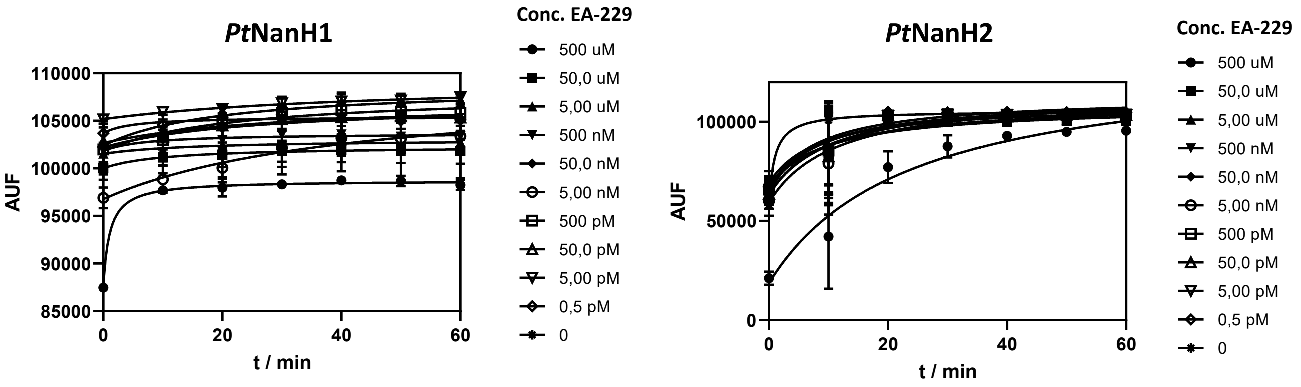

5. **Supporting Information S5.** Gel analysis. Attempts to decrease off-labeling in *P. timonensis* 5C-B1 pellet using **EA-229**.

Incubations were performed in a buffer 50 mM Tris/HCl and 4 mM $CaCl_2$, pH 6). The samples were boiled for 5 minutes in laemmlli buffer (3X), loaded onto 10% SDS gels and run at 90V. Gels were blotted on PDVF membranes, followed by the Western blot protocol using streptavidin-HRP and ECL reagent for visualization.

15 min incubation at RT.

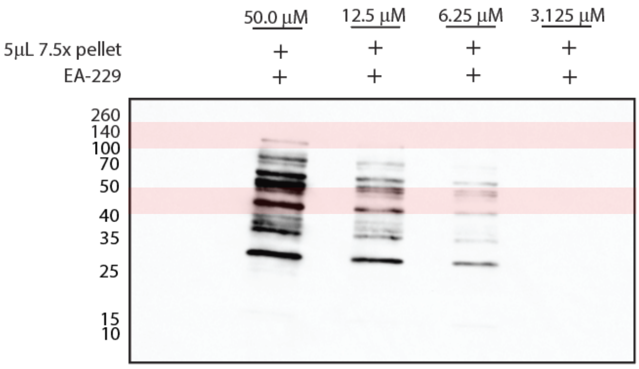

30 minutes incubation at RT.

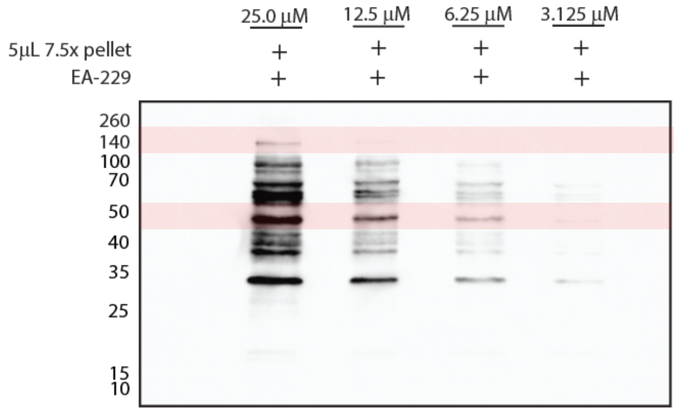

60 minutes incubation at RT.

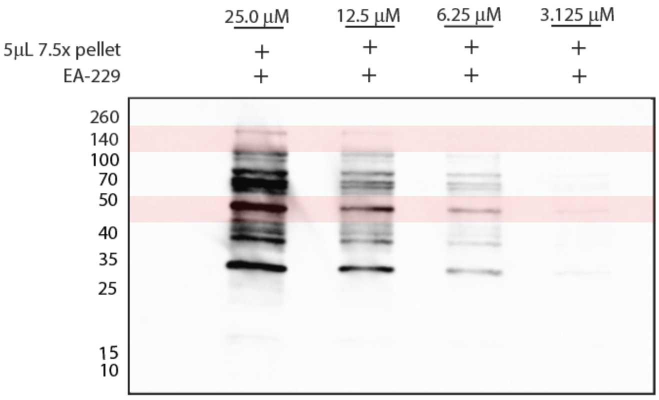

60 minutes incubation at 4°C.

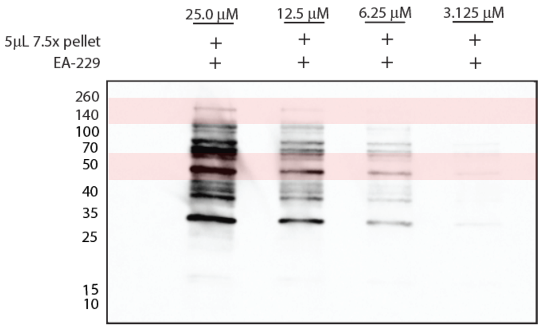

30 minutes incubation at RT, buffer supplemented with Tritonx100.

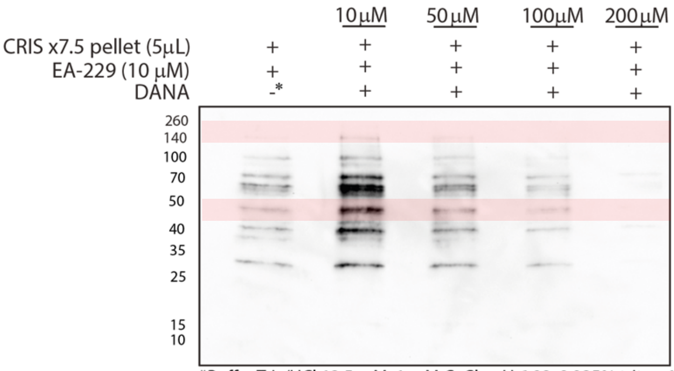

*Buffer Tris/HCl 12.5 mM, 1 mM $CaCl_2$, pH 6.08, 0.025% triton 100.

6. **Supporting Information S6.** Fluorometric assay for neuraminidase activity detection of *P. timonensis* 5C-B1 pellet after incubation with **EA-229**.

2 µL of loose pellet in 48 µL of a buffer 50 mM Tris/HCl and 4 mM $CaCl_2$, pH 6), was used in this assay as the source of neuraminidases. Enzyme solution and probes were incubated for 1h at RT, after which MUNANA (200 µM) was added. Fluorescence read-out (exc.: 320 nm, em.: 490 nm) was measured after 30 minutes of incubation with the fluorescent substrate at RT.

7. **Supporting Information S7.** Fluorescence microscopy images of *P. timonensis* CRIS 5C-B1 fluorescently labeled using **EA-227** followed by CuAAC reaction with alkyne-AF_{488} (488 channel and light channel).

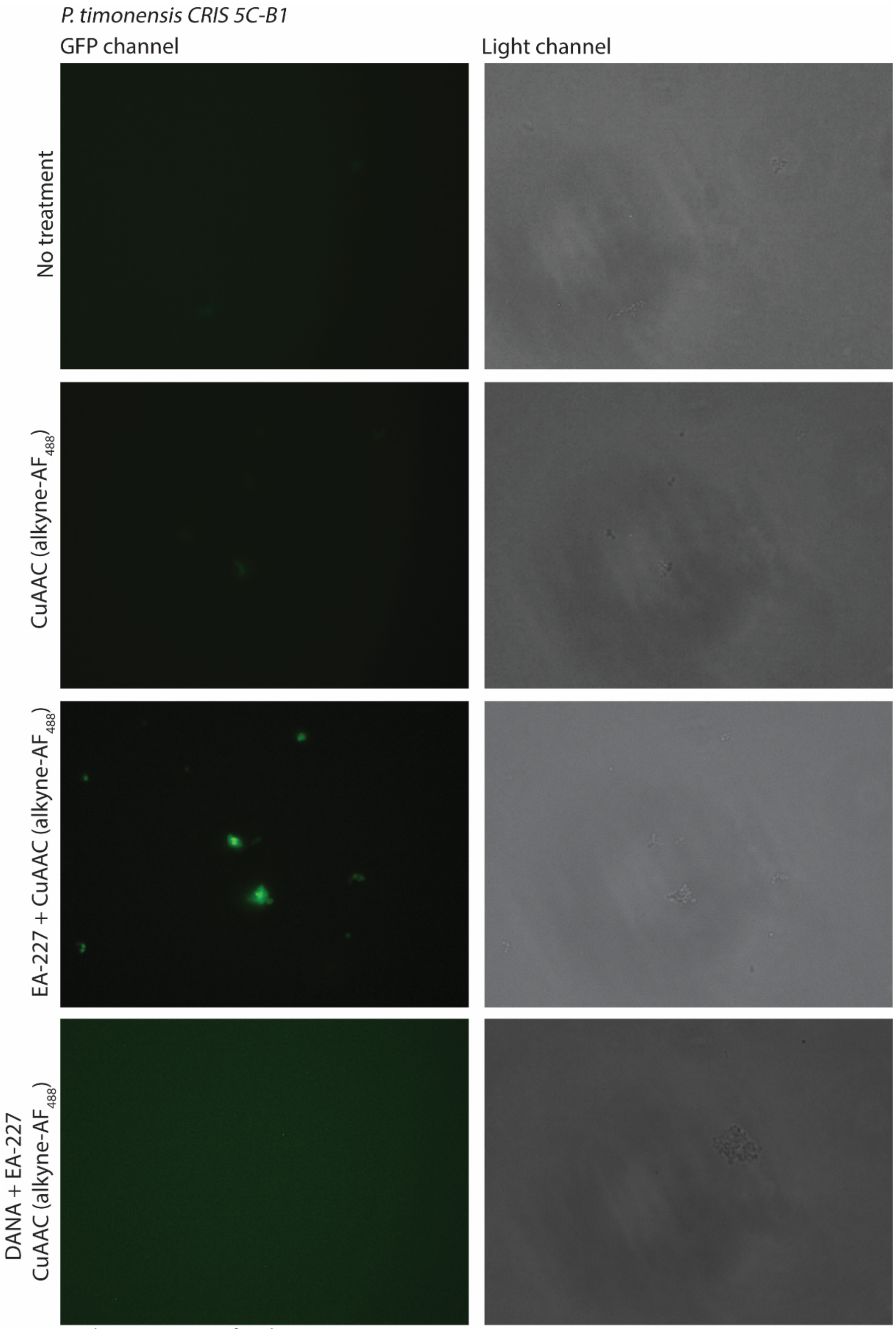

Final concentration of probe was 20 μM.
Invitrogen™ EVOS™ M5000 Imaging System100X, Light exposure 0.5.

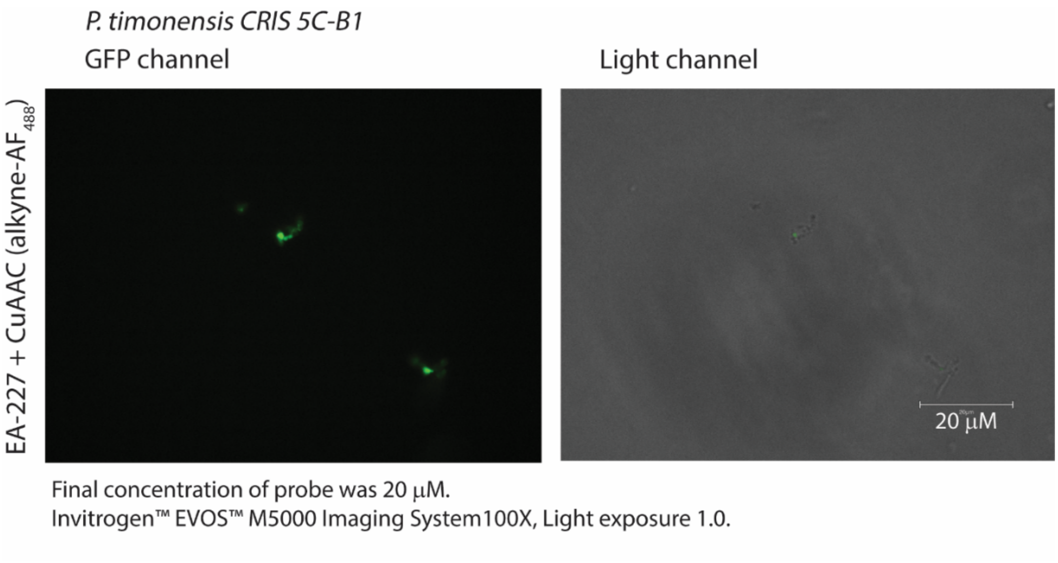

Supporting Information S8. 4-MUNANA assay for the evaluation of the enzymatic activity of *Pt*NanH1 and *Pt*NanH2 in different buffers.

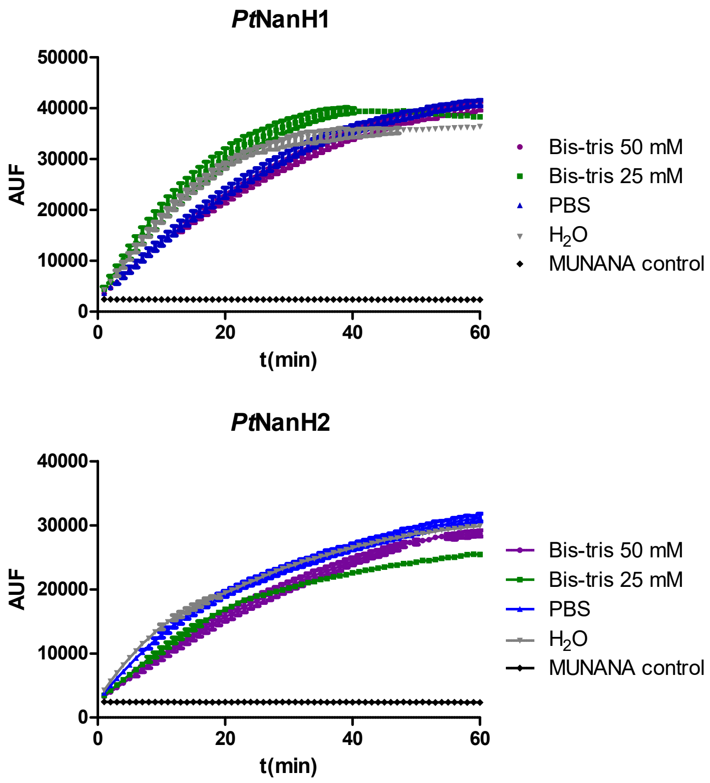

Supplement: CB-006-D5CB00170F-s001 [file CB-006-D5CB00170F-s001.pdf]
